# Supplementary material for: Altered microRNA Transcriptome in Cultured Human Liver Cells upon Infection with Ebola Virus
Source: Int J Mol Sci. 2021 Apr 6;22(7):3792. doi: 10.3390/ijms22073792 (PMC8038836; doi:10.3390/ijms22073792)
Supplement: Supplementary file 1 [file ijms-22-03792-s001.zip › Supplementary_File/C_ GO_Analysis_Results/16-30nt_go_Mayinga-96h-Huh7_vs_Control-96h-Huh7_up.mature_mirna_targets/BP_result(Human).html]

| GO.ID | Term | Ontology | Count | Pop.Hits | List.Total | Pop.Total | Fold.Enrichment | Pvalue | FDR | Enrichment.Score | Gene.Ratio | GENES |
| --- | --- | --- | --- | --- | --- | --- | --- | --- | --- | --- | --- | --- |
| GO:0045935 | positive regulation of nucleobase-containing compound metabolic process | Biological process | 62 | 1742 | 393 | 17653 | 1.59870933062228 | 0.000136492817664931 | 0.339235039690401 | 3.86489020086172 | 0.157760814249364 | NCOA1//BAK1//XBP1//ATRX//WIZ//KLF2//USP16//CKS2//F2R//FBXW11//EBF3//SMAD5//MITF//NPM1//CRLF3//ARNTL2//RNF4//RORA//NCOA4//FUBP3//PIAS2//CHURC1//HAND2//CREB5//CALCR//NPPC//NCBP2//EREG//TIMP2//PRKAA2//TFRC//PSMC6//FSTL3//KAT7//E2F5//CRTC1//GABPB1//NR6A1//HOXA9//IL2//NFYC//PAX7//PBX3//TRIAP1//CDK12//PLAGL1//RARB//RFX4//ALX4//HIF3A//SMO//TRPS1//YES1//ZC3H12A//MIXL1//CCNC//SUB1//GTPBP1//TRIM71//QKI//PCK1//NMD3// |
| GO:0000041 | transition metal ion transport | Biological process | 10 | 110 | 393 | 17653 | 4.08350682396484 | 0.000172991617674012 | 0.339235039690401 | 3.76197494014357 | 0.0254452926208651 | SLC30A5//ATP7A//SFXN1//TRPC3//TRPC5//STEAP2//B2M//TFRC//ATP6V0E1//SLC30A2// |
| GO:0048172 | regulation of short-term neuronal synaptic plasticity | Biological process | 4 | 15 | 393 | 17653 | 11.9782866836302 | 0.000271796158808304 | 0.339235039690401 | 3.56575668527882 | 0.0101781170483461 | GRIK2//SHISA6//SYP//SHISA9// |
| GO:0007049 | cell cycle | Biological process | 65 | 1898 | 393 | 17653 | 1.53830736519223 | 0.000274661071534349 | 0.339235039690401 | 3.56120288996372 | 0.165394402035623 | TRIM71//EIF4E//CRLF3//MCM10//CUL2//DHFR//GFI1//FBXW11//YWHAG//CEP135//DYNLT1//ATRX//USP16//WDHD1//TUBB2B//CLTCL1//RAB6C//MAP9//E2F5//NF2//CDK12//BAK1//CDKL1//NPM1//TRIAP1//PLAGL1//DHCR24//HEPACAM//PRKAA2//THBS1//SPC24//DYNC1LI2//CKS2//TAOK3//CDC14A//HEPACAM2//C14ORF39//SYCE1//ASZ1//EREG//MAP3K8//STK24//USP51//WIZ//PSMA8//PSMA1//PSMC6//CCNC//PPP1R13B//BTG3//TIMP2//RBM7//EXD1//NPPC//RNF4//APEX1//CCSAP//TPRA1//CLTA//APEX2//TACC1//UBE2I//CDK2AP1//RASSF4//RASSF2// |
| GO:0006366 | transcription by RNA polymerase II | Biological process | 78 | 2405 | 393 | 17653 | 1.45681865071178 | 0.000342928367183171 | 0.339235039690401 | 3.46479658837132 | 0.198473282442748 | KLF2//ZHX1//IRX2//DR1//CUX2//NR6A1//GFI1//SMAD5//MITF//DLL4//H2AFY2//PURB//RARB//SMO//TRPS1//UBE2I//NCOA1//NCBP2//SNRPF//SRSF11//FSTL3//USP16//ZNF273//SUB1//WDHD1//FOXP4//CKS2//CERS6//GABPB1//ALX3//NFYC//NPM1//QRICH1//THAP10//ARNTL2//RFX4//ZNF704//HIF3A//SP4//ZNF37A//SLTM//CREB5//RORA//TAF11//CCNC//CDK12//XBP1//TMEM100//CTDSPL2//C5AR1//PSMC6//KAT7//E2F5//CRTC1//EBF3//HOXA9//IL2//PAX7//PBX3//CRLF3//TRIAP1//PLAGL1//ATRX//RNF4//ALX4//YES1//ZC3H12A//MIXL1//FUBP3//PIAS2//HAND2//PCK1//PSMA8//PSMA1//CUL2//TAF5L//YBX2//BTF3// |
| GO:0019219 | regulation of nucleobase-containing compound metabolic process | Biological process | 126 | 4328 | 393 | 17653 | 1.30770343299798 | 0.00038130443064788 | 0.339235039690401 | 3.41872814831997 | 0.320610687022901 | KPNA1//DHFR//GFI1//KLF2//ZHX1//IRX2//DR1//CUX2//NR6A1//SMAD5//MITF//DLL4//H2AFY2//PURB//RARB//SMO//TRPS1//UBE2I//RBM7//NCOA1//ATP7A//PCGF3//KAT7//ZNF597//ZNF558//ZNF320//KDM2A//ACAD8//TAF5L//ZNF544//APEX1//ZNF699//AFF3//NFYC//YBX2//ATRX//COMMD8//PRKAA2//ARNTL2//REXO4//RORA//TAF11//BTF3//ZNF37A//ZNF75D//CSDE1//ZNF329//SAP30L//LZTR1//ZNF251//FSTL3//USP16//ZNF273//SUB1//WDHD1//FOXP4//CKS2//CERS6//GABPB1//ALX3//NPM1//QRICH1//THAP10//RFX4//ZNF704//HIF3A//SP4//SLTM//CREB5//BAK1//XBP1//G3BP2//NF2//USP51//WIZ//F2R//FBXW11//EBF3//CRLF3//RNF4//NCOA4//FUBP3//PIAS2//CHURC1//HAND2//ZNF488//EREG//PRAMEF19//TMEM100//CTDSPL2//NPPC//CALCR//THBS1//NCBP2//NOD1//ZC3H12A//CRTC1//CDK12//PSMA8//PSMA1//PSMC6//TIMP2//TFRC//E2F5//HOXA9//IL2//PAX7//PBX3//TRIAP1//PLAGL1//ALX4//YES1//MIXL1//CCNC//C14ORF39//RIPK3//CD40LG//NOVA2//GTPBP1//TRIM71//QKI//PCK1//CUL2//NMD3//RAP2C//RAD52// |
| GO:0051254 | positive regulation of RNA metabolic process | Biological process | 54 | 1523 | 393 | 17653 | 1.59264809811892 | 0.000424699514434153 | 0.339235039690401 | 3.37191823554497 | 0.137404580152672 | NCOA1//XBP1//KLF2//USP16//CKS2//F2R//FBXW11//EBF3//SMAD5//MITF//NPM1//CRLF3//ARNTL2//RNF4//RORA//NCOA4//FUBP3//PIAS2//CHURC1//HAND2//CREB5//NCBP2//PSMC6//FSTL3//KAT7//E2F5//CRTC1//GABPB1//NR6A1//HOXA9//IL2//NFYC//PAX7//PBX3//TRIAP1//CDK12//PLAGL1//ATRX//RARB//RFX4//ALX4//HIF3A//SMO//TRPS1//YES1//ZC3H12A//MIXL1//CCNC//SUB1//GTPBP1//TRIM71//QKI//PCK1//NMD3// |
| GO:0045944 | positive regulation of transcription by RNA polymerase II | Biological process | 43 | 1132 | 393 | 17653 | 1.70627096089697 | 0.000428560573603538 | 0.339235039690401 | 3.36798778535677 | 0.10941475826972 | NCOA1//XBP1//KLF2//PSMC6//SUB1//PCK1//SMAD5//ATRX//HAND2//FSTL3//USP16//KAT7//E2F5//CRTC1//EBF3//GABPB1//NR6A1//HOXA9//IL2//MITF//NFYC//NPM1//PAX7//PBX3//CRLF3//TRIAP1//CDK12//PLAGL1//ARNTL2//RARB//RFX4//RNF4//ALX4//RORA//HIF3A//SMO//TRPS1//YES1//ZC3H12A//MIXL1//CCNC//FUBP3//PIAS2// |
| GO:0019882 | antigen processing and presentation | Biological process | 14 | 221 | 393 | 17653 | 2.84552059226509 | 0.000451443938668285 | 0.339235039690401 | 3.34539617367537 | 0.0356234096692112 | B2M//PSMA8//PSMA1//PSMC6//THBS1//NOD1//IFI30//CLTA//DYNC1LI2//HLA-DPB1//CTSF//CLEC4M//CD209//CD1D// |
| GO:0006139 | nucleobase-containing compound metabolic process | Biological process | 164 | 5967 | 393 | 17653 | 1.23456448976581 | 0.00055743848210072 | 0.357639997255935 | 3.25380305358627 | 0.417302798982188 | KPNA1//KAT7//MCM10//DHFR//GFI1//KLF2//ZHX1//IRX2//DR1//CUX2//NR6A1//SMAD5//MITF//DLL4//H2AFY2//PURB//RARB//SMO//TRPS1//UBE2I//NCBP2//UPF3A//EIF4E//ZC3H12A//DBR1//RBM7//SNRPF//NOVA2//RNPC3//SRSF11//NCOA1//RPL7L1//COPS7B//APEX1//WDR48//RAD52//EXOG//POP4//ATP7A//SUB1//EBF3//TAF5L//NFYC//NPM1//PBX3//YBX2//PLAGL1//RFX4//ALX4//HIF3A//TAF11//BTF3//XBP1//MIXL1//FUBP3//HAND2//CREB5//ALDOB//RPE//H6PD//COX4I1//ACPP//CMPK1//NPPC//PDE1B//WDHD1//CDK2AP1//USP51//TAOK3//ATRX//UBE2W//APEX2//KDM2A//TSN//FSTL3//PCGF3//USP16//ZNF273//FOXP4//ZNF488//ZNF597//ZNF558//ZNF320//E2F5//CRTC1//GABPB1//ALX3//ACAD8//ZNF544//HOXA9//ZNF699//AFF3//PAX7//COMMD8//PRKAA2//ARNTL2//RNF4//SP4//ZNF37A//ZNF75D//ZNF329//SAP30L//SLTM//NCOA4//PIAS2//ZNF251//CHURC1//REXO4//RORA//CSDE1//LZTR1//CKS2//CERS6//QRICH1//THAP10//ZNF704//SENP3//CCNC//CDK12//QKI//BAK1//G3BP2//NF2//MAPKBP1//EREG//RFK//TRIM71//DDX6//DDX3Y//WIZ//C14ORF39//MCCC2//F2R//FBXW11//CRLF3//PRAMEF19//TMEM100//CTDSPL2//CALCR//THBS1//NOD1//ASZ1//EXD1//C5AR1//PSMA8//PSMA1//PSMC6//CD40LG//TIMP2//TFRC//IL2//TRIAP1//YES1//FAM20C//GTPBP1//SULT6B1//RIPK3//PCK1//CUL2//N4BP2//AEN//PGBD5//NMD3//RAP2C// |
| GO:0051353 | positive regulation of oxidoreductase activity | Biological process | 6 | 47 | 393 | 17653 | 5.7342861783336 | 0.000581700424340571 | 0.357639997255935 | 3.23530061939107 | 0.0152671755725191 | RFK//NOD1//NUS1//DHFR//RIPK3//ATP7A// |
| GO:0002544 | chronic inflammatory response | Biological process | 4 | 19 | 393 | 17653 | 9.45654211865542 | 0.000719233737122833 | 0.396810829876492 | 3.14312994933011 | 0.0101781170483461 | CYP19A1//LTA//THBS1//VNN1// |
| GO:0090304 | nucleic acid metabolic process | Biological process | 148 | 5329 | 393 | 17653 | 1.24750405506 | 0.000812735898478974 | 0.396810829876492 | 3.09005055707133 | 0.376590330788804 | KPNA1//KAT7//MCM10//DHFR//GFI1//KLF2//ZHX1//IRX2//DR1//CUX2//NR6A1//SMAD5//MITF//DLL4//H2AFY2//PURB//RARB//SMO//TRPS1//UBE2I//NCBP2//UPF3A//EIF4E//ZC3H12A//DBR1//RBM7//SNRPF//NOVA2//RNPC3//SRSF11//NCOA1//RPL7L1//COPS7B//APEX1//WDR48//RAD52//EXOG//POP4//SUB1//EBF3//TAF5L//NFYC//NPM1//PBX3//YBX2//PLAGL1//RFX4//ALX4//HIF3A//TAF11//BTF3//XBP1//MIXL1//FUBP3//HAND2//CREB5//WDHD1//CDK2AP1//USP51//TAOK3//ATRX//UBE2W//APEX2//KDM2A//TSN//FSTL3//PCGF3//USP16//ZNF273//FOXP4//ZNF488//ZNF597//ZNF558//ZNF320//E2F5//CRTC1//GABPB1//ALX3//ACAD8//ZNF544//HOXA9//ZNF699//AFF3//PAX7//COMMD8//PRKAA2//ARNTL2//RNF4//SP4//ZNF37A//ZNF75D//ZNF329//SAP30L//SLTM//NCOA4//PIAS2//ZNF251//CHURC1//REXO4//RORA//CSDE1//LZTR1//CKS2//CERS6//QRICH1//THAP10//ZNF704//SENP3//CCNC//CDK12//QKI//G3BP2//NF2//MAPKBP1//EREG//TRIM71//DDX6//DDX3Y//WIZ//C14ORF39//F2R//FBXW11//CRLF3//PRAMEF19//TMEM100//CTDSPL2//NOD1//ASZ1//EXD1//C5AR1//PSMA8//PSMA1//PSMC6//CD40LG//TFRC//IL2//TRIAP1//YES1//NPPC//RIPK3//GTPBP1//PCK1//CUL2//N4BP2//AEN//PGBD5//NMD3//RAP2C// |
| GO:0097327 | response to antineoplastic agent | Biological process | 8 | 91 | 393 | 17653 | 3.94888571987809 | 0.000954908021171102 | 0.396810829876492 | 3.02003845859596 | 0.0203562340966921 | DHFR//EIF4E//GJB2//PCK1//KAT7//ATRX//ZC3H12A//KIF1B// |
| GO:0051341 | regulation of oxidoreductase activity | Biological process | 8 | 92 | 393 | 17653 | 3.90596304900985 | 0.00102596223714626 | 0.396810829876492 | 2.98886862411779 | 0.0203562340966921 | GFI1//RFK//NOD1//NUS1//DHFR//RIPK3//ATP7A//GZMK// |
| GO:0031098 | stress-activated protein kinase signaling cascade | Biological process | 16 | 296 | 393 | 17653 | 2.42803108451963 | 0.0010277328223922 | 0.396810829876492 | 2.98811977331932 | 0.0407124681933842 | NOD1//MUL1//CD40LG//FAS//TAOK3//SERPINB3//GRIK2//TRAF4//RASSF2//MAP3K8//FBXW11//ZC3H12A//HAND2//KAT7//GFRAL//STK24// |
| GO:1901360 | organic cyclic compound metabolic process | Biological process | 172 | 6394 | 393 | 17653 | 1.20831950436995 | 0.00110360876851201 | 0.396810829876492 | 2.95718485757555 | 0.43765903307888 | KPNA1//KAT7//MCM10//DHFR//GFI1//KLF2//ZHX1//IRX2//DR1//CUX2//NR6A1//SMAD5//MITF//DLL4//H2AFY2//PURB//RARB//SMO//TRPS1//UBE2I//NCBP2//UPF3A//EIF4E//ZC3H12A//DBR1//RBM7//SNRPF//NOVA2//RNPC3//SRSF11//NCOA1//RPL7L1//COPS7B//APEX1//WDR48//RAD52//EXOG//POP4//ATP7A//SUB1//EBF3//TAF5L//NFYC//NPM1//PBX3//YBX2//PLAGL1//RFX4//ALX4//HIF3A//TAF11//BTF3//XBP1//MIXL1//FUBP3//HAND2//CREB5//ALDOB//RPE//H6PD//COX4I1//ACPP//CMPK1//NPPC//PDE1B//WDHD1//CDK2AP1//USP51//TAOK3//ATRX//UBE2W//APEX2//KDM2A//TSN//FSTL3//PCGF3//USP16//ZNF273//FOXP4//ZNF488//ZNF597//ZNF558//ZNF320//E2F5//CRTC1//GABPB1//ALX3//ACAD8//ZNF544//HOXA9//ZNF699//AFF3//PAX7//COMMD8//PRKAA2//ARNTL2//RNF4//SP4//ZNF37A//ZNF75D//ZNF329//SAP30L//SLTM//NCOA4//PIAS2//ZNF251//CHURC1//REXO4//RORA//CSDE1//LZTR1//CKS2//CERS6//QRICH1//THAP10//ZNF704//SENP3//CCNC//CDK12//QKI//CPQ//CYP19A1//DHCR24//IDI2//MCCC2//RFK//CPOX//BAK1//G3BP2//NF2//MAPKBP1//EREG//TRIM71//DDX6//DDX3Y//WIZ//C14ORF39//EBPL//F2R//FBXW11//CRLF3//PRAMEF19//TMEM100//CTDSPL2//CALCR//THBS1//NOD1//ASZ1//EXD1//C5AR1//PSMA8//PSMA1//PSMC6//CD40LG//SCD//TIMP2//TFRC//IL2//TRIAP1//YES1//FAM20C//GTPBP1//SULT6B1//BCHE//RIPK3//PCK1//CUL2//N4BP2//AEN//PGBD5//NMD3//RAP2C// |
| GO:0006725 | cellular aromatic compound metabolic process | Biological process | 167 | 6178 | 393 | 17653 | 1.21421204849845 | 0.00110475744637056 | 0.396810829876492 | 2.95673306249095 | 0.424936386768448 | KPNA1//KAT7//MCM10//DHFR//GFI1//KLF2//ZHX1//IRX2//DR1//CUX2//NR6A1//SMAD5//MITF//DLL4//H2AFY2//PURB//RARB//SMO//TRPS1//UBE2I//NCBP2//UPF3A//EIF4E//ZC3H12A//DBR1//RBM7//SNRPF//NOVA2//RNPC3//SRSF11//NCOA1//RPL7L1//COPS7B//APEX1//WDR48//RAD52//EXOG//POP4//ATP7A//SUB1//EBF3//TAF5L//NFYC//NPM1//PBX3//YBX2//PLAGL1//RFX4//ALX4//HIF3A//TAF11//BTF3//XBP1//MIXL1//FUBP3//HAND2//CREB5//ALDOB//RPE//H6PD//COX4I1//ACPP//CMPK1//NPPC//PDE1B//WDHD1//CDK2AP1//USP51//TAOK3//ATRX//UBE2W//APEX2//KDM2A//TSN//FSTL3//PCGF3//USP16//ZNF273//FOXP4//ZNF488//ZNF597//ZNF558//ZNF320//E2F5//CRTC1//GABPB1//ALX3//ACAD8//ZNF544//HOXA9//ZNF699//AFF3//PAX7//COMMD8//PRKAA2//ARNTL2//RNF4//SP4//ZNF37A//ZNF75D//ZNF329//SAP30L//SLTM//NCOA4//PIAS2//ZNF251//CHURC1//REXO4//RORA//CSDE1//LZTR1//CKS2//CERS6//QRICH1//THAP10//ZNF704//SENP3//CCNC//CDK12//QKI//CPQ//CPOX//BAK1//G3BP2//NF2//MAPKBP1//EREG//RFK//TRIM71//DDX6//DDX3Y//WIZ//C14ORF39//MCCC2//F2R//FBXW11//CRLF3//PRAMEF19//TMEM100//CTDSPL2//CALCR//THBS1//NOD1//ASZ1//EXD1//C5AR1//PSMA8//PSMA1//PSMC6//CD40LG//TIMP2//TFRC//IL2//TRIAP1//YES1//FAM20C//GTPBP1//SULT6B1//BCHE//RIPK3//PCK1//CUL2//N4BP2//AEN//PGBD5//NMD3//RAP2C// |
| GO:0046483 | heterocycle metabolic process | Biological process | 166 | 6136 | 393 | 17653 | 1.21520265002604 | 0.00111480197658633 | 0.396810829876492 | 2.95280226993741 | 0.422391857506361 | KPNA1//KAT7//MCM10//DHFR//GFI1//KLF2//ZHX1//IRX2//DR1//CUX2//NR6A1//SMAD5//MITF//DLL4//H2AFY2//PURB//RARB//SMO//TRPS1//UBE2I//NCBP2//UPF3A//EIF4E//ZC3H12A//DBR1//RBM7//SNRPF//NOVA2//RNPC3//SRSF11//NCOA1//RPL7L1//COPS7B//APEX1//WDR48//RAD52//EXOG//POP4//ATP7A//SUB1//EBF3//TAF5L//NFYC//NPM1//PBX3//YBX2//PLAGL1//RFX4//ALX4//HIF3A//TAF11//BTF3//XBP1//MIXL1//FUBP3//HAND2//CREB5//ALDOB//RPE//H6PD//COX4I1//ACPP//CMPK1//NPPC//PDE1B//WDHD1//CDK2AP1//USP51//TAOK3//ATRX//UBE2W//APEX2//KDM2A//TSN//FSTL3//PCGF3//USP16//ZNF273//FOXP4//ZNF488//ZNF597//ZNF558//ZNF320//E2F5//CRTC1//GABPB1//ALX3//ACAD8//ZNF544//HOXA9//ZNF699//AFF3//PAX7//COMMD8//PRKAA2//ARNTL2//RNF4//SP4//ZNF37A//ZNF75D//ZNF329//SAP30L//SLTM//NCOA4//PIAS2//ZNF251//CHURC1//REXO4//RORA//CSDE1//LZTR1//CKS2//CERS6//QRICH1//THAP10//ZNF704//SENP3//CCNC//CDK12//QKI//MCCC2//RFK//CPOX//BAK1//G3BP2//NF2//MAPKBP1//EREG//TRIM71//DDX6//DDX3Y//WIZ//C14ORF39//F2R//FBXW11//CRLF3//PRAMEF19//TMEM100//CTDSPL2//CALCR//THBS1//NOD1//ASZ1//EXD1//C5AR1//PSMA8//PSMA1//PSMC6//CD40LG//TIMP2//TFRC//IL2//TRIAP1//YES1//FAM20C//GTPBP1//SULT6B1//BCHE//RIPK3//PCK1//CUL2//N4BP2//AEN//PGBD5//NMD3//RAP2C// |
| GO:0009595 | detection of biotic stimulus | Biological process | 4 | 22 | 393 | 17653 | 8.16701364792968 | 0.00128763606485016 | 0.421445799729914 | 2.89020686784689 | 0.0101781170483461 | NOD1//PGLYRP4//CD1D//SMO// |
| GO:1902680 | positive regulation of RNA biosynthetic process | Biological process | 50 | 1451 | 393 | 17653 | 1.54784889950425 | 0.00130864435817362 | 0.421445799729914 | 2.88317836282145 | 0.127226463104326 | NCOA1//XBP1//KLF2//USP16//CKS2//F2R//FBXW11//EBF3//SMAD5//MITF//NPM1//CRLF3//ARNTL2//RNF4//RORA//NCOA4//FUBP3//PIAS2//CHURC1//HAND2//CREB5//PSMC6//FSTL3//KAT7//E2F5//CRTC1//GABPB1//NR6A1//HOXA9//IL2//NFYC//PAX7//PBX3//TRIAP1//CDK12//PLAGL1//ATRX//RARB//RFX4//ALX4//HIF3A//SMO//TRPS1//YES1//ZC3H12A//MIXL1//CCNC//SUB1//PCK1//NMD3// |
| GO:0051252 | regulation of RNA metabolic process | Biological process | 114 | 3984 | 393 | 17653 | 1.28532067200098 | 0.00158228973512116 | 0.457548511527224 | 2.80071398933193 | 0.290076335877863 | DHFR//GFI1//KLF2//ZHX1//IRX2//DR1//CUX2//NR6A1//SMAD5//MITF//DLL4//H2AFY2//PURB//RARB//SMO//TRPS1//UBE2I//RBM7//NCOA1//PCGF3//KAT7//ZNF597//ZNF558//ZNF320//KDM2A//ACAD8//TAF5L//ZNF544//APEX1//ZNF699//AFF3//NFYC//YBX2//ATRX//COMMD8//PRKAA2//ARNTL2//REXO4//RORA//TAF11//BTF3//ZNF37A//ZNF75D//CSDE1//ZNF329//SAP30L//LZTR1//ZNF251//FSTL3//USP16//ZNF273//SUB1//WDHD1//FOXP4//CKS2//CERS6//GABPB1//ALX3//NPM1//QRICH1//THAP10//RFX4//ZNF704//HIF3A//SP4//SLTM//CREB5//XBP1//G3BP2//F2R//FBXW11//EBF3//CRLF3//RNF4//NCOA4//FUBP3//PIAS2//CHURC1//HAND2//ZNF488//EREG//PRAMEF19//TMEM100//CTDSPL2//NCBP2//NOD1//ZC3H12A//CRTC1//CDK12//PSMA8//PSMA1//PSMC6//E2F5//HOXA9//IL2//PAX7//PBX3//TRIAP1//PLAGL1//ALX4//YES1//MIXL1//CCNC//C14ORF39//RIPK3//CD40LG//GTPBP1//TRIM71//QKI//PCK1//CUL2//NMD3//RAP2C//NOVA2// |
| GO:0006357 | regulation of transcription by RNA polymerase II | Biological process | 70 | 2227 | 393 | 17653 | 1.41189953051321 | 0.0016872392908338 | 0.457548511527224 | 2.77282331971142 | 0.178117048346056 | KLF2//ZHX1//IRX2//DR1//CUX2//NR6A1//GFI1//SMAD5//MITF//DLL4//H2AFY2//PURB//RARB//SMO//TRPS1//UBE2I//NCOA1//XBP1//TMEM100//FSTL3//CTDSPL2//PSMC6//USP16//KAT7//E2F5//CRTC1//EBF3//GABPB1//HOXA9//IL2//NFYC//NPM1//PAX7//PBX3//CRLF3//TRIAP1//CDK12//PLAGL1//ATRX//ARNTL2//RFX4//RNF4//ALX4//RORA//HIF3A//YES1//ZC3H12A//MIXL1//CCNC//FUBP3//PIAS2//HAND2//SUB1//PCK1//PSMA8//PSMA1//CUL2//ZNF273//WDHD1//FOXP4//CKS2//CERS6//ALX3//QRICH1//THAP10//ZNF704//SP4//ZNF37A//SLTM//CREB5// |
| GO:1901615 | organic hydroxy compound metabolic process | Biological process | 22 | 497 | 393 | 17653 | 1.98834738712171 | 0.00182606722552783 | 0.457548511527224 | 2.73848323822649 | 0.0559796437659033 | ALDH1B1//MOGAT3//ATP7A//CPQ//DHCR24//PRKAA2//IDI2//DHFR//GFI1//CYP19A1//EBPL//NUS1//PDE1B//UBE2I//RPE65//LRAT//INPP5B//SYNJ1//NFYC//SCD//PCK1//HAND2// |
| GO:0035107 | appendage morphogenesis | Biological process | 10 | 149 | 393 | 17653 | 3.01466946735659 | 0.00187697309075499 | 0.457548511527224 | 2.72654195360156 | 0.0254452926208651 | ASPH//BAK1//ALX3//HOXA9//ALX4//AFF3//RARB//ATRX//HAND2//SEMA3C// |
| GO:0035108 | limb morphogenesis | Biological process | 10 | 149 | 393 | 17653 | 3.01466946735659 | 0.00187697309075499 | 0.457548511527224 | 2.72654195360156 | 0.0254452926208651 | ALX3//HOXA9//ALX4//AFF3//RARB//ATRX//HAND2//SEMA3C//ASPH//BAK1// |
| GO:0048266 | behavioral response to pain | Biological process | 3 | 12 | 393 | 17653 | 11.2296437659033 | 0.00207427003230274 | 0.457548511527224 | 2.68313470700906 | 0.00763358778625954 | PIRT//VWA1//THBS1// |
| GO:0061158 | 3'-UTR-mediated mRNA destabilization | Biological process | 3 | 12 | 393 | 17653 | 11.2296437659033 | 0.00207427003230274 | 0.457548511527224 | 2.68313470700906 | 0.00763358778625954 | TRIM71//ZC3H12A//QKI// |
| GO:0006826 | iron ion transport | Biological process | 6 | 60 | 393 | 17653 | 4.49185750636132 | 0.00212885864282066 | 0.457548511527224 | 2.67185317495487 | 0.0152671755725191 | B2M//TFRC//ATP6V0E1//ATP7A//STEAP2//SFXN1// |
| GO:0045893 | positive regulation of transcription, DNA-templated | Biological process | 49 | 1450 | 393 | 17653 | 1.51793805387383 | 0.00218730155301768 | 0.457548511527224 | 2.66009133868913 | 0.124681933842239 | NCOA1//XBP1//KLF2//PSMC6//FSTL3//USP16//KAT7//E2F5//CRTC1//EBF3//GABPB1//NR6A1//HOXA9//IL2//MITF//NFYC//NPM1//PAX7//PBX3//CRLF3//TRIAP1//CDK12//PLAGL1//ATRX//ARNTL2//RARB//RFX4//RNF4//ALX4//RORA//HIF3A//SMO//TRPS1//YES1//ZC3H12A//MIXL1//CCNC//FUBP3//PIAS2//HAND2//SUB1//PCK1//SMAD5//CKS2//F2R//FBXW11//NCOA4//CHURC1//CREB5// |
| GO:1903508 | positive regulation of nucleic acid-templated transcription | Biological process | 49 | 1450 | 393 | 17653 | 1.51793805387383 | 0.00218730155301768 | 0.457548511527224 | 2.66009133868913 | 0.124681933842239 | NCOA1//XBP1//KLF2//USP16//CKS2//F2R//FBXW11//EBF3//SMAD5//MITF//NPM1//CRLF3//ARNTL2//RNF4//RORA//NCOA4//FUBP3//PIAS2//CHURC1//HAND2//CREB5//PSMC6//FSTL3//KAT7//E2F5//CRTC1//GABPB1//NR6A1//HOXA9//IL2//NFYC//PAX7//PBX3//TRIAP1//CDK12//PLAGL1//ATRX//RARB//RFX4//ALX4//HIF3A//SMO//TRPS1//YES1//ZC3H12A//MIXL1//CCNC//SUB1//PCK1// |
| GO:2001141 | regulation of RNA biosynthetic process | Biological process | 108 | 3774 | 393 | 17653 | 1.28542822121628 | 0.00220632870951057 | 0.457548511527224 | 2.65632978378533 | 0.274809160305344 | DHFR//GFI1//KLF2//ZHX1//IRX2//DR1//CUX2//NR6A1//SMAD5//MITF//DLL4//H2AFY2//PURB//RARB//SMO//TRPS1//UBE2I//NCOA1//PCGF3//KAT7//ZNF597//ZNF558//ZNF320//KDM2A//ACAD8//TAF5L//ZNF544//APEX1//ZNF699//AFF3//NFYC//YBX2//ATRX//COMMD8//PRKAA2//ARNTL2//REXO4//RORA//TAF11//BTF3//ZNF37A//ZNF75D//CSDE1//ZNF329//SAP30L//LZTR1//ZNF251//FSTL3//USP16//ZNF273//SUB1//WDHD1//FOXP4//CKS2//CERS6//GABPB1//ALX3//NPM1//QRICH1//THAP10//RFX4//ZNF704//HIF3A//SP4//SLTM//CREB5//XBP1//G3BP2//F2R//FBXW11//EBF3//CRLF3//RNF4//NCOA4//FUBP3//PIAS2//CHURC1//HAND2//ZNF488//EREG//PRAMEF19//TMEM100//CTDSPL2//NOD1//ZC3H12A//CRTC1//PSMC6//E2F5//HOXA9//IL2//PAX7//PBX3//TRIAP1//CDK12//PLAGL1//ALX4//YES1//MIXL1//CCNC//C14ORF39//RIPK3//CD40LG//PCK1//PSMA8//PSMA1//CUL2//NMD3//RAP2C// |
| GO:0032774 | RNA biosynthetic process | Biological process | 112 | 3940 | 393 | 17653 | 1.27687319977784 | 0.00223260400419908 | 0.457548511527224 | 2.65118830068234 | 0.28498727735369 | DHFR//GFI1//KLF2//ZHX1//IRX2//DR1//CUX2//NR6A1//SMAD5//MITF//DLL4//H2AFY2//PURB//RARB//SMO//TRPS1//UBE2I//NCOA1//SUB1//NCBP2//EBF3//TAF5L//NFYC//NPM1//PBX3//YBX2//PLAGL1//RFX4//ALX4//HIF3A//TAF11//BTF3//XBP1//MIXL1//FUBP3//HAND2//CREB5//SNRPF//SRSF11//FSTL3//PCGF3//USP16//ZNF273//KAT7//FOXP4//ZNF488//ZNF597//ZNF558//ZNF320//E2F5//KDM2A//CRTC1//GABPB1//ALX3//ACAD8//ZNF544//HOXA9//APEX1//ZNF699//AFF3//PAX7//ATRX//COMMD8//PRKAA2//ARNTL2//RNF4//SP4//ZNF37A//ZNF75D//ZNF329//SAP30L//SLTM//ZC3H12A//NCOA4//PIAS2//ZNF251//CHURC1//REXO4//RORA//CSDE1//LZTR1//WDHD1//CKS2//CERS6//QRICH1//THAP10//ZNF704//CCNC//CDK12//G3BP2//EREG//F2R//FBXW11//CRLF3//PRAMEF19//TMEM100//CTDSPL2//NOD1//C5AR1//PSMC6//IL2//TRIAP1//YES1//C14ORF39//RIPK3//CD40LG//PCK1//PSMA8//PSMA1//CUL2//NMD3//RAP2C// |
| GO:0034641 | cellular nitrogen compound metabolic process | Biological process | 180 | 6851 | 393 | 17653 | 1.18016983089336 | 0.00253304500909391 | 0.464934459118104 | 2.59635709326217 | 0.458015267175573 | KPNA1//KAT7//MCM10//DHFR//GFI1//KLF2//ZHX1//IRX2//DR1//CUX2//NR6A1//SMAD5//MITF//DLL4//H2AFY2//PURB//RARB//SMO//TRPS1//UBE2I//NCBP2//UPF3A//EIF4E//ZC3H12A//DBR1//RBM7//SNRPF//NOVA2//RNPC3//SRSF11//NCOA1//RPL7L1//COPS7B//APEX1//WDR48//RAD52//EXOG//POP4//EIF3A//ATP7A//SUB1//EBF3//TAF5L//NFYC//NPM1//PBX3//YBX2//PLAGL1//RFX4//ALX4//HIF3A//TAF11//BTF3//XBP1//MIXL1//FUBP3//HAND2//CREB5//ALDOB//RPE//H6PD//COX4I1//ACPP//CMPK1//NPPC//PDE1B//WDHD1//CDK2AP1//USP51//TAOK3//ATRX//UBE2W//APEX2//KDM2A//TSN//FSTL3//PCGF3//USP16//ZNF273//FOXP4//ZNF488//ZNF597//ZNF558//ZNF320//E2F5//CRTC1//GABPB1//ALX3//ACAD8//ZNF544//HOXA9//ZNF699//AFF3//PAX7//COMMD8//PRKAA2//ARNTL2//RNF4//SP4//ZNF37A//ZNF75D//ZNF329//SAP30L//SLTM//NCOA4//PIAS2//ZNF251//CHURC1//REXO4//RORA//CSDE1//LZTR1//CKS2//CERS6//QRICH1//THAP10//ZNF704//SENP3//CCNC//CDK12//QKI//MRPS7//COA1//EIF1AX//EIF5//DDX3Y//GTPBP1//DDX6//OGFOD1//SEC11A//PSMA8//PSMA1//PSMC6//GCLM//MCCC2//RFK//CPOX//BAK1//G3BP2//NF2//MAPKBP1//EREG//TRIM71//WIZ//C14ORF39//XPNPEP1//VNN1//LARP4B//THBS1//F2R//FBXW11//CRLF3//PRAMEF19//MEX3B//CRAT//TMEM100//CTDSPL2//CALCR//NOD1//SATL1//ASZ1//EXD1//C5AR1//CPQ//CD40LG//TIMP2//TFRC//IL2//TRIAP1//YES1//FAM20C//SULT6B1//RIPK3//PCK1//CUL2//N4BP2//AEN//PGBD5//NMD3//RAP2C// |
| GO:0048534 | hematopoietic or lymphoid organ development | Biological process | 33 | 882 | 393 | 17653 | 1.6806269581624 | 0.00254848144907692 | 0.464934459118104 | 2.59371852338304 | 0.083969465648855 | FSTL3//AGPAT5//MIXL1//CMTM7//BAK1//RIPK3//LTA//GFI1//CD40LG//IL2//DLL4//SMAD5//SFXN1//WDR1//PDE1B//MITF//TFRC//B2M//VNN1//FAM20C//ATP7A//KLF2//CD1D//XBP1//PURB//HOXA9//THBS1//RASSF2//HAND2//RORA//PSMA8//PSMA1//PSMC6// |
| GO:0016045 | detection of bacterium | Biological process | 3 | 13 | 393 | 17653 | 10.36582501468 | 0.00265230397789858 | 0.464934459118104 | 2.57637670335981 | 0.00763358778625954 | NOD1//PGLYRP4//CD1D// |
| GO:0060081 | membrane hyperpolarization | Biological process | 3 | 13 | 393 | 17653 | 10.36582501468 | 0.00265230397789858 | 0.464934459118104 | 2.57637670335981 | 0.00763358778625954 | TRPC5//CRTC1//KCNQ3// |
| GO:0097659 | nucleic acid-templated transcription | Biological process | 111 | 3924 | 393 | 17653 | 1.27063247504105 | 0.00278488602269258 | 0.464934459118104 | 2.55519257454002 | 0.282442748091603 | DHFR//GFI1//KLF2//ZHX1//IRX2//DR1//CUX2//NR6A1//SMAD5//MITF//DLL4//H2AFY2//PURB//RARB//SMO//TRPS1//UBE2I//NCOA1//SUB1//NCBP2//EBF3//TAF5L//NFYC//NPM1//PBX3//YBX2//PLAGL1//RFX4//ALX4//HIF3A//TAF11//BTF3//XBP1//MIXL1//FUBP3//HAND2//CREB5//SNRPF//SRSF11//FSTL3//PCGF3//USP16//ZNF273//KAT7//FOXP4//ZNF488//ZNF597//ZNF558//ZNF320//E2F5//KDM2A//CRTC1//GABPB1//ALX3//ACAD8//ZNF544//HOXA9//APEX1//ZNF699//AFF3//PAX7//ATRX//COMMD8//PRKAA2//ARNTL2//RNF4//SP4//ZNF37A//ZNF75D//ZNF329//SAP30L//SLTM//ZC3H12A//NCOA4//PIAS2//ZNF251//CHURC1//REXO4//RORA//CSDE1//LZTR1//WDHD1//CKS2//CERS6//QRICH1//THAP10//ZNF704//CCNC//CDK12//G3BP2//EREG//F2R//FBXW11//CRLF3//PRAMEF19//TMEM100//CTDSPL2//NOD1//C5AR1//PSMC6//IL2//TRIAP1//YES1//C14ORF39//RIPK3//CD40LG//PCK1//PSMA8//PSMA1//CUL2//RAP2C// |
| GO:0048522 | positive regulation of cellular process | Biological process | 140 | 5142 | 393 | 17653 | 1.22298726349783 | 0.00284857980958019 | 0.464934459118104 | 2.54537160826813 | 0.356234096692112 | STK24//MAP3K8//F2R//TAOK3//NOD1//THBS1//C5AR1//NCOA1//FAS//CDK2AP1//STX1A//SMO//EREG//MUL1//NPM1//TRIAP1//PLAGL1//BAK1//XBP1//TRAF4//CD40LG//IL2//RARB//SERPINB3//PRAMEF19//TRPC5//RNF152//PRKAA2//ZC3H12A//TRPC3//ATRX//WIZ//ATP7A//LMAN1//RIPK3//CALCR//DYNLT1//ZNF804A//LARP4B//UPF3A//KLF2//USP16//CKS2//FBXW11//EBF3//SMAD5//MITF//CRLF3//ARNTL2//RNF4//RORA//NCOA4//FUBP3//PIAS2//CHURC1//HAND2//CREB5//SEMA3C//CD274//MCAM//NPPC//WDR1//TFRC//PAX7//HLA-DPB1//YES1//CD3G//FKBP1A//NCBP2//RAP2C//RASSF2//OSBPL8//VNN1//ARPC5//F2RL2//OXCT1//VSNL1//CD209//CD1D//MOB1B//GZMK//LTA//CANT1//ZDHHC17//UBE2I//TIMP2//GRIK2//NUS1//NF2//TMEM100//H2AFY2//FAM20C//CCNC//TSPAN5//DLL4//ASPH//PSMC6//EIF4E//FSTL3//KAT7//E2F5//CRTC1//GABPB1//NR6A1//HOXA9//NFYC//PBX3//CDK12//RFX4//ALX4//HIF3A//TRPS1//MIXL1//B2M//ZNF488//WDR48//CUX2//DCUN1D5//PGLYRP4//ACPP//SUB1//SNAP47//SLC24A2//GTPBP1//SLC30A2//TRIM71//QKI//PCK1//GFI1//PSMA8//PSMA1//APEX1//PPP1R13B//YWHAG//TUBB2B//NMD3//CEP135//NBPF3//SYNJ1//CYP19A1// |
| GO:0031328 | positive regulation of cellular biosynthetic process | Biological process | 58 | 1812 | 393 | 17653 | 1.43779103404501 | 0.00294548596537275 | 0.464934459118104 | 2.53084304224583 | 0.147582697201018 | NCOA1//XBP1//ATRX//WIZ//LARP4B//NPM1//UPF3A//THBS1//KLF2//USP16//CKS2//F2R//FBXW11//EBF3//SMAD5//MITF//CRLF3//ARNTL2//RNF4//RORA//NCOA4//FUBP3//PIAS2//CHURC1//HAND2//CREB5//NPPC//EREG//TIMP2//PRKAA2//PSMC6//FSTL3//KAT7//E2F5//CRTC1//GABPB1//NR6A1//HOXA9//IL2//NFYC//PAX7//PBX3//TRIAP1//CDK12//PLAGL1//RARB//RFX4//ALX4//HIF3A//SMO//TRPS1//YES1//ZC3H12A//MIXL1//CCNC//SUB1//PCK1//NMD3// |
| GO:0010557 | positive regulation of macromolecule biosynthetic process | Biological process | 55 | 1698 | 393 | 17653 | 1.45495973409819 | 0.00295582547683179 | 0.464934459118104 | 2.52932121191675 | 0.139949109414758 | NCOA1//XBP1//ATRX//WIZ//LARP4B//NPM1//UPF3A//THBS1//KLF2//USP16//CKS2//F2R//FBXW11//EBF3//SMAD5//MITF//CRLF3//ARNTL2//RNF4//RORA//NCOA4//FUBP3//PIAS2//CHURC1//HAND2//CREB5//EREG//PSMC6//FSTL3//KAT7//E2F5//CRTC1//GABPB1//NR6A1//HOXA9//IL2//NFYC//PAX7//PBX3//TRIAP1//CDK12//PLAGL1//RARB//RFX4//ALX4//HIF3A//SMO//TRPS1//YES1//ZC3H12A//MIXL1//CCNC//SUB1//PCK1//NMD3// |
| GO:1903506 | regulation of nucleic acid-templated transcription | Biological process | 107 | 3766 | 393 | 17653 | 1.27623142108513 | 0.00295945285078437 | 0.464934459118104 | 2.52878857470118 | 0.272264631043257 | DHFR//GFI1//KLF2//ZHX1//IRX2//DR1//CUX2//NR6A1//SMAD5//MITF//DLL4//H2AFY2//PURB//RARB//SMO//TRPS1//UBE2I//NCOA1//PCGF3//KAT7//ZNF597//ZNF558//ZNF320//KDM2A//ACAD8//TAF5L//ZNF544//APEX1//ZNF699//AFF3//NFYC//YBX2//ATRX//COMMD8//PRKAA2//ARNTL2//REXO4//RORA//TAF11//BTF3//ZNF37A//ZNF75D//CSDE1//ZNF329//SAP30L//LZTR1//ZNF251//FSTL3//USP16//ZNF273//SUB1//WDHD1//FOXP4//CKS2//CERS6//GABPB1//ALX3//NPM1//QRICH1//THAP10//RFX4//ZNF704//HIF3A//SP4//SLTM//CREB5//XBP1//G3BP2//F2R//FBXW11//EBF3//CRLF3//RNF4//NCOA4//FUBP3//PIAS2//CHURC1//HAND2//ZNF488//EREG//PRAMEF19//TMEM100//CTDSPL2//NOD1//ZC3H12A//CRTC1//PSMC6//E2F5//HOXA9//IL2//PAX7//PBX3//TRIAP1//CDK12//PLAGL1//ALX4//YES1//MIXL1//CCNC//C14ORF39//RIPK3//CD40LG//PCK1//PSMA8//PSMA1//CUL2//RAP2C// |
| GO:0050870 | positive regulation of T cell activation | Biological process | 12 | 213 | 393 | 17653 | 2.53062394724582 | 0.00302663602271402 | 0.464934459118104 | 2.51903980330218 | 0.0305343511450382 | MAP3K8//CD274//HLA-DPB1//YES1//CD3G//CD40LG//VNN1//CD209//IL2//TFRC//CD1D//XBP1// |
| GO:0048002 | antigen processing and presentation of peptide antigen | Biological process | 11 | 187 | 393 | 17653 | 2.64226912138901 | 0.00320409326674874 | 0.464934459118104 | 2.49429485068868 | 0.0279898218829517 | B2M//PSMA8//PSMA1//PSMC6//IFI30//CLTA//DYNC1LI2//HLA-DPB1//CTSF//CLEC4M//CD209// |
| GO:0097286 | iron ion import | Biological process | 3 | 14 | 393 | 17653 | 9.62540894220284 | 0.0033203317675289 | 0.464934459118104 | 2.47881851942598 | 0.00763358778625954 | B2M//STEAP2//TFRC// |
| GO:0097329 | response to antimetabolite | Biological process | 3 | 14 | 393 | 17653 | 9.62540894220284 | 0.0033203317675289 | 0.464934459118104 | 2.47881851942598 | 0.00763358778625954 | DHFR//KAT7//ATRX// |
| GO:0098543 | detection of other organism | Biological process | 3 | 14 | 393 | 17653 | 9.62540894220284 | 0.0033203317675289 | 0.464934459118104 | 2.47881851942598 | 0.00763358778625954 | NOD1//PGLYRP4//CD1D// |
| GO:0009893 | positive regulation of metabolic process | Biological process | 96 | 3329 | 393 | 17653 | 1.29533890240519 | 0.00333827982077514 | 0.464934459118104 | 2.47647726276605 | 0.244274809160305 | STK24//MAP3K8//F2R//TAOK3//NOD1//THBS1//C5AR1//NCOA1//FAS//CDK2AP1//EREG//MUL1//BAK1//XBP1//TRAF4//CD40LG//RNF152//PRKAA2//ZC3H12A//ATRX//WIZ//KIF1B//CUX2//MITF//DLL4//SMO//CALCR//FUBP3//ZNF804A//QKI//HAND2//RIPK3//TRPC5//SERPINB3//LARP4B//NPM1//UPF3A//KLF2//USP16//CKS2//FBXW11//EBF3//SMAD5//CRLF3//ARNTL2//RNF4//RORA//NCOA4//PIAS2//CHURC1//CREB5//NPPC//PAX7//FKBP1A//NCBP2//RAP2C//RASSF2//OSBPL8//RFK//MOB1B//IL2//TIMP2//CCNC//TFRC//ASPH//PSMC6//FSTL3//KAT7//E2F5//CRTC1//GABPB1//NR6A1//HOXA9//NFYC//PBX3//TRIAP1//CDK12//PLAGL1//RARB//RFX4//ALX4//HIF3A//TRPS1//YES1//MIXL1//NUS1//DHFR//ATP7A//DCUN1D5//SUB1//GTPBP1//TRIM71//PCK1//NMD3//WDR48//UBE2I// |
| GO:0006351 | transcription, DNA-templated | Biological process | 110 | 3907 | 393 | 17653 | 1.26466425825376 | 0.00343085578756475 | 0.464934459118104 | 2.46459753667456 | 0.279898218829517 | DHFR//GFI1//KLF2//ZHX1//IRX2//DR1//CUX2//NR6A1//SMAD5//MITF//DLL4//H2AFY2//PURB//RARB//SMO//TRPS1//UBE2I//NCOA1//SUB1//NCBP2//EBF3//TAF5L//NFYC//NPM1//PBX3//YBX2//PLAGL1//RFX4//ALX4//HIF3A//TAF11//BTF3//XBP1//MIXL1//FUBP3//HAND2//CREB5//SNRPF//SRSF11//PCGF3//KAT7//ZNF597//ZNF558//ZNF320//KDM2A//ACAD8//ZNF544//APEX1//ZNF699//AFF3//ATRX//COMMD8//PRKAA2//ARNTL2//REXO4//RORA//ZNF37A//ZNF75D//CSDE1//ZNF329//SAP30L//LZTR1//ZNF251//FSTL3//USP16//ZNF273//WDHD1//FOXP4//CKS2//CERS6//GABPB1//ALX3//QRICH1//THAP10//ZNF704//SP4//SLTM//CCNC//CDK12//G3BP2//EREG//F2R//FBXW11//CRLF3//RNF4//NCOA4//PIAS2//CHURC1//ZNF488//PRAMEF19//TMEM100//CTDSPL2//NOD1//ZC3H12A//CRTC1//C5AR1//PSMC6//E2F5//HOXA9//IL2//PAX7//TRIAP1//YES1//C14ORF39//RIPK3//CD40LG//PCK1//PSMA8//PSMA1//CUL2// |
| GO:0006355 | regulation of transcription, DNA-templated | Biological process | 106 | 3745 | 393 | 17653 | 1.27139357990467 | 0.00352589209967592 | 0.464934459118104 | 2.45273098223305 | 0.269720101781171 | DHFR//GFI1//KLF2//ZHX1//IRX2//DR1//CUX2//NR6A1//SMAD5//MITF//DLL4//H2AFY2//PURB//RARB//SMO//TRPS1//UBE2I//NCOA1//FSTL3//USP16//ZNF273//SUB1//WDHD1//FOXP4//CKS2//CERS6//GABPB1//ALX3//NFYC//NPM1//QRICH1//THAP10//ARNTL2//RFX4//ZNF704//HIF3A//SP4//ZNF37A//SLTM//CREB5//XBP1//G3BP2//F2R//FBXW11//EBF3//CRLF3//RNF4//RORA//NCOA4//FUBP3//PIAS2//CHURC1//HAND2//ZNF488//EREG//PRAMEF19//TMEM100//CTDSPL2//NOD1//ZC3H12A//CRTC1//PSMC6//KAT7//E2F5//HOXA9//IL2//PAX7//PBX3//TRIAP1//CDK12//PLAGL1//ATRX//ALX4//YES1//MIXL1//CCNC//C14ORF39//RIPK3//CD40LG//PCK1//PSMA8//PSMA1//CUL2//PCGF3//ZNF597//ZNF558//ZNF320//KDM2A//ACAD8//TAF5L//ZNF544//APEX1//ZNF699//AFF3//YBX2//COMMD8//PRKAA2//REXO4//TAF11//BTF3//ZNF75D//CSDE1//ZNF329//SAP30L//LZTR1//ZNF251// |
| GO:0006766 | vitamin metabolic process | Biological process | 9 | 137 | 393 | 17653 | 2.95085529614977 | 0.0035995177790305 | 0.464934459118104 | 2.44375567699207 | 0.0229007633587786 | MCCC2//RFK//ACPP//RPE65//LRAT//GFI1//VNN1//UBE2I//DHFR// |
| GO:0035116 | embryonic hindlimb morphogenesis | Biological process | 4 | 29 | 393 | 17653 | 6.19566552601562 | 0.00369842311579431 | 0.464934459118104 | 2.43198340514467 | 0.0101781170483461 | ALX3//AFF3//RARB//ALX4// |
| GO:2000112 | regulation of cellular macromolecule biosynthetic process | Biological process | 121 | 4376 | 393 | 17653 | 1.24203555363282 | 0.00376096194795402 | 0.464934459118104 | 2.42470106058909 | 0.307888040712468 | DHFR//GFI1//KLF2//ZHX1//IRX2//DR1//CUX2//NR6A1//SMAD5//MITF//DLL4//H2AFY2//PURB//RARB//SMO//TRPS1//UBE2I//NCOA1//PCGF3//KAT7//ZNF597//ZNF558//ZNF320//KDM2A//ACAD8//TAF5L//ZNF544//APEX1//ZNF699//AFF3//NFYC//YBX2//ATRX//COMMD8//PRKAA2//ARNTL2//REXO4//RORA//TAF11//BTF3//ZNF37A//ZNF75D//CSDE1//ZNF329//SAP30L//LZTR1//ZNF251//FSTL3//USP16//ZNF273//SUB1//WDHD1//FOXP4//CKS2//CERS6//GABPB1//ALX3//NPM1//QRICH1//THAP10//RFX4//ZNF704//HIF3A//SP4//SLTM//CREB5//DDX6//EIF4E//EIF5//NCBP2//OGFOD1//XBP1//G3BP2//NF2//TRIM71//WIZ//LARP4B//UPF3A//THBS1//F2R//FBXW11//EBF3//CRLF3//RNF4//NCOA4//FUBP3//PIAS2//CHURC1//HAND2//ZNF488//EREG//PRAMEF19//MEX3B//TMEM100//CTDSPL2//NOD1//ZC3H12A//CRTC1//NECAB3//NECAB1//PSMC6//E2F5//HOXA9//IL2//PAX7//PBX3//TRIAP1//CDK12//PLAGL1//ALX4//YES1//MIXL1//CCNC//C14ORF39//RIPK3//CD40LG//QKI//PCK1//PSMA8//PSMA1//CUL2// |
| GO:0051726 | regulation of cell cycle | Biological process | 41 | 1197 | 393 | 17653 | 1.53856439232092 | 0.00397761900846092 | 0.464934459118104 | 2.40037681760352 | 0.104325699745547 | NPM1//TRIAP1//PLAGL1//DHCR24//HEPACAM//PRKAA2//THBS1//CUL2//TAOK3//CDC14A//CKS2//MAP3K8//STK24//YWHAG//CEP135//USP51//ATRX//WIZ//RAB6C//PSMA8//PSMA1//PSMC6//CCNC//PPP1R13B//EREG//BTG3//TIMP2//EIF4E//MAP9//NPPC//CRLF3//RNF4//APEX1//CCSAP//TPRA1//USP16//E2F5//NF2//CDK12//BAK1//CDKL1// |
| GO:0010556 | regulation of macromolecule biosynthetic process | Biological process | 123 | 4467 | 393 | 17653 | 1.23684457864885 | 0.00398277656864757 | 0.464934459118104 | 2.39981405656461 | 0.312977099236641 | DHFR//GFI1//KLF2//ZHX1//IRX2//DR1//CUX2//NR6A1//SMAD5//MITF//DLL4//H2AFY2//PURB//RARB//SMO//TRPS1//UBE2I//NCOA1//PCGF3//KAT7//ZNF597//ZNF558//ZNF320//KDM2A//ACAD8//TAF5L//ZNF544//APEX1//ZNF699//AFF3//NFYC//YBX2//ATRX//COMMD8//PRKAA2//ARNTL2//REXO4//RORA//TAF11//BTF3//ZNF37A//ZNF75D//CSDE1//ZNF329//SAP30L//LZTR1//ZNF251//FSTL3//USP16//ZNF273//SUB1//WDHD1//FOXP4//CKS2//CERS6//GABPB1//ALX3//NPM1//QRICH1//THAP10//RFX4//ZNF704//HIF3A//SP4//SLTM//CREB5//DDX6//EIF4E//EIF5//NCBP2//OGFOD1//XBP1//G3BP2//NF2//TRIM71//WIZ//LARP4B//UPF3A//THBS1//F2R//FBXW11//EBF3//CRLF3//RNF4//NCOA4//FUBP3//PIAS2//CHURC1//HAND2//ZNF488//EREG//PRAMEF19//MEX3B//TMEM100//CTDSPL2//NOD1//ZC3H12A//CRTC1//NECAB3//NECAB1//PSMC6//E2F5//HOXA9//IL2//PAX7//PBX3//TRIAP1//CDK12//PLAGL1//ALX4//YES1//MIXL1//CCNC//C14ORF39//RIPK3//CD40LG//QKI//PCK1//PSMA8//PSMA1//CUL2//NMD3//RAP2C// |
| GO:0033555 | multicellular organismal response to stress | Biological process | 6 | 68 | 393 | 17653 | 3.96340368208352 | 0.00401268123712989 | 0.464934459118104 | 2.39656533875956 | 0.0152671755725191 | EIF4E//GRIK2//VDAC1//PIRT//VWA1//THBS1// |
| GO:0010628 | positive regulation of gene expression | Biological process | 59 | 1877 | 393 | 17653 | 1.41193176811571 | 0.0040340726950232 | 0.464934459118104 | 2.39425627995494 | 0.150127226463104 | NCOA1//XBP1//LARP4B//NPM1//UPF3A//THBS1//KLF2//USP16//CKS2//F2R//FBXW11//EBF3//SMAD5//MITF//CRLF3//ARNTL2//RNF4//RORA//NCOA4//FUBP3//PIAS2//CHURC1//HAND2//CREB5//NCBP2//PSMC6//FSTL3//KAT7//E2F5//CRTC1//GABPB1//NR6A1//HOXA9//IL2//NFYC//PAX7//PBX3//TRIAP1//CDK12//PLAGL1//ATRX//RARB//RFX4//ALX4//HIF3A//SMO//TRPS1//YES1//ZC3H12A//MIXL1//CCNC//SUB1//PCK1//KIF1B//CUX2//DLL4//CALCR//ZNF804A//QKI// |
| GO:0016082 | synaptic vesicle priming | Biological process | 3 | 15 | 393 | 17653 | 8.98371501272265 | 0.00408246566970905 | 0.464934459118104 | 2.38907745864729 | 0.00763358778625954 | STX1A//SNAP47//SYNJ1// |
| GO:0046165 | alcohol biosynthetic process | Biological process | 9 | 140 | 393 | 17653 | 2.88762268266085 | 0.00415294874090475 | 0.464934459118104 | 2.38164342927529 | 0.0229007633587786 | DHCR24//PRKAA2//IDI2//DHFR//GFI1//NUS1//NFYC//SCD//PCK1// |
| GO:0009891 | positive regulation of biosynthetic process | Biological process | 58 | 1841 | 393 | 17653 | 1.41514250607798 | 0.00415473469349332 | 0.464934459118104 | 2.38145670348771 | 0.147582697201018 | NCOA1//XBP1//ATRX//WIZ//LARP4B//NPM1//UPF3A//THBS1//KLF2//USP16//CKS2//F2R//FBXW11//EBF3//SMAD5//MITF//CRLF3//ARNTL2//RNF4//RORA//NCOA4//FUBP3//PIAS2//CHURC1//HAND2//CREB5//NPPC//EREG//TIMP2//PRKAA2//PSMC6//FSTL3//KAT7//E2F5//CRTC1//GABPB1//NR6A1//HOXA9//IL2//NFYC//PAX7//PBX3//TRIAP1//CDK12//PLAGL1//RARB//RFX4//ALX4//HIF3A//SMO//TRPS1//YES1//ZC3H12A//MIXL1//CCNC//SUB1//PCK1//NMD3// |
| GO:0021696 | cerebellar cortex morphogenesis | Biological process | 4 | 30 | 393 | 17653 | 5.9891433418151 | 0.00419355345352718 | 0.464934459118104 | 2.3774178168354 | 0.0101781170483461 | ATP7A//RORA//RFX4//SMO// |
| GO:0030097 | hemopoiesis | Biological process | 31 | 842 | 393 | 17653 | 1.65377176600001 | 0.00431405064318731 | 0.467128756747493 | 2.36511476096558 | 0.0788804071246819 | FSTL3//AGPAT5//MIXL1//CMTM7//BAK1//CD40LG//IL2//DLL4//SMAD5//SFXN1//WDR1//PDE1B//MITF//TFRC//RIPK3//B2M//VNN1//FAM20C//ATP7A//KLF2//CD1D//XBP1//PURB//HOXA9//THBS1//RASSF2//RORA//PSMA8//PSMA1//PSMC6//GFI1// |
| GO:0055076 | transition metal ion homeostasis | Biological process | 9 | 141 | 393 | 17653 | 2.8671430891668 | 0.00435148775322964 | 0.467128756747493 | 2.36136223441266 | 0.0229007633587786 | ATP7A//TMEM199//TFRC//NCOA4//SLC30A5//STEAP2//B2M//SFXN1//SLC30A2// |
| GO:1903039 | positive regulation of leukocyte cell-cell adhesion | Biological process | 12 | 224 | 393 | 17653 | 2.40635223555071 | 0.00453174995621912 | 0.478878514904842 | 2.34373406077467 | 0.0305343511450382 | MAP3K8//CD274//HLA-DPB1//YES1//CD3G//CD40LG//VNN1//CD209//IL2//TFRC//CD1D//XBP1// |
| GO:0048771 | tissue remodeling | Biological process | 10 | 169 | 393 | 17653 | 2.65790384991794 | 0.00465250327358357 | 0.484075071373011 | 2.33231331261981 | 0.0254452926208651 | SEMA3C//ATP7A//DLL4//BAK1//F2R//HAND2//IL2//TFRC//MITF//RASSF2// |
| GO:0071347 | cellular response to interleukin-1 | Biological process | 10 | 170 | 393 | 17653 | 2.64226912138901 | 0.00484828581947821 | 0.491551403928977 | 2.31441178526024 | 0.0254452926208651 | NOD1//MAP3K8//PSMA8//FBXW11//PSMA1//PSMC6//KLF2//PCK1//RORA//ZC3H12A// |
| GO:0051173 | positive regulation of nitrogen compound metabolic process | Biological process | 86 | 2965 | 393 | 17653 | 1.30286592090075 | 0.00489393014974876 | 0.491551403928977 | 2.31034223355292 | 0.21882951653944 | STK24//MAP3K8//F2R//TAOK3//NOD1//THBS1//C5AR1//NCOA1//FAS//CDK2AP1//EREG//MUL1//BAK1//XBP1//TRAF4//CD40LG//ATRX//WIZ//TRPC5//SERPINB3//LARP4B//NPM1//UPF3A//KLF2//USP16//CKS2//FBXW11//EBF3//SMAD5//MITF//CRLF3//ARNTL2//RNF4//RORA//NCOA4//FUBP3//PIAS2//CHURC1//HAND2//CREB5//CALCR//NPPC//PAX7//FKBP1A//NCBP2//RAP2C//RASSF2//RIPK3//OSBPL8//IL2//TIMP2//CCNC//PRKAA2//TFRC//ASPH//PSMC6//FSTL3//KAT7//E2F5//CRTC1//GABPB1//NR6A1//HOXA9//NFYC//PBX3//TRIAP1//CDK12//PLAGL1//RARB//RFX4//ALX4//HIF3A//SMO//TRPS1//YES1//ZC3H12A//MIXL1//DCUN1D5//SUB1//GTPBP1//TRIM71//QKI//PCK1//NMD3//WDR48//UBE2I// |
| GO:0021534 | cell proliferation in hindbrain | Biological process | 3 | 16 | 393 | 17653 | 8.42223282442748 | 0.00494240654549319 | 0.491551403928977 | 2.30606153388285 | 0.00763358778625954 | RORA//C5AR1// |
| GO:0022409 | positive regulation of cell-cell adhesion | Biological process | 13 | 257 | 393 | 17653 | 2.27214582033841 | 0.00517187640016421 | 0.50691884194653 | 2.28635186263311 | 0.0330788804071247 | MAP3K8//CD274//HLA-DPB1//YES1//CD3G//CD40LG//VNN1//CD209//IL2//TFRC//CD1D//XBP1//FSTL3// |
| GO:0031638 | zymogen activation | Biological process | 5 | 51 | 393 | 17653 | 4.40378186898169 | 0.00540561402471869 | 0.511413370880363 | 2.26715496731639 | 0.0127226463104326 | THBS1//DHCR24//ST14//ASPH//BAK1// |
| GO:0002520 | immune system development | Biological process | 33 | 928 | 393 | 17653 | 1.5973200184259 | 0.00550809710213759 | 0.511413370880363 | 2.25899841217858 | 0.083969465648855 | FSTL3//AGPAT5//MIXL1//CMTM7//BAK1//RIPK3//LTA//GFI1//CD40LG//IL2//DLL4//SMAD5//SFXN1//WDR1//PDE1B//MITF//TFRC//B2M//VNN1//FAM20C//ATP7A//KLF2//CD1D//XBP1//PURB//HOXA9//THBS1//RASSF2//HAND2//RORA//PSMA8//PSMA1//PSMC6// |
| GO:0042102 | positive regulation of T cell proliferation | Biological process | 7 | 96 | 393 | 17653 | 3.27531276505513 | 0.00561344986356051 | 0.511413370880363 | 2.25077015189522 | 0.0178117048346056 | IL2//CD274//CD209//HLA-DPB1//TFRC//CD1D//CD40LG// |
| GO:0042098 | T cell proliferation | Biological process | 10 | 174 | 393 | 17653 | 2.58152730250651 | 0.00569699451250578 | 0.511413370880363 | 2.24435419886693 | 0.0254452926208651 | CD274//CD209//HLA-DPB1//IL2//TFRC//CD1D//CD40LG//RIPK3//BTN2A2//FKBP1A// |
| GO:0017085 | response to insecticide | Biological process | 3 | 17 | 393 | 17653 | 7.92680736416704 | 0.00590346408100857 | 0.511413370880363 | 2.22889307483421 | 0.00763358778625954 | ZC3H12A//KIF1B//CPOX// |
| GO:0042753 | positive regulation of circadian rhythm | Biological process | 3 | 17 | 393 | 17653 | 7.92680736416704 | 0.00590346408100857 | 0.511413370880363 | 2.22889307483421 | 0.00763358778625954 | FBXW11//ARNTL2//RORA// |
| GO:0051023 | regulation of immunoglobulin secretion | Biological process | 3 | 17 | 393 | 17653 | 7.92680736416704 | 0.00590346408100857 | 0.511413370880363 | 2.22889307483421 | 0.00763358778625954 | IL2//XBP1//CD40LG// |
| GO:0010039 | response to iron ion | Biological process | 4 | 33 | 393 | 17653 | 5.44467576528645 | 0.00594261461224353 | 0.511413370880363 | 2.22602243349799 | 0.0101781170483461 | ATP7A//B2M//CPOX//FKBP1A// |
| GO:0048736 | appendage development | Biological process | 10 | 176 | 393 | 17653 | 2.55219176497802 | 0.00616270534743916 | 0.511413370880363 | 2.21022859635181 | 0.0254452926208651 | ASPH//BAK1//ALX3//HOXA9//ALX4//AFF3//RARB//ATRX//HAND2//SEMA3C// |
| GO:0060173 | limb development | Biological process | 10 | 176 | 393 | 17653 | 2.55219176497802 | 0.00616270534743916 | 0.511413370880363 | 2.21022859635181 | 0.0254452926208651 | ASPH//BAK1//ALX3//HOXA9//ALX4//AFF3//RARB//ATRX//HAND2//SEMA3C// |
| GO:0042129 | regulation of T cell proliferation | Biological process | 9 | 149 | 393 | 17653 | 2.71320252062093 | 0.00621883071768436 | 0.511413370880363 | 2.20629126492248 | 0.0229007633587786 | CD274//CD209//HLA-DPB1//IL2//TFRC//CD1D//CD40LG//RIPK3//BTN2A2// |
| GO:0016070 | RNA metabolic process | Biological process | 130 | 4821 | 393 | 17653 | 1.21124554205968 | 0.00623407268822654 | 0.511413370880363 | 2.20522813822991 | 0.330788804071247 | DHFR//GFI1//KLF2//ZHX1//IRX2//DR1//CUX2//NR6A1//SMAD5//MITF//DLL4//H2AFY2//PURB//RARB//SMO//TRPS1//UBE2I//NCBP2//UPF3A//EIF4E//ZC3H12A//DBR1//RBM7//SNRPF//NOVA2//RNPC3//SRSF11//NCOA1//RPL7L1//POP4//SUB1//EBF3//TAF5L//NFYC//NPM1//PBX3//YBX2//PLAGL1//RFX4//ALX4//HIF3A//TAF11//BTF3//XBP1//MIXL1//FUBP3//HAND2//CREB5//FSTL3//PCGF3//USP16//ZNF273//KAT7//FOXP4//ZNF488//ZNF597//ZNF558//ZNF320//E2F5//KDM2A//CRTC1//GABPB1//ALX3//ACAD8//ZNF544//HOXA9//APEX1//ZNF699//AFF3//PAX7//ATRX//COMMD8//PRKAA2//ARNTL2//RNF4//SP4//ZNF37A//ZNF75D//ZNF329//SAP30L//SLTM//NCOA4//PIAS2//ZNF251//CHURC1//REXO4//RORA//CSDE1//LZTR1//WDHD1//CKS2//CERS6//QRICH1//THAP10//ZNF704//SENP3//CCNC//CDK12//QKI//G3BP2//MAPKBP1//EREG//TRIM71//DDX6//DDX3Y//F2R//FBXW11//CRLF3//PRAMEF19//TSN//TMEM100//CTDSPL2//NOD1//ASZ1//EXD1//C5AR1//PSMA8//PSMA1//PSMC6//IL2//TRIAP1//YES1//C14ORF39//RIPK3//CD40LG//GTPBP1//PCK1//CUL2//NMD3//RAP2C// |
| GO:0006066 | alcohol metabolic process | Biological process | 15 | 324 | 393 | 17653 | 2.07956366035246 | 0.00625024173363472 | 0.511413370880363 | 2.20410318560737 | 0.0381679389312977 | ALDH1B1//MOGAT3//DHCR24//PRKAA2//IDI2//DHFR//GFI1//NUS1//RPE65//LRAT//INPP5B//SYNJ1//NFYC//SCD//PCK1// |
| GO:0031325 | positive regulation of cellular metabolic process | Biological process | 88 | 3074 | 393 | 17653 | 1.28589284502211 | 0.00627640245202871 | 0.511413370880363 | 2.20228921660016 | 0.223918575063613 | STK24//MAP3K8//F2R//TAOK3//NOD1//THBS1//C5AR1//NCOA1//FAS//CDK2AP1//EREG//MUL1//BAK1//XBP1//TRAF4//CD40LG//RNF152//PRKAA2//ZC3H12A//ATRX//WIZ//RIPK3//TRPC5//SERPINB3//LARP4B//NPM1//UPF3A//KLF2//USP16//CKS2//FBXW11//EBF3//SMAD5//MITF//CRLF3//ARNTL2//RNF4//RORA//NCOA4//FUBP3//PIAS2//CHURC1//HAND2//CREB5//CALCR//NPPC//PAX7//FKBP1A//NCBP2//RAP2C//RASSF2//OSBPL8//MOB1B//IL2//TIMP2//CCNC//TFRC//ASPH//PSMC6//FSTL3//KAT7//E2F5//CRTC1//GABPB1//NR6A1//HOXA9//NFYC//PBX3//TRIAP1//CDK12//PLAGL1//RARB//RFX4//ALX4//HIF3A//SMO//TRPS1//YES1//MIXL1//DCUN1D5//SUB1//GTPBP1//TRIM71//QKI//PCK1//NMD3//WDR48//UBE2I// |
| GO:0010468 | regulation of gene expression | Biological process | 131 | 4873 | 393 | 17653 | 1.20753813530337 | 0.0066850429481023 | 0.538225541166855 | 2.17489579826528 | 0.333333333333333 | DHFR//GFI1//KLF2//ZHX1//IRX2//DR1//CUX2//NR6A1//SMAD5//MITF//DLL4//H2AFY2//PURB//RARB//SMO//TRPS1//UBE2I//NCBP2//UPF3A//EIF4E//ZC3H12A//RBM7//NCOA1//PCGF3//KAT7//ZNF597//ZNF558//ZNF320//KDM2A//ACAD8//TAF5L//ZNF544//APEX1//ZNF699//AFF3//NFYC//YBX2//ATRX//COMMD8//PRKAA2//ARNTL2//REXO4//RORA//TAF11//BTF3//ZNF37A//ZNF75D//CSDE1//ZNF329//SAP30L//LZTR1//ZNF251//FSTL3//USP16//ZNF273//SUB1//WDHD1//FOXP4//CKS2//CERS6//GABPB1//ALX3//NPM1//QRICH1//THAP10//RFX4//ZNF704//HIF3A//SP4//SLTM//CREB5//DDX6//EIF5//OGFOD1//XBP1//G3BP2//RPE65//TRIM71//KIF1B//CALCR//FUBP3//ZNF804A//QKI//HAND2//BAK1//THBS1//LARP4B//F2R//FBXW11//EBF3//CRLF3//RNF4//NCOA4//PIAS2//CHURC1//ZNF488//EREG//PRAMEF19//MEX3B//TSN//C5AR1//TMEM100//CTDSPL2//ASZ1//EXD1//NOD1//CRTC1//CDK12//PSMA8//PSMA1//PSMC6//E2F5//HOXA9//IL2//PAX7//PBX3//TRIAP1//PLAGL1//ALX4//YES1//MIXL1//CCNC//C14ORF39//RIPK3//CD40LG//GTPBP1//PCK1//CUL2//INPP5B//ATP7A//DDX3Y// |
| GO:0060255 | regulation of macromolecule metabolic process | Biological process | 167 | 6425 | 393 | 17653 | 1.16753339075851 | 0.00680977129664926 | 0.541817450343988 | 2.16686747344273 | 0.424936386768448 | KPNA1//DHFR//GFI1//KLF2//ZHX1//IRX2//DR1//CUX2//NR6A1//SMAD5//MITF//DLL4//H2AFY2//PURB//RARB//SMO//TRPS1//UBE2I//NCBP2//UPF3A//STK24//MAP3K8//F2R//TAOK3//NOD1//THBS1//C5AR1//DUSP16//EIF4E//ZC3H12A//RBM7//NCOA1//FKBP1A//IL2//FAS//CDK2AP1//EREG//PCGF3//KAT7//ZNF597//ZNF558//ZNF320//KDM2A//ACAD8//TAF5L//ZNF544//APEX1//ZNF699//AFF3//NFYC//YBX2//ATRX//COMMD8//PRKAA2//ARNTL2//REXO4//RORA//TAF11//BTF3//ZNF37A//ZNF75D//CSDE1//ZNF329//SAP30L//LZTR1//ZNF251//FSTL3//USP16//ZNF273//SUB1//WDHD1//FOXP4//CKS2//CERS6//GABPB1//ALX3//NPM1//QRICH1//THAP10//RFX4//ZNF704//HIF3A//SP4//SLTM//CREB5//DDX6//EIF5//OGFOD1//NF2//YWHAG//MUL1//BAK1//XBP1//TRAF4//G3BP2//CD40LG//RPE65//TRIM71//SERPINB3//SERPINB4//ATP7A//DDX3Y//USP51//WIZ//KIF1B//CALCR//FUBP3//ZNF804A//QKI//HAND2//TRPC5//TIMP2//LARP4B//FBXW11//EBF3//CRLF3//RNF4//NCOA4//PIAS2//CHURC1//ZNF488//PRAMEF19//MEX3B//TSN//TMEM100//CTDSPL2//ASZ1//EXD1//PAX7//MOB1B//RAP2C//RASSF2//RIPK3//OSBPL8//MTM1//CRTC1//NECAB3//NECAB1//DHCR24//TRIAP1//CDK12//PSMA8//PSMA1//PSMC6//CCNC//TFRC//ASPH//E2F5//HOXA9//PBX3//PLAGL1//ALX4//YES1//MIXL1//GRIK2//NPPC//C14ORF39//NOVA2//DCUN1D5//GTPBP1//PCK1//CUL2//EIF3A//INPP5B//WDR48//NMD3//RAD52// |
| GO:0051403 | stress-activated MAPK cascade | Biological process | 13 | 267 | 393 | 17653 | 2.18704672594371 | 0.00705731437559543 | 0.55419399662226 | 2.15136053602409 | 0.0330788804071247 | NOD1//MUL1//CD40LG//FAS//TAOK3//SERPINB3//GRIK2//TRAF4//RASSF2//ZC3H12A//HAND2//MAP3K8//FBXW11// |
| GO:0038061 | NIK/NF-kappaB signaling | Biological process | 8 | 126 | 393 | 17653 | 2.85197301991195 | 0.00723099836940814 | 0.55419399662226 | 2.14080173639962 | 0.0203562340966921 | TRAF4//RASSF2//NOD1//RIPK3//PSMA8//FBXW11//PSMA1//PSMC6// |
| GO:0031295 | T cell costimulation | Biological process | 6 | 77 | 393 | 17653 | 3.50014870625558 | 0.00736113665124664 | 0.55419399662226 | 2.13305512000352 | 0.0152671755725191 | MAP3K8//CD274//HLA-DPB1//YES1//CD3G//CD40LG// |
| GO:0070887 | cellular response to chemical stimulus | Biological process | 90 | 3175 | 393 | 17653 | 1.27328244274809 | 0.00741284230336929 | 0.55419399662226 | 2.13001523871611 | 0.229007633587786 | NCOA1//CCR5//NAT2//MARC2//RORA//BAK1//XBP1//FKBP1A//SMAD5//RPE65//ATP6V0E1//TRIM71//NCBP2//STK24//RARB//THBS1//C5AR1//CYP19A1//EREG//IL2//STX1A//ATP7A//PRDX2//TSN//TMEM100//FSTL3//CTDSPL2//NR6A1//RNF4//NCOA4//PIAS2//PIP5K1C//PCK1//YWHAG//UFM1//PSMA8//FAS//LTA//PSMA1//PSMC6//CD40LG//GJB2//GPX3//RAD52//PYROXD1//ZC3H12A//LTBP1//PRKAA2//TFRC//GCLM//OXCT1//VSNL1//DLL4//PDE1B//YES1//TMEM199//NUS1//FAM20C//OSBPL8//BCHE//PIP5K1A//IFI30//HLA-DPB1//B2M//MUL1//NPM1//HIF3A//CUL2//GFI1//KLF2//APEX1//NOD1//MAP3K8//FBXW11//UBE2W//CD274//SLC41A1//CUX2//SYP//PTPN7//SMO//EIF4E//ATRX//VNN1//DYNC1LI2//KIF1B//LAPTM5//ADAM23//LRAT//DHFR// |
| GO:0019222 | regulation of metabolic process | Biological process | 178 | 6920 | 393 | 17653 | 1.15541999441086 | 0.007455051596336 | 0.55419399662226 | 2.12754934645291 | 0.452926208651399 | KPNA1//DHFR//GFI1//KLF2//ZHX1//IRX2//DR1//CUX2//NR6A1//SMAD5//MITF//DLL4//H2AFY2//PURB//RARB//SMO//TRPS1//UBE2I//NCBP2//UPF3A//STK24//MAP3K8//F2R//TAOK3//NOD1//THBS1//C5AR1//DUSP16//EIF4E//ZC3H12A//RBM7//NCOA1//FKBP1A//IL2//FAS//CDK2AP1//ATP7A//EREG//PCGF3//KAT7//ZNF597//ZNF558//ZNF320//KDM2A//ACAD8//TAF5L//ZNF544//APEX1//ZNF699//AFF3//NFYC//YBX2//ATRX//COMMD8//PRKAA2//ARNTL2//REXO4//RORA//TAF11//BTF3//ZNF37A//ZNF75D//CSDE1//ZNF329//SAP30L//LZTR1//ZNF251//FSTL3//USP16//ZNF273//SUB1//WDHD1//FOXP4//CKS2//CERS6//GABPB1//ALX3//NPM1//QRICH1//THAP10//RFX4//ZNF704//HIF3A//SP4//SLTM//CREB5//DDX6//EIF5//OGFOD1//NF2//YWHAG//PSMA8//PSMA1//PSMC6//MUL1//BAK1//XBP1//TRAF4//G3BP2//CD40LG//RPE65//TRIM71//SERPINB3//SERPINB4//DDX3Y//RNF152//USP51//WIZ//KIF1B//CALCR//FUBP3//ZNF804A//QKI//HAND2//RIPK3//VDAC1//TRPC5//TIMP2//LARP4B//EXOC8//ATP6V0E1//FBXW11//EBF3//CRLF3//RNF4//NCOA4//PIAS2//CHURC1//ZNF488//PRAMEF19//MEX3B//TSN//TMEM100//CTDSPL2//NPPC//ASZ1//EXD1//PAX7//BTN2A2//MOB1B//RAP2C//RASSF2//OSBPL8//MTM1//CRTC1//RFK//PDE1B//NECAB3//NECAB1//DHCR24//TRIAP1//CDK12//SCD//CCNC//TFRC//ASPH//E2F5//HOXA9//PBX3//PLAGL1//ALX4//YES1//MIXL1//GRIK2//NUS1//C14ORF39//FAM20C//NOVA2//GZMK//DCUN1D5//GTPBP1//PCK1//CUL2//EIF3A//INPP5B//WDR48//NMD3//RAD52// |
| GO:0019884 | antigen processing and presentation of exogenous antigen | Biological process | 10 | 181 | 393 | 17653 | 2.48168923003388 | 0.00745699448360574 | 0.55419399662226 | 2.12743617818338 | 0.0254452926208651 | PSMA8//B2M//PSMA1//PSMC6//IFI30//CLTA//DYNC1LI2//HLA-DPB1//CTSF//CD1D// |
| GO:0031326 | regulation of cellular biosynthetic process | Biological process | 125 | 4640 | 393 | 17653 | 1.21009092304993 | 0.0078067422256652 | 0.569328096416853 | 2.10753016056351 | 0.318066157760814 | DHFR//GFI1//KLF2//ZHX1//IRX2//DR1//CUX2//NR6A1//SMAD5//MITF//DLL4//H2AFY2//PURB//RARB//SMO//TRPS1//UBE2I//NCOA1//PCGF3//KAT7//ZNF597//ZNF558//ZNF320//KDM2A//ACAD8//TAF5L//ZNF544//APEX1//ZNF699//AFF3//NFYC//YBX2//ATRX//COMMD8//PRKAA2//ARNTL2//REXO4//RORA//TAF11//BTF3//ZNF37A//ZNF75D//CSDE1//ZNF329//SAP30L//LZTR1//ZNF251//FSTL3//USP16//ZNF273//SUB1//WDHD1//FOXP4//CKS2//CERS6//GABPB1//ALX3//NPM1//QRICH1//THAP10//RFX4//ZNF704//HIF3A//SP4//SLTM//CREB5//DDX6//EIF4E//EIF5//NCBP2//OGFOD1//XBP1//G3BP2//NF2//TRIM71//WIZ//LARP4B//UPF3A//THBS1//F2R//FBXW11//EBF3//CRLF3//RNF4//NCOA4//FUBP3//PIAS2//CHURC1//HAND2//ZNF488//EREG//PRAMEF19//MEX3B//TMEM100//CTDSPL2//NPPC//NOD1//ZC3H12A//CRTC1//NECAB3//NECAB1//TIMP2//PSMC6//E2F5//HOXA9//IL2//PAX7//PBX3//TRIAP1//CDK12//PLAGL1//ALX4//YES1//MIXL1//CCNC//C14ORF39//RIPK3//CD40LG//QKI//PCK1//PSMA8//PSMA1//CUL2//NMD3//RAP2C// |
| GO:0031294 | lymphocyte costimulation | Biological process | 6 | 78 | 393 | 17653 | 3.45527500489333 | 0.00782899792499887 | 0.569328096416853 | 2.10629382204088 | 0.0152671755725191 | MAP3K8//CD274//HLA-DPB1//YES1//CD3G//CD40LG// |
| GO:0048518 | positive regulation of biological process | Biological process | 153 | 5841 | 393 | 17653 | 1.17660366114241 | 0.00797292972353052 | 0.573222475683741 | 2.09838206392839 | 0.389312977099237 | STK24//MAP3K8//F2R//TAOK3//NOD1//THBS1//C5AR1//NCOA1//EREG//B2M//FAS//CDK2AP1//STX1A//SMO//PGLYRP4//PSMA8//FBXW11//CD209//PSMA1//PSMC6//XBP1//ZC3H12A//LTA//MUL1//NPM1//TRIAP1//PLAGL1//BAK1//TRAF4//CD40LG//IL2//RARB//SERPINB3//PRAMEF19//TRPC5//RNF152//PRKAA2//TRPC3//ATRX//WIZ//RORA//ATP7A//HAND2//KIF1B//CUX2//MITF//DLL4//CALCR//FUBP3//ZNF804A//QKI//LMAN1//RIPK3//EDIL3//DYNLT1//LARP4B//UPF3A//CLTCL1//KLF2//USP16//CKS2//EBF3//SMAD5//CRLF3//ARNTL2//RNF4//NCOA4//PIAS2//CHURC1//CREB5//FSTL3//BTN2A2//BTN2A1//HLA-DPB1//CD3G//SEMA3C//CD274//MCAM//FAM20C//NPPC//WDR1//TFRC//PAX7//YES1//FKBP1A//NCBP2//RAP2C//RASSF2//OSBPL8//LRAT//CRTC1//VNN1//RFK//GFI1//ARPC5//F2RL2//OXCT1//VSNL1//CD1D//MOB1B//GZMK//CANT1//ZDHHC17//UBE2I//TIMP2//GRIK2//NUS1//TAF11//NF2//TMEM100//H2AFY2//CCNC//TSPAN5//ASPH//EIF4E//KAT7//E2F5//GABPB1//NR6A1//HOXA9//NFYC//PBX3//CDK12//RFX4//ALX4//HIF3A//TRPS1//MIXL1//CTDSPL2//MTM1//ZNF488//WDR48//DHFR//DCUN1D5//ACPP//SUB1//SNAP47//SLC24A2//GTPBP1//SLC30A2//TRIM71//PCK1//APEX1//PPP1R13B//YWHAG//FGF14//TUBB2B//NMD3//CEP135//NBPF3//SYNJ1//CYP19A1//PIRT// |
| GO:0042110 | T cell activation | Biological process | 19 | 463 | 393 | 17653 | 1.84331085574223 | 0.00805206789737622 | 0.573222475683741 | 2.09409257167068 | 0.0483460559796438 | IL2//DLL4//MAP3K8//CD274//HLA-DPB1//YES1//CD3G//CD40LG//RIPK3//B2M//VNN1//ATP7A//FKBP1A//CD209//TFRC//CD1D//XBP1//BTN2A2//RORA// |
| GO:0098581 | detection of external biotic stimulus | Biological process | 3 | 19 | 393 | 17653 | 7.09240658899156 | 0.00814032795856169 | 0.573469145664091 | 2.08935809784735 | 0.00763358778625954 | NOD1//PGLYRP4//CD1D// |
| GO:0010604 | positive regulation of macromolecule metabolic process | Biological process | 88 | 3106 | 393 | 17653 | 1.27264475389503 | 0.00826098924287348 | 0.575969796387148 | 2.08296794335797 | 0.223918575063613 | STK24//MAP3K8//F2R//TAOK3//NOD1//THBS1//C5AR1//NCOA1//FAS//CDK2AP1//EREG//MUL1//BAK1//XBP1//TRAF4//CD40LG//ATRX//WIZ//KIF1B//CUX2//MITF//DLL4//SMO//CALCR//ZC3H12A//FUBP3//ZNF804A//QKI//HAND2//TRPC5//SERPINB3//LARP4B//NPM1//UPF3A//KLF2//USP16//CKS2//FBXW11//EBF3//SMAD5//CRLF3//ARNTL2//RNF4//RORA//NCOA4//PIAS2//CHURC1//CREB5//PAX7//FKBP1A//NCBP2//RAP2C//RASSF2//RIPK3//OSBPL8//IL2//TIMP2//CCNC//TFRC//ASPH//PSMC6//FSTL3//KAT7//E2F5//CRTC1//GABPB1//NR6A1//HOXA9//NFYC//PBX3//TRIAP1//CDK12//PLAGL1//RARB//RFX4//ALX4//HIF3A//TRPS1//YES1//MIXL1//DCUN1D5//SUB1//GTPBP1//TRIM71//PCK1//NMD3//WDR48//UBE2I// |
| GO:0050801 | ion homeostasis | Biological process | 28 | 779 | 393 | 17653 | 1.61453158123385 | 0.00889888688363938 | 0.605099563672611 | 2.05066431363893 | 0.0712468193384224 | SLC24A2//GRIK2//ATP7A//TMEM199//TFRC//NCOA4//SLC30A5//ATP6V0E1//CCR5//F2R//IL2//TRPC5//C5AR1//BAK1//TRPC3//SLC41A1//FKBP1A//SLC9C2//SLC9A2//F2RL2//GCLM//STEAP2//B2M//SFXN1//PRKAA2//XBP1//SLC30A2//CALCR// |
| GO:0021587 | cerebellum morphogenesis | Biological process | 4 | 37 | 393 | 17653 | 4.85606216903927 | 0.00894720632371154 | 0.605099563672611 | 2.04831254765544 | 0.0101781170483461 | RFX4//SMO//ATP7A//RORA// |
| GO:0035137 | hindlimb morphogenesis | Biological process | 4 | 37 | 393 | 17653 | 4.85606216903927 | 0.00894720632371154 | 0.605099563672611 | 2.04831254765544 | 0.0101781170483461 | ALX3//AFF3//RARB//ALX4// |
| GO:0032872 | regulation of stress-activated MAPK cascade | Biological process | 11 | 216 | 393 | 17653 | 2.28752002638771 | 0.00925543166568177 | 0.612634751561454 | 2.0336033213031 | 0.0279898218829517 | MUL1//CD40LG//TAOK3//SERPINB3//GRIK2//NOD1//TRAF4//RASSF2//ZC3H12A//HAND2//FAS// |
| GO:0009889 | regulation of biosynthetic process | Biological process | 126 | 4707 | 393 | 17653 | 1.2024092751254 | 0.00930862416782616 | 0.612634751561454 | 2.03111450381736 | 0.320610687022901 | DHFR//GFI1//KLF2//ZHX1//IRX2//DR1//CUX2//NR6A1//SMAD5//MITF//DLL4//H2AFY2//PURB//RARB//SMO//TRPS1//UBE2I//NCOA1//PCGF3//KAT7//ZNF597//ZNF558//ZNF320//KDM2A//ACAD8//TAF5L//ZNF544//APEX1//ZNF699//AFF3//NFYC//YBX2//ATRX//COMMD8//PRKAA2//ARNTL2//REXO4//RORA//TAF11//BTF3//ZNF37A//ZNF75D//CSDE1//ZNF329//SAP30L//LZTR1//ZNF251//FSTL3//USP16//ZNF273//SUB1//WDHD1//FOXP4//CKS2//CERS6//GABPB1//ALX3//NPM1//QRICH1//THAP10//RFX4//ZNF704//HIF3A//SP4//SLTM//CREB5//DDX6//EIF4E//EIF5//NCBP2//OGFOD1//XBP1//G3BP2//NF2//TRIM71//WIZ//LARP4B//UPF3A//THBS1//F2R//FBXW11//EBF3//CRLF3//RNF4//NCOA4//FUBP3//PIAS2//CHURC1//HAND2//ZNF488//EREG//PRAMEF19//MEX3B//TMEM100//CTDSPL2//NPPC//NOD1//ZC3H12A//CRTC1//NECAB3//NECAB1//SCD//TIMP2//PSMC6//E2F5//HOXA9//IL2//PAX7//PBX3//TRIAP1//CDK12//PLAGL1//ALX4//YES1//MIXL1//CCNC//C14ORF39//RIPK3//CD40LG//QKI//PCK1//PSMA8//PSMA1//CUL2//NMD3//RAP2C// |
| GO:0048305 | immunoglobulin secretion | Biological process | 3 | 20 | 393 | 17653 | 6.73778625954198 | 0.00942096912056649 | 0.612634751561454 | 2.02590441970545 | 0.00763358778625954 | CD40LG//IL2//XBP1// |
| GO:0051000 | positive regulation of nitric-oxide synthase activity | Biological process | 3 | 20 | 393 | 17653 | 6.73778625954198 | 0.00942096912056649 | 0.612634751561454 | 2.02590441970545 | 0.00763358778625954 | NOD1//NUS1//DHFR// |
| GO:0070302 | regulation of stress-activated protein kinase signaling cascade | Biological process | 11 | 217 | 393 | 17653 | 2.27697845944583 | 0.00956179548533288 | 0.615870693974345 | 2.01946054954854 | 0.0279898218829517 | MUL1//CD40LG//FAS//TAOK3//SERPINB3//GRIK2//NOD1//TRAF4//RASSF2//ZC3H12A//HAND2// |
| GO:0019048 | modulation by virus of host morphology or physiology | Biological process | 4 | 38 | 393 | 17653 | 4.72827105932771 | 0.00982786255583046 | 0.627036174198881 | 2.00754092582462 | 0.0101781170483461 | KPNA1//ZC3H12A//CLEC4M//CD209// |
| GO:0015682 | ferric iron transport | Biological process | 4 | 39 | 393 | 17653 | 4.60703333985777 | 0.0107628887131968 | 0.661721966975909 | 1.96807115024696 | 0.0101781170483461 | B2M//TFRC//ATP6V0E1//STEAP2// |
| GO:0046006 | regulation of activated T cell proliferation | Biological process | 4 | 39 | 393 | 17653 | 4.60703333985777 | 0.0107628887131968 | 0.661721966975909 | 1.96807115024696 | 0.0101781170483461 | IL2//BTN2A2//CD274//RIPK3// |
| GO:0061014 | positive regulation of mRNA catabolic process | Biological process | 4 | 39 | 393 | 17653 | 4.60703333985777 | 0.0107628887131968 | 0.661721966975909 | 1.96807115024696 | 0.0101781170483461 | TRIM71//ZC3H12A//QKI//GTPBP1// |
| GO:0072512 | trivalent inorganic cation transport | Biological process | 4 | 39 | 393 | 17653 | 4.60703333985777 | 0.0107628887131968 | 0.661721966975909 | 1.96807115024696 | 0.0101781170483461 | B2M//TFRC//ATP6V0E1//STEAP2// |
| GO:0031323 | regulation of cellular metabolic process | Biological process | 166 | 6454 | 393 | 17653 | 1.15532746522464 | 0.0108981980083493 | 0.661929139382732 | 1.96264530570405 | 0.422391857506361 | KPNA1//DHFR//GFI1//KLF2//ZHX1//IRX2//DR1//CUX2//NR6A1//SMAD5//MITF//DLL4//H2AFY2//PURB//RARB//SMO//TRPS1//UBE2I//STK24//MAP3K8//F2R//TAOK3//NOD1//THBS1//C5AR1//DUSP16//RBM7//NCOA1//FKBP1A//IL2//ZC3H12A//FAS//CDK2AP1//ATP7A//EREG//PCGF3//KAT7//ZNF597//ZNF558//ZNF320//KDM2A//ACAD8//TAF5L//ZNF544//APEX1//ZNF699//AFF3//NFYC//YBX2//ATRX//COMMD8//PRKAA2//ARNTL2//REXO4//RORA//TAF11//BTF3//ZNF37A//ZNF75D//CSDE1//ZNF329//SAP30L//LZTR1//ZNF251//FSTL3//USP16//ZNF273//SUB1//WDHD1//FOXP4//CKS2//CERS6//GABPB1//ALX3//NPM1//QRICH1//THAP10//RFX4//ZNF704//HIF3A//SP4//SLTM//CREB5//DDX6//EIF4E//EIF5//NCBP2//OGFOD1//NF2//YWHAG//PSMA8//PSMA1//PSMC6//MUL1//BAK1//XBP1//TRAF4//G3BP2//CD40LG//TRIM71//SERPINB3//SERPINB4//RNF152//USP51//WIZ//RIPK3//VDAC1//TRPC5//TIMP2//LARP4B//UPF3A//EXOC8//ATP6V0E1//FBXW11//EBF3//CRLF3//RNF4//NCOA4//FUBP3//PIAS2//CHURC1//HAND2//ZNF488//PRAMEF19//MEX3B//TMEM100//CTDSPL2//NPPC//CALCR//PAX7//BTN2A2//MOB1B//RAP2C//RASSF2//OSBPL8//MTM1//CRTC1//PDE1B//NECAB3//NECAB1//DHCR24//TRIAP1//CDK12//CCNC//TFRC//ASPH//E2F5//HOXA9//PBX3//PLAGL1//ALX4//YES1//MIXL1//GRIK2//C14ORF39//FAM20C//NOVA2//DCUN1D5//GTPBP1//QKI//PCK1//CUL2//EIF3A//WDR48//NMD3//RAD52// |
| GO:0070555 | response to interleukin-1 | Biological process | 10 | 192 | 393 | 17653 | 2.33950911789652 | 0.0110375298156756 | 0.661929139382732 | 1.95712811025325 | 0.0254452926208651 | NOD1//MAP3K8//PSMA8//FBXW11//PSMA1//PSMC6//KLF2//PCK1//RORA//ZC3H12A// |
| GO:0010467 | gene expression | Biological process | 150 | 5761 | 393 | 17653 | 1.16955151181079 | 0.0110598835945954 | 0.661929139382732 | 1.95624944396178 | 0.381679389312977 | DHFR//GFI1//KLF2//ZHX1//IRX2//DR1//CUX2//NR6A1//SMAD5//MITF//DLL4//H2AFY2//PURB//RARB//SMO//TRPS1//UBE2I//NCBP2//UPF3A//EIF4E//ZC3H12A//DBR1//RBM7//SNRPF//NOVA2//RNPC3//SRSF11//NCOA1//RPL7L1//POP4//EIF3A//SUB1//EBF3//TAF5L//NFYC//NPM1//PBX3//YBX2//PLAGL1//RFX4//ALX4//HIF3A//TAF11//BTF3//XBP1//MIXL1//FUBP3//HAND2//CREB5//FSTL3//PCGF3//USP16//ZNF273//KAT7//FOXP4//ZNF488//ZNF597//ZNF558//ZNF320//E2F5//KDM2A//CRTC1//GABPB1//ALX3//ACAD8//ZNF544//HOXA9//APEX1//ZNF699//AFF3//PAX7//ATRX//COMMD8//PRKAA2//ARNTL2//RNF4//SP4//ZNF37A//ZNF75D//ZNF329//SAP30L//SLTM//NCOA4//PIAS2//ZNF251//CHURC1//REXO4//RORA//CSDE1//LZTR1//WDHD1//CKS2//CERS6//QRICH1//THAP10//ZNF704//SENP3//CCNC//CDK12//QKI//MRPS7//COA1//EIF1AX//EIF5//DDX3Y//GTPBP1//DDX6//OGFOD1//SEC11A//G3BP2//RPE65//MAPKBP1//EREG//TRIM71//NOD1//ATP7A//KIF1B//CALCR//ZNF804A//BAK1//THBS1//LARP4B//F2R//FBXW11//CRLF3//PRAMEF19//MEX3B//FKBP1A//TSN//C5AR1//TMEM100//CTDSPL2//ASZ1//EXD1//DHCR24//ST14//PSMA8//PSMA1//PSMC6//IL2//TRIAP1//YES1//C14ORF39//RIPK3//CD40LG//TSPAN5//PCK1//CUL2//INPP5B//ASPH// |
| GO:0051171 | regulation of nitrogen compound metabolic process | Biological process | 161 | 6240 | 393 | 17653 | 1.15895682455797 | 0.0111795508289968 | 0.663221949618468 | 1.95157564514431 | 0.409669211195929 | KPNA1//DHFR//GFI1//KLF2//ZHX1//IRX2//DR1//CUX2//NR6A1//SMAD5//MITF//DLL4//H2AFY2//PURB//RARB//SMO//TRPS1//UBE2I//STK24//MAP3K8//F2R//TAOK3//NOD1//THBS1//C5AR1//DUSP16//RBM7//NCOA1//FKBP1A//IL2//ZC3H12A//FAS//CDK2AP1//ATP7A//EREG//PCGF3//KAT7//ZNF597//ZNF558//ZNF320//KDM2A//ACAD8//TAF5L//ZNF544//APEX1//ZNF699//AFF3//NFYC//YBX2//ATRX//COMMD8//PRKAA2//ARNTL2//REXO4//RORA//TAF11//BTF3//ZNF37A//ZNF75D//CSDE1//ZNF329//SAP30L//LZTR1//ZNF251//FSTL3//USP16//ZNF273//SUB1//WDHD1//FOXP4//CKS2//CERS6//GABPB1//ALX3//NPM1//QRICH1//THAP10//RFX4//ZNF704//HIF3A//SP4//SLTM//CREB5//DDX6//EIF4E//EIF5//NCBP2//OGFOD1//NF2//YWHAG//PSMA8//PSMA1//PSMC6//MUL1//BAK1//XBP1//TRAF4//G3BP2//CD40LG//TRIM71//SERPINB3//SERPINB4//USP51//WIZ//TRPC5//TIMP2//LARP4B//UPF3A//FBXW11//EBF3//CRLF3//RNF4//NCOA4//FUBP3//PIAS2//CHURC1//HAND2//ZNF488//PRAMEF19//MEX3B//TMEM100//CTDSPL2//NPPC//CALCR//PAX7//MOB1B//RAP2C//RASSF2//RIPK3//OSBPL8//MTM1//CRTC1//PDE1B//NECAB3//NECAB1//DHCR24//TRIAP1//CDK12//CCNC//TFRC//ASPH//E2F5//HOXA9//PBX3//PLAGL1//ALX4//YES1//MIXL1//GRIK2//C14ORF39//NOVA2//DCUN1D5//GTPBP1//QKI//PCK1//CUL2//EIF3A//INPP5B//WDR48//NMD3//RAD52// |
| GO:0043030 | regulation of macrophage activation | Biological process | 4 | 40 | 393 | 17653 | 4.49185750636132 | 0.0117534631250673 | 0.691205835781132 | 1.92983415088092 | 0.0101781170483461 | CD200//ZC3H12A//THBS1//RORA// |
| GO:0000278 | mitotic cell cycle | Biological process | 35 | 1056 | 393 | 17653 | 1.48877852957051 | 0.0122106022618312 | 0.69996180107528 | 1.91326291488072 | 0.089058524173028 | TRIM71//EIF4E//CRLF3//MCM10//CUL2//DHFR//GFI1//FBXW11//YWHAG//CEP135//DYNLT1//NPM1//TRIAP1//PLAGL1//CKS2//TAOK3//CDC14A//MAP3K8//STK24//PSMA8//PSMA1//PSMC6//EREG//BTG3//TIMP2//MAP9//APEX1//CCSAP//TPRA1//ATRX//USP16//WDHD1//TUBB2B//CLTCL1//RAB6C// |
| GO:0010758 | regulation of macrophage chemotaxis | Biological process | 3 | 22 | 393 | 17653 | 6.12526023594726 | 0.012316346935969 | 0.69996180107528 | 1.90951808606839 | 0.00763358778625954 | THBS1//C5AR1//CYP19A1// |
| GO:0021516 | dorsal spinal cord development | Biological process | 3 | 22 | 393 | 17653 | 6.12526023594726 | 0.012316346935969 | 0.69996180107528 | 1.90951808606839 | 0.00763358778625954 | PAX7//PBX3//RFX4// |
| GO:0061157 | mRNA destabilization | Biological process | 3 | 22 | 393 | 17653 | 6.12526023594726 | 0.012316346935969 | 0.69996180107528 | 1.90951808606839 | 0.00763358778625954 | TRIM71//ZC3H12A//QKI// |
| GO:0021575 | hindbrain morphogenesis | Biological process | 4 | 41 | 393 | 17653 | 4.38230000620617 | 0.0128007027205171 | 0.701177862383904 | 1.89276618822199 | 0.0101781170483461 | RFX4//SMO//ATP7A//RORA// |
| GO:0035136 | forelimb morphogenesis | Biological process | 4 | 41 | 393 | 17653 | 4.38230000620617 | 0.0128007027205171 | 0.701177862383904 | 1.89276618822199 | 0.0101781170483461 | ALX3//HOXA9//ALX4//ATRX// |
| GO:0009059 | macromolecule biosynthetic process | Biological process | 144 | 5526 | 393 | 17653 | 1.17051661403552 | 0.012913372729515 | 0.701177862383904 | 1.88896031338679 | 0.366412213740458 | KAT7//MCM10//DHFR//GFI1//KLF2//ZHX1//IRX2//DR1//CUX2//NR6A1//SMAD5//MITF//DLL4//H2AFY2//PURB//RARB//SMO//TRPS1//UBE2I//NCOA1//EIF3A//RPL7L1//SUB1//NCBP2//EBF3//TAF5L//NFYC//NPM1//PBX3//YBX2//PLAGL1//RFX4//ALX4//HIF3A//TAF11//BTF3//XBP1//MIXL1//FUBP3//HAND2//CREB5//SNRPF//SRSF11//WDHD1//CDK2AP1//FSTL3//PCGF3//USP16//ZNF273//FOXP4//ZNF488//ZNF597//ZNF558//ZNF320//E2F5//KDM2A//CRTC1//GABPB1//ALX3//ACAD8//ZNF544//HOXA9//APEX1//ZNF699//AFF3//PAX7//ATRX//COMMD8//PRKAA2//ARNTL2//RNF4//SP4//ZNF37A//ZNF75D//ZNF329//SAP30L//SLTM//ZC3H12A//NCOA4//PIAS2//ZNF251//CHURC1//REXO4//RORA//CSDE1//LZTR1//CKS2//CERS6//QRICH1//THAP10//ZNF704//CCNC//CDK12//MRPS7//COA1//EIF1AX//EIF4E//EIF5//DDX3Y//GTPBP1//DDX6//OGFOD1//NUS1//XPNPEP2//VNN1//G3BP2//NF2//EREG//WIZ//LARP4B//UPF3A//THBS1//GALNT14//F2R//FBXW11//CRLF3//PRAMEF19//TRIM71//MEX3B//KERA//LMAN1//ZDHHC17//CANT1//TMEM100//CTDSPL2//NOD1//C5AR1//NECAB3//NECAB1//PSMC6//IL2//TRIAP1//YES1//C14ORF39//RIPK3//CD40LG//ATP7A//QKI//PCK1//PSMA8//PSMA1//CUL2//NMD3//RAP2C// |
| GO:0055072 | iron ion homeostasis | Biological process | 6 | 87 | 393 | 17653 | 3.09783276300781 | 0.0130457824501696 | 0.701177862383904 | 1.88452986799341 | 0.0152671755725191 | TMEM199//TFRC//NCOA4//STEAP2//B2M//SFXN1// |
| GO:0048167 | regulation of synaptic plasticity | Biological process | 9 | 168 | 393 | 17653 | 2.40635223555071 | 0.0130920053259058 | 0.701177862383904 | 1.88299382669041 | 0.0229007633587786 | SYP//GRIK2//SHISA6//SHISA9//SNAP47//SLC24A2//CRTC1//FGF14//YWHAG// |
| GO:0090305 | nucleic acid phosphodiester bond hydrolysis | Biological process | 13 | 289 | 393 | 17653 | 2.02055873988572 | 0.0131145206127536 | 0.701177862383904 | 1.88224757995285 | 0.0330788804071247 | EXOG//KPNA1//POP4//ZC3H12A//APEX1//DBR1//NCBP2//APEX2//N4BP2//REXO4//AEN//TSN//PGBD5// |
| GO:0050863 | regulation of T cell activation | Biological process | 14 | 321 | 393 | 17653 | 1.9590655790984 | 0.0131762366084812 | 0.701177862383904 | 1.88020861504997 | 0.0356234096692112 | MAP3K8//CD274//HLA-DPB1//YES1//CD3G//CD40LG//VNN1//CD209//IL2//TFRC//CD1D//XBP1//RIPK3//BTN2A2// |
| GO:0050767 | regulation of neurogenesis | Biological process | 26 | 732 | 393 | 17653 | 1.59546851318845 | 0.0132032423082651 | 0.701177862383904 | 1.87931940639422 | 0.0661577608142494 | SMO//ZNF804A//DYNLT1//GFI1//PMP22//B2M//PTPRG//NF2//YWHAG//DDX6//EIF4E//RARB//TIMP2//NCOA1//TRPC5//STK24//WDR1//ZNF488//SEMA3C//CUX2//LTA//IL2//CRTC1//TUBB2B//MUL1//DLL4// |
| GO:0034645 | cellular macromolecule biosynthetic process | Biological process | 141 | 5401 | 393 | 17653 | 1.17265674578216 | 0.0132708511585302 | 0.701177862383904 | 1.87710122170238 | 0.358778625954198 | KAT7//MCM10//DHFR//GFI1//KLF2//ZHX1//IRX2//DR1//CUX2//NR6A1//SMAD5//MITF//DLL4//H2AFY2//PURB//RARB//SMO//TRPS1//UBE2I//NCOA1//EIF3A//RPL7L1//SUB1//NCBP2//EBF3//TAF5L//NFYC//NPM1//PBX3//YBX2//PLAGL1//RFX4//ALX4//HIF3A//TAF11//BTF3//XBP1//MIXL1//FUBP3//HAND2//CREB5//SNRPF//SRSF11//WDHD1//CDK2AP1//FSTL3//PCGF3//USP16//ZNF273//FOXP4//ZNF488//ZNF597//ZNF558//ZNF320//E2F5//KDM2A//CRTC1//GABPB1//ALX3//ACAD8//ZNF544//HOXA9//APEX1//ZNF699//AFF3//PAX7//ATRX//COMMD8//PRKAA2//ARNTL2//RNF4//SP4//ZNF37A//ZNF75D//ZNF329//SAP30L//SLTM//ZC3H12A//NCOA4//PIAS2//ZNF251//CHURC1//REXO4//RORA//CSDE1//LZTR1//CKS2//CERS6//QRICH1//THAP10//ZNF704//CCNC//CDK12//MRPS7//COA1//EIF1AX//EIF4E//EIF5//DDX3Y//GTPBP1//DDX6//OGFOD1//NUS1//XPNPEP2//VNN1//G3BP2//NF2//EREG//WIZ//LARP4B//UPF3A//THBS1//GALNT14//F2R//FBXW11//CRLF3//PRAMEF19//TRIM71//MEX3B//LMAN1//ZDHHC17//CANT1//TMEM100//CTDSPL2//NOD1//C5AR1//NECAB3//NECAB1//PSMC6//IL2//TRIAP1//YES1//C14ORF39//RIPK3//CD40LG//ATP7A//QKI//PCK1//PSMA8//PSMA1//CUL2// |
| GO:0021513 | spinal cord dorsal/ventral patterning | Biological process | 3 | 23 | 393 | 17653 | 5.85894457351477 | 0.0139340600900354 | 0.726623339712137 | 1.85592232092112 | 0.00763358778625954 | SMO//RFX4//DLL4// |
| GO:0055082 | cellular chemical homeostasis | Biological process | 27 | 773 | 393 | 17653 | 1.56895410959581 | 0.0142699768866958 | 0.726623339712137 | 1.84557673031833 | 0.0687022900763359 | SLC24A2//GRIK2//ATP7A//TMEM199//TFRC//NCOA4//SLC30A5//ATP6V0E1//CCR5//F2R//IL2//TRPC5//C5AR1//BAK1//TRPC3//SLC41A1//XBP1//OXCT1//VSNL1//FKBP1A//SLC9C2//SLC9A2//F2RL2//SLC30A2//GCLM//PCK1//CALCR// |
| GO:0006915 | apoptotic process | Biological process | 58 | 1958 | 393 | 17653 | 1.3305808752245 | 0.0143327896089194 | 0.726623339712137 | 1.84366927409298 | 0.147582697201018 | BAK1//IL2//HAND2//KPNA1//NOD1//F2R//FAS//MUL1//DHCR24//NPM1//PAX7//TRIAP1//UFM1//PRKAA2//RARB//PRAMEF19//SMO//PRDX2//THBS1//XBP1//CD40LG//STK24//GCLM//ZC3H12A//MAP3K8//PPP1R13B//ALX3//APEX1//NF2//TAOK3//ALX4//TRAF4//AEN//GZMK//LTA//NCOA1//RASSF2//GRIK2//GFRAL//C5AR1//CD3G//RIPK3//CUL2//YWHAG//VNN1//CD274//CCR5//RNF152//KIF1B//GSDMA//EMC4//PDE1B//PLAGL1//RFK//HIF3A//VDAC1//SLTM// |
| GO:0006310 | DNA recombination | Biological process | 12 | 261 | 393 | 17653 | 2.06522184200521 | 0.0143558001935461 | 0.726623339712137 | 1.84297259488075 | 0.0305343511450382 | KPNA1//WDR48//RAD52//USP51//C14ORF39//CD40LG//TFRC//IL2//APEX2//APEX1//ATRX//TSN// |
| GO:0045595 | regulation of cell differentiation | Biological process | 53 | 1761 | 393 | 17653 | 1.35189351412351 | 0.0143936508136094 | 0.726623339712137 | 1.8418290373969 | 0.134860050890585 | SMO//PAX7//SERPINB3//XBP1//ZNF804A//DYNLT1//GFI1//PMP22//B2M//PTPRG//NF2//TRPS1//RARB//VNN1//IL2//PRAMEF19//RORA//ZC3H12A//TMEM100//H2AFY2//PURB//HOXA9//THBS1//YWHAG//DDX6//EIF4E//TIMP2//NCOA1//PIAS2//RASSF2//HAND2//SMAD5//NPPC//FAM20C//MITF//FSTL3//TRPC5//STK24//WDR1//ZNF488//SEMA3C//DLL4//CUX2//EREG//LTA//CRTC1//MIXL1//PSMA8//PSMA1//PSMC6//TUBB2B//MUL1//CDK12// |
| GO:0008152 | metabolic process | Biological process | 282 | 11746 | 393 | 17653 | 1.07841292081891 | 0.0143970911609384 | 0.726623339712137 | 1.84172524543007 | 0.717557251908397 | KPNA1//KAT7//MCM10//DHFR//GFI1//KLF2//ZHX1//IRX2//DR1//CUX2//NR6A1//SMAD5//MITF//DLL4//H2AFY2//PURB//RARB//SMO//TRPS1//UBE2I//CCR5//PSMA8//EREG//IL2//TAOK3//PSMA1//PSMC6//STK24//NCBP2//UPF3A//MAP3K8//F2R//NOD1//THBS1//C5AR1//DUSP16//FBXW11//UBE2W//EIF4E//ZC3H12A//COPS7B//DBR1//RBM7//SNRPF//NOVA2//RNPC3//SRSF11//FKBP1A//NCOA1//RPL7L1//APEX1//WDR48//RAD52//EXOG//RPE65//LRAT//POP4//EIF3A//FAS//CDK2AP1//ATP7A//SUB1//EBF3//TAF5L//NFYC//NPM1//PBX3//YBX2//PLAGL1//RFX4//ALX4//HIF3A//TAF11//BTF3//XBP1//MIXL1//FUBP3//HAND2//CREB5//ASB15//WDSUB1//RNF152//RNF4//MUL1//CUL2//YDJC//ALDH1B1//HYAL4//RPE//ALDOB//PCK1//H6PD//MOGAT3//COX4I1//IFI30//CPOX//DHCR24//KDM2A//STEAP2//ACAD8//GPX3//RFESD//MARC2//OGFOD1//SCD//PRDX2//PYROXD1//PYROXD2//CYP19A1//ASPH//ACPP//CMPK1//NPPC//PDE1B//WDHD1//USP51//ATRX//APEX2//TSN//FSTL3//PCGF3//USP16//ZNF273//FOXP4//ZNF488//ZNF597//ZNF558//ZNF320//E2F5//CRTC1//GABPB1//ALX3//ZNF544//HOXA9//ZNF699//AFF3//PAX7//COMMD8//PRKAA2//ARNTL2//SP4//ZNF37A//ZNF75D//ZNF329//SAP30L//SLTM//NCOA4//PIAS2//ZNF251//CHURC1//REXO4//RORA//CSDE1//LZTR1//CKS2//CERS6//QRICH1//THAP10//ZNF704//SENP3//CCNC//CDK12//QKI//MRPS7//COA1//EIF1AX//EIF5//DDX3Y//GTPBP1//DDX6//RIPK3//YES1//SEC11A//SPEG//FAM20C//MEX3B//CDKL1//RASSF2//NF2//YWHAG//MTM1//CTDSPL2//PTPN7//PCMTD1//NUS1//XPNPEP2//VNN1//C2ORF66//CPQ//GZMK//NRIP3//PRSS22//ST14//XPNPEP1//CTSF//ADAM23//NGLY1//GCLM//MCCC2//BTN2A1//TPRA1//ABCD3//AGPAT5//ETNK2//PIP5K1A//DGKA//AGPAT4//PIP5K1C//SYNJ1//IDI2//RFK//NAT2//LIX1L//BAK1//TRAF4//G3BP2//CD40LG//MAPKBP1//PGLYRP4//GALNT14//TRIM71//SERPINB3//SERPINB4//WIZ//KIF1B//CALCR//ZNF804A//VDAC1//C14ORF39//TRPC5//TIMP2//PLCXD3//LARP4B//EBPL//EXOC8//ATP6V0E1//N4BP2//ENOPH1//CRLF3//PRAMEF19//TEX30//OTUD3//LYZL6//CAMK1G//KERA//LMAN1//ZDHHC17//CRAT//NECAB3//BCHE//DCUN1D5//CANT1//TMEM100//ASZ1//EXD1//BTN2A2//TFRC//MOB1B//RAP2C//OSBPL8//METTL7B//CLTA//SATL1//PTPRG//PTP4A2//CDC14A//OXCT1//NECAB1//TRIAP1//INPP5B//SUMF2//LTBP1//VWA1//B2M//GRIK2//SULT6B1//TSPAN5//UFM1//AEN//PGBD5//NMD3//TMEM199// |
| GO:1903313 | positive regulation of mRNA metabolic process | Biological process | 5 | 65 | 393 | 17653 | 3.45527500489333 | 0.0147818336433625 | 0.740515118000449 | 1.83027168963536 | 0.0127226463104326 | NCBP2//ZC3H12A//GTPBP1//TRIM71//QKI// |
| GO:0050798 | activated T cell proliferation | Biological process | 4 | 43 | 393 | 17653 | 4.17847209894077 | 0.0150693347049535 | 0.749366989776474 | 1.82190592090007 | 0.0101781170483461 | IL2//RIPK3//BTN2A2//CD274// |
| GO:0044772 | mitotic cell cycle phase transition | Biological process | 21 | 565 | 393 | 17653 | 1.6695399581166 | 0.01566188983386 | 0.751612950206933 | 1.80515583519648 | 0.0534351145038168 | TRIM71//EIF4E//CRLF3//MCM10//CUL2//DHFR//GFI1//FBXW11//YWHAG//CEP135//NPM1//TRIAP1//PLAGL1//TAOK3//CDC14A//PSMA8//PSMA1//PSMC6//APEX1//TPRA1//CKS2// |
| GO:0019682 | glyceraldehyde-3-phosphate metabolic process | Biological process | 3 | 24 | 393 | 17653 | 5.61482188295165 | 0.0156666469140378 | 0.751612950206933 | 1.80502394434542 | 0.00763358778625954 | RPE//H6PD//ALDOB// |
| GO:0033233 | regulation of protein sumoylation | Biological process | 3 | 24 | 393 | 17653 | 5.61482188295165 | 0.0156666469140378 | 0.751612950206933 | 1.80502394434542 | 0.00763358778625954 | RNF4//MUL1//UBE2I// |
| GO:0050779 | RNA destabilization | Biological process | 3 | 24 | 393 | 17653 | 5.61482188295165 | 0.0156666469140378 | 0.751612950206933 | 1.80502394434542 | 0.00763358778625954 | TRIM71//ZC3H12A//QKI// |
| GO:0055080 | cation homeostasis | Biological process | 25 | 707 | 393 | 17653 | 1.58835131059453 | 0.0156908456445306 | 0.751612950206933 | 1.80435364985751 | 0.0636132315521628 | SLC24A2//GRIK2//ATP7A//TMEM199//TFRC//NCOA4//SLC30A5//ATP6V0E1//CCR5//F2R//IL2//TRPC5//C5AR1//BAK1//TRPC3//SLC41A1//FKBP1A//SLC9C2//SLC9A2//F2RL2//STEAP2//B2M//SFXN1//SLC30A2//CALCR// |
| GO:0012501 | programmed cell death | Biological process | 61 | 2088 | 393 | 17653 | 1.31227637877414 | 0.0158278941466533 | 0.751612950206933 | 1.80057686285876 | 0.155216284987277 | BAK1//IL2//HAND2//NOD1//DHCR24//RNF152//KIF1B//GSDMA//FAS//LTA//EMC4//TRIAP1//PDE1B//PLAGL1//RFK//HIF3A//C5AR1//VDAC1//MUL1//SLTM//ZC3H12A//TRAF4//KPNA1//F2R//NPM1//PAX7//UFM1//PRKAA2//RARB//PRAMEF19//SMO//PRDX2//THBS1//XBP1//CD40LG//STK24//GCLM//MAP3K8//PPP1R13B//ALX3//APEX1//NF2//TAOK3//ALX4//AEN//GZMK//NCOA1//RASSF2//GRIK2//GFRAL//RIPK3//CD3G//KRT6C//ST14//TCHH//CUL2//YWHAG//VNN1//CD274//CCR5// |
| GO:0009791 | post-embryonic development | Biological process | 6 | 91 | 393 | 17653 | 2.96166428990856 | 0.0160035878796094 | 0.751612950206933 | 1.79578264099187 | 0.0152671755725191 | ATRX//BAK1//SEMA3C//NPPC//ETNK2//ALX4// |
| GO:0032649 | regulation of interferon-gamma production | Biological process | 6 | 91 | 393 | 17653 | 2.96166428990856 | 0.0160035878796094 | 0.751612950206933 | 1.79578264099187 | 0.0152671755725191 | CD274//HLA-DPB1//IL2//LTA//ZC3H12A//RIPK3// |
| GO:0002478 | antigen processing and presentation of exogenous peptide antigen | Biological process | 9 | 174 | 393 | 17653 | 2.32337457225586 | 0.0161337875592284 | 0.752502105262494 | 1.79226366590441 | 0.0229007633587786 | PSMA8//B2M//PSMA1//PSMC6//IFI30//CLTA//DYNC1LI2//HLA-DPB1//CTSF// |
| GO:0006775 | fat-soluble vitamin metabolic process | Biological process | 4 | 44 | 393 | 17653 | 4.08350682396484 | 0.0162926493229327 | 0.752550918413922 | 1.78800828998383 | 0.0101781170483461 | RPE65//LRAT//GFI1//UBE2I// |
| GO:0006695 | cholesterol biosynthetic process | Biological process | 5 | 67 | 393 | 17653 | 3.35213246743382 | 0.0166765112838622 | 0.752550918413922 | 1.77789479834089 | 0.0127226463104326 | DHCR24//NFYC//SCD//PRKAA2//IDI2// |
| GO:0071359 | cellular response to dsRNA | Biological process | 5 | 67 | 393 | 17653 | 3.35213246743382 | 0.0166765112838622 | 0.752550918413922 | 1.77789479834089 | 0.0127226463104326 | TRIM71//TSN//NPM1//MUL1//ZC3H12A// |
| GO:0008219 | cell death | Biological process | 64 | 2216 | 393 | 17653 | 1.29728736645814 | 0.0169709722681614 | 0.752550918413922 | 1.77029327620903 | 0.162849872773537 | BAK1//IL2//HAND2//NOD1//DHCR24//RNF152//KIF1B//GSDMA//FAS//LTA//EMC4//TRIAP1//PDE1B//PLAGL1//RFK//HIF3A//C5AR1//VDAC1//MUL1//SLTM//ZC3H12A//TRAF4//KPNA1//F2R//NPM1//PAX7//UFM1//PRKAA2//RARB//PRAMEF19//SMO//PRDX2//THBS1//XBP1//CD40LG//STK24//GCLM//CALCR//MAP3K8//PPP1R13B//ALX3//APEX1//NF2//TAOK3//ALX4//AEN//GZMK//NCOA1//RASSF2//GRIK2//GFRAL//RIPK3//DYNLT1//CD3G//KRT6C//ST14//TCHH//CUL2//YWHAG//VNN1//CD274//CCR5//PMP22// |
| GO:0019430 | removal of superoxide radicals | Biological process | 3 | 25 | 393 | 17653 | 5.39022900763359 | 0.0175149277483434 | 0.752550918413922 | 1.7565916498713 | 0.00763358778625954 | DHFR//ATP7A//PRDX2// |
| GO:0021511 | spinal cord patterning | Biological process | 3 | 25 | 393 | 17653 | 5.39022900763359 | 0.0175149277483434 | 0.752550918413922 | 1.7565916498713 | 0.00763358778625954 | SMO//RFX4//DLL4// |
| GO:0046856 | phosphatidylinositol dephosphorylation | Biological process | 3 | 25 | 393 | 17653 | 5.39022900763359 | 0.0175149277483434 | 0.752550918413922 | 1.7565916498713 | 0.00763358778625954 | INPP5B//MTM1//SYNJ1// |
| GO:0071407 | cellular response to organic cyclic compound | Biological process | 21 | 572 | 393 | 17653 | 1.64910852506272 | 0.0177079354646787 | 0.752550918413922 | 1.7518320694459 | 0.0534351145038168 | TRIM71//TSN//NR6A1//RNF4//NCOA4//NCOA1//PIAS2//UFM1//RORA//RARB//NPM1//APEX1//PCK1//MUL1//SMO//KLF2//EIF4E//GJB2//GCLM//ZC3H12A//SMAD5// |
| GO:0044249 | cellular biosynthetic process | Biological process | 167 | 6579 | 393 | 17653 | 1.14020398778286 | 0.0178303457733006 | 0.752550918413922 | 1.74884023472816 | 0.424936386768448 | KAT7//MCM10//DHFR//GFI1//KLF2//ZHX1//IRX2//DR1//CUX2//NR6A1//SMAD5//MITF//DLL4//H2AFY2//PURB//RARB//SMO//TRPS1//UBE2I//NCOA1//EIF3A//RPL7L1//SUB1//NCBP2//EBF3//TAF5L//NFYC//NPM1//PBX3//YBX2//PLAGL1//RFX4//ALX4//HIF3A//TAF11//BTF3//XBP1//MIXL1//FUBP3//HAND2//CREB5//SNRPF//SRSF11//ALDOB//NPPC//PDE1B//CMPK1//WDHD1//CDK2AP1//FSTL3//PCGF3//USP16//ZNF273//FOXP4//ZNF488//ZNF597//ZNF558//ZNF320//E2F5//KDM2A//CRTC1//GABPB1//ALX3//ACAD8//ZNF544//HOXA9//APEX1//ZNF699//AFF3//PAX7//ATRX//COMMD8//PRKAA2//ARNTL2//RNF4//SP4//ZNF37A//ZNF75D//ZNF329//SAP30L//SLTM//ZC3H12A//NCOA4//PIAS2//ZNF251//CHURC1//REXO4//RORA//CSDE1//LZTR1//CKS2//CERS6//QRICH1//THAP10//ZNF704//CCNC//CDK12//MRPS7//COA1//EIF1AX//EIF4E//EIF5//DDX3Y//GTPBP1//DDX6//OGFOD1//NUS1//XPNPEP2//VNN1//ABCD3//SCD//ETNK2//DGKA//AGPAT5//AGPAT4//PIP5K1C//MTM1//PIP5K1A//SYNJ1//CYP19A1//GCLM//CPOX//G3BP2//NF2//IDI2//RFK//EREG//WIZ//LARP4B//UPF3A//THBS1//GALNT14//F2R//FBXW11//CRLF3//PRAMEF19//TRIM71//MEX3B//KERA//LMAN1//ZDHHC17//MOGAT3//ENOPH1//CANT1//TMEM100//CTDSPL2//NOD1//QKI//C5AR1//NECAB3//NECAB1//TIMP2//PSMC6//IL2//TRIAP1//YES1//PCK1//C14ORF39//RIPK3//CD40LG//ATP7A//PSMA8//PSMA1//CUL2//RPE65//NMD3//RAP2C// |
| GO:0048872 | homeostasis of number of cells | Biological process | 11 | 238 | 393 | 17653 | 2.07606859537708 | 0.0179822862987137 | 0.752550918413922 | 1.7451550921472 | 0.0279898218829517 | BAK1//SMAD5//SFXN1//RIPK3//KLF2//B2M//IL2//F2R//SMO//WDR48//RASSF2// |
| GO:0014070 | response to organic cyclic compound | Biological process | 31 | 937 | 393 | 17653 | 1.48610013550908 | 0.0182054681843766 | 0.752550918413922 | 1.73979814796998 | 0.0788804071246819 | TRIM71//TSN//NR6A1//RNF4//NCOA4//NCOA1//PIAS2//FKBP1A//DHFR//GJB2//THBS1//UFM1//TRPC3//RORA//RARB//BCHE//CALCR//NPM1//CCR5//APEX1//PCK1//MUL1//SMO//SMAD5//KLF2//EIF4E//GCLM//ZC3H12A//KIF1B//BAK1//ABCD3// |
| GO:1903037 | regulation of leukocyte cell-cell adhesion | Biological process | 13 | 302 | 393 | 17653 | 1.93358104578468 | 0.0182260498272376 | 0.752550918413922 | 1.73930744676609 | 0.0330788804071247 | MAP3K8//CD274//HLA-DPB1//YES1//CD3G//CD40LG//VNN1//CD209//IL2//TFRC//CD1D//XBP1//BTN2A2// |
| GO:0007159 | leukocyte cell-cell adhesion | Biological process | 14 | 335 | 393 | 17653 | 1.87719418176294 | 0.0184048572944679 | 0.752550918413922 | 1.73506754558843 | 0.0356234096692112 | MAP3K8//CD274//HLA-DPB1//YES1//CD3G//CD40LG//VNN1//CD209//IL2//TFRC//CD1D//XBP1//BTN2A2//CLEC4M// |
| GO:0050804 | modulation of chemical synaptic transmission | Biological process | 14 | 335 | 393 | 17653 | 1.87719418176294 | 0.0184048572944679 | 0.752550918413922 | 1.73506754558843 | 0.0356234096692112 | STX1A//KCNC4//FGF14//YWHAG//SYP//GRIK2//SHISA6//SHISA9//BCHE//SNAP47//SLC24A2//F2R//CRTC1//CUX2// |
| GO:0099177 | regulation of trans-synaptic signaling | Biological process | 14 | 335 | 393 | 17653 | 1.87719418176294 | 0.0184048572944679 | 0.752550918413922 | 1.73506754558843 | 0.0356234096692112 | STX1A//KCNC4//FGF14//YWHAG//SYP//GRIK2//SHISA6//SHISA9//BCHE//SNAP47//SLC24A2//F2R//CRTC1//CUX2// |
| GO:1902653 | secondary alcohol biosynthetic process | Biological process | 5 | 69 | 393 | 17653 | 3.25496920750821 | 0.0187258976183444 | 0.752550918413922 | 1.72755735539883 | 0.0127226463104326 | DHCR24//PRKAA2//IDI2//NFYC//SCD// |
| GO:0001974 | blood vessel remodeling | Biological process | 4 | 46 | 393 | 17653 | 3.90596304900985 | 0.0189216102944979 | 0.752550918413922 | 1.72304190639852 | 0.0101781170483461 | SEMA3C//ATP7A//DLL4//BAK1// |
| GO:0044003 | modification by symbiont of host morphology or physiology | Biological process | 4 | 46 | 393 | 17653 | 3.90596304900985 | 0.0189216102944979 | 0.752550918413922 | 1.72304190639852 | 0.0101781170483461 | CLEC4M//CD209//KPNA1//ZC3H12A// |
| GO:1901617 | organic hydroxy compound biosynthetic process | Biological process | 11 | 240 | 393 | 17653 | 2.05876802374894 | 0.0190051317635355 | 0.752550918413922 | 1.72112911506493 | 0.0279898218829517 | DHCR24//PRKAA2//IDI2//DHFR//GFI1//NUS1//NFYC//SCD//PCK1//CYP19A1//HAND2// |
| GO:0098771 | inorganic ion homeostasis | Biological process | 25 | 720 | 393 | 17653 | 1.55967274526435 | 0.0191833120301963 | 0.752550918413922 | 1.71707640903917 | 0.0636132315521628 | SLC24A2//GRIK2//ATP7A//TMEM199//TFRC//NCOA4//SLC30A5//ATP6V0E1//CCR5//F2R//IL2//TRPC5//C5AR1//BAK1//TRPC3//SLC41A1//FKBP1A//SLC9C2//SLC9A2//F2RL2//STEAP2//B2M//SFXN1//SLC30A2//CALCR// |
| GO:1990823 | response to leukemia inhibitory factor | Biological process | 6 | 95 | 393 | 17653 | 2.83696263559662 | 0.0193952784544185 | 0.752550918413922 | 1.71230398093377 | 0.0152671755725191 | GCLM//TFRC//XBP1//LAPTM5//ADAM23//LRAT// |
| GO:1990830 | cellular response to leukemia inhibitory factor | Biological process | 6 | 95 | 393 | 17653 | 2.83696263559662 | 0.0193952784544185 | 0.752550918413922 | 1.71230398093377 | 0.0152671755725191 | GCLM//TFRC//XBP1//LAPTM5//ADAM23//LRAT// |
| GO:0031639 | plasminogen activation | Biological process | 3 | 26 | 393 | 17653 | 5.18291250733999 | 0.0194794817324794 | 0.752550918413922 | 1.71042260206308 | 0.00763358778625954 | THBS1//DHCR24//ST14// |
| GO:0036314 | response to sterol | Biological process | 3 | 26 | 393 | 17653 | 5.18291250733999 | 0.0194794817324794 | 0.752550918413922 | 1.71042260206308 | 0.00763358778625954 | RORA//CCR5//SMO// |
| GO:1905521 | regulation of macrophage migration | Biological process | 3 | 26 | 393 | 17653 | 5.18291250733999 | 0.0194794817324794 | 0.752550918413922 | 1.71042260206308 | 0.00763358778625954 | THBS1//C5AR1//CYP19A1// |
| GO:0002475 | antigen processing and presentation via MHC class Ib | Biological process | 2 | 10 | 393 | 17653 | 8.98371501272265 | 0.0197662079372669 | 0.752550918413922 | 1.70407664024033 | 0.00508905852417303 | B2M//CD1D// |
| GO:0006828 | manganese ion transport | Biological process | 2 | 10 | 393 | 17653 | 8.98371501272265 | 0.0197662079372669 | 0.752550918413922 | 1.70407664024033 | 0.00508905852417303 | TRPC3//TRPC5// |
| GO:0042428 | serotonin metabolic process | Biological process | 2 | 10 | 393 | 17653 | 8.98371501272265 | 0.0197662079372669 | 0.752550918413922 | 1.70407664024033 | 0.00508905852417303 | PDE1B//ATP7A// |
| GO:0061088 | regulation of sequestering of zinc ion | Biological process | 2 | 10 | 393 | 17653 | 8.98371501272265 | 0.0197662079372669 | 0.752550918413922 | 1.70407664024033 | 0.00508905852417303 | SLC30A2//SLC30A5// |
| GO:0070070 | proton-transporting V-type ATPase complex assembly | Biological process | 2 | 10 | 393 | 17653 | 8.98371501272265 | 0.0197662079372669 | 0.752550918413922 | 1.70407664024033 | 0.00508905852417303 | TMEM199//ALDOB// |
| GO:0070072 | vacuolar proton-transporting V-type ATPase complex assembly | Biological process | 2 | 10 | 393 | 17653 | 8.98371501272265 | 0.0197662079372669 | 0.752550918413922 | 1.70407664024033 | 0.00508905852417303 | TMEM199//ALDOB// |
| GO:0071281 | cellular response to iron ion | Biological process | 2 | 10 | 393 | 17653 | 8.98371501272265 | 0.0197662079372669 | 0.752550918413922 | 1.70407664024033 | 0.00508905852417303 | B2M//ATP7A// |
| GO:0070227 | lymphocyte apoptotic process | Biological process | 5 | 70 | 393 | 17653 | 3.20846964740094 | 0.0198100224230795 | 0.752550918413922 | 1.70311503288073 | 0.0127226463104326 | BAK1//IL2//CD3G//RIPK3//CD274// |
| GO:0032874 | positive regulation of stress-activated MAPK cascade | Biological process | 8 | 151 | 393 | 17653 | 2.37979205635037 | 0.0199181745373491 | 0.752550918413922 | 1.70075046634826 | 0.0203562340966921 | MUL1//CD40LG//TAOK3//NOD1//TRAF4//RASSF2//ZC3H12A//HAND2// |
| GO:0061013 | regulation of mRNA catabolic process | Biological process | 9 | 181 | 393 | 17653 | 2.23352030703049 | 0.0203032654872287 | 0.755404879609458 | 1.6924341064695 | 0.0229007633587786 | PSMA8//APEX1//PSMA1//PSMC6//ZC3H12A//GTPBP1//TRIM71//QKI//NPM1// |
| GO:0021695 | cerebellar cortex development | Biological process | 4 | 47 | 393 | 17653 | 3.8228574522224 | 0.0203288020240901 | 0.755404879609458 | 1.69188821357331 | 0.0101781170483461 | RFX4//SMO//ATP7A//RORA// |
| GO:1901028 | regulation of mitochondrial outer membrane permeabilization involved in apoptotic signaling pathway | Biological process | 4 | 47 | 393 | 17653 | 3.8228574522224 | 0.0203288020240901 | 0.755404879609458 | 1.69188821357331 | 0.0101781170483461 | PPP1R13B//YWHAG//BAK1//MUL1// |
| GO:0070304 | positive regulation of stress-activated protein kinase signaling cascade | Biological process | 8 | 152 | 393 | 17653 | 2.36413552966385 | 0.0206325637913661 | 0.762502890278737 | 1.68544680347317 | 0.0203562340966921 | MUL1//CD40LG//TAOK3//NOD1//TRAF4//RASSF2//ZC3H12A//HAND2// |
| GO:0034654 | nucleobase-containing compound biosynthetic process | Biological process | 118 | 4488 | 393 | 17653 | 1.18101422849963 | 0.0210069316039914 | 0.771857551112333 | 1.67763737856848 | 0.300254452926209 | DHFR//GFI1//KLF2//ZHX1//IRX2//DR1//CUX2//NR6A1//SMAD5//MITF//DLL4//H2AFY2//PURB//RARB//SMO//TRPS1//UBE2I//NCOA1//SUB1//NCBP2//EBF3//TAF5L//NFYC//NPM1//PBX3//YBX2//PLAGL1//RFX4//ALX4//HIF3A//TAF11//BTF3//XBP1//MIXL1//FUBP3//HAND2//CREB5//SNRPF//SRSF11//ALDOB//NPPC//PDE1B//CMPK1//FSTL3//PCGF3//USP16//ZNF273//KAT7//FOXP4//ZNF488//ZNF597//ZNF558//ZNF320//E2F5//KDM2A//CRTC1//GABPB1//ALX3//ACAD8//ZNF544//HOXA9//APEX1//ZNF699//AFF3//PAX7//ATRX//COMMD8//PRKAA2//ARNTL2//RNF4//SP4//ZNF37A//ZNF75D//ZNF329//SAP30L//SLTM//ZC3H12A//NCOA4//PIAS2//ZNF251//CHURC1//REXO4//RORA//CSDE1//LZTR1//WDHD1//CKS2//CERS6//QRICH1//THAP10//ZNF704//CCNC//CDK12//G3BP2//EREG//RFK//F2R//FBXW11//CRLF3//PRAMEF19//TMEM100//CTDSPL2//NOD1//C5AR1//TIMP2//PSMC6//IL2//TRIAP1//YES1//C14ORF39//RIPK3//CD40LG//PCK1//PSMA8//PSMA1//CUL2//NMD3//RAP2C// |
| GO:1901362 | organic cyclic compound biosynthetic process | Biological process | 123 | 4704 | 393 | 17653 | 1.17452906735213 | 0.0213660738472941 | 0.771857551112333 | 1.67027527489292 | 0.312977099236641 | DHFR//GFI1//KLF2//ZHX1//IRX2//DR1//CUX2//NR6A1//SMAD5//MITF//DLL4//H2AFY2//PURB//RARB//SMO//TRPS1//UBE2I//NCOA1//SUB1//NCBP2//EBF3//TAF5L//NFYC//NPM1//PBX3//YBX2//PLAGL1//RFX4//ALX4//HIF3A//TAF11//BTF3//XBP1//MIXL1//FUBP3//HAND2//CREB5//SNRPF//SRSF11//ALDOB//NPPC//PDE1B//CMPK1//FSTL3//PCGF3//USP16//ZNF273//KAT7//FOXP4//ZNF488//ZNF597//ZNF558//ZNF320//E2F5//KDM2A//CRTC1//GABPB1//ALX3//ACAD8//ZNF544//HOXA9//APEX1//ZNF699//AFF3//PAX7//ATRX//COMMD8//PRKAA2//ARNTL2//RNF4//SP4//ZNF37A//ZNF75D//ZNF329//SAP30L//SLTM//ZC3H12A//NCOA4//PIAS2//ZNF251//CHURC1//REXO4//RORA//CSDE1//LZTR1//WDHD1//CKS2//CERS6//QRICH1//THAP10//ZNF704//CCNC//CDK12//CYP19A1//DHCR24//IDI2//CPOX//G3BP2//RFK//EREG//F2R//FBXW11//CRLF3//PRAMEF19//TMEM100//CTDSPL2//NOD1//C5AR1//SCD//TIMP2//PSMC6//IL2//TRIAP1//YES1//C14ORF39//RIPK3//CD40LG//PCK1//PSMA8//PSMA1//CUL2//NMD3//RAP2C// |
| GO:0032770 | positive regulation of monooxygenase activity | Biological process | 3 | 27 | 393 | 17653 | 4.99095278484591 | 0.0215606603283795 | 0.771857551112333 | 1.66633794234523 | 0.00763358778625954 | NOD1//NUS1//DHFR// |
| GO:0071450 | cellular response to oxygen radical | Biological process | 3 | 27 | 393 | 17653 | 4.99095278484591 | 0.0215606603283795 | 0.771857551112333 | 1.66633794234523 | 0.00763358778625954 | ATP7A//PRDX2//DHFR// |
| GO:0071451 | cellular response to superoxide | Biological process | 3 | 27 | 393 | 17653 | 4.99095278484591 | 0.0215606603283795 | 0.771857551112333 | 1.66633794234523 | 0.00763358778625954 | ATP7A//PRDX2//DHFR// |
| GO:0080090 | regulation of primary metabolic process | Biological process | 162 | 6394 | 393 | 17653 | 1.13806837039496 | 0.0216933828627197 | 0.771857551112333 | 1.66367271889147 | 0.412213740458015 | KPNA1//DHFR//GFI1//KLF2//ZHX1//IRX2//DR1//CUX2//NR6A1//SMAD5//MITF//DLL4//H2AFY2//PURB//RARB//SMO//TRPS1//UBE2I//STK24//MAP3K8//F2R//TAOK3//NOD1//THBS1//C5AR1//DUSP16//RBM7//NCOA1//FKBP1A//IL2//ZC3H12A//FAS//CDK2AP1//ATP7A//EREG//PCGF3//KAT7//ZNF597//ZNF558//ZNF320//KDM2A//ACAD8//TAF5L//ZNF544//APEX1//ZNF699//AFF3//NFYC//YBX2//ATRX//COMMD8//PRKAA2//ARNTL2//REXO4//RORA//TAF11//BTF3//ZNF37A//ZNF75D//CSDE1//ZNF329//SAP30L//LZTR1//ZNF251//FSTL3//USP16//ZNF273//SUB1//WDHD1//FOXP4//CKS2//CERS6//GABPB1//ALX3//NPM1//QRICH1//THAP10//RFX4//ZNF704//HIF3A//SP4//SLTM//CREB5//DDX6//EIF4E//EIF5//NCBP2//OGFOD1//NF2//YWHAG//PSMA8//PSMA1//PSMC6//MUL1//BAK1//XBP1//TRAF4//G3BP2//CD40LG//TRIM71//SERPINB3//SERPINB4//USP51//WIZ//TRPC5//TIMP2//LARP4B//UPF3A//FBXW11//EBF3//CRLF3//RNF4//NCOA4//FUBP3//PIAS2//CHURC1//HAND2//ZNF488//PRAMEF19//MEX3B//TMEM100//CTDSPL2//NPPC//CALCR//PAX7//MOB1B//RAP2C//RASSF2//RIPK3//OSBPL8//MTM1//CRTC1//PDE1B//NECAB3//NECAB1//DHCR24//TRIAP1//CDK12//SCD//CCNC//TFRC//ASPH//E2F5//HOXA9//PBX3//PLAGL1//ALX4//YES1//MIXL1//GRIK2//C14ORF39//NOVA2//DCUN1D5//GTPBP1//QKI//PCK1//CUL2//EIF3A//INPP5B//WDR48//NMD3//RAD52// |
| GO:0017001 | antibiotic catabolic process | Biological process | 4 | 48 | 393 | 17653 | 3.74321458863444 | 0.0217987272308821 | 0.771857551112333 | 1.66156886294316 | 0.0101781170483461 | ALDH1B1//PCK1//GPX3//PRDX2// |
| GO:0042982 | amyloid precursor protein metabolic process | Biological process | 4 | 48 | 393 | 17653 | 3.74321458863444 | 0.0217987272308821 | 0.771857551112333 | 1.66156886294316 | 0.0101781170483461 | NECAB3//NECAB1//DHCR24//FKBP1A// |
| GO:0019725 | cellular homeostasis | Biological process | 30 | 916 | 393 | 17653 | 1.47113237107904 | 0.0225743803135527 | 0.774077795335659 | 1.6463841626135 | 0.0763358778625954 | APEX1//PRDX2//SLC24A2//GRIK2//ATP7A//TMEM199//TFRC//NCOA4//SLC30A5//ATP6V0E1//CCR5//F2R//IL2//TRPC5//C5AR1//BAK1//TRPC3//SLC41A1//XBP1//OXCT1//VSNL1//MTM1//FKBP1A//SLC9C2//SLC9A2//F2RL2//SLC30A2//GCLM//PCK1//CALCR// |
| GO:1901576 | organic substance biosynthetic process | Biological process | 168 | 6665 | 393 | 17653 | 1.1322311493904 | 0.0226922765465923 | 0.774077795335659 | 1.64412193240827 | 0.427480916030534 | KAT7//MCM10//DHFR//GFI1//KLF2//ZHX1//IRX2//DR1//CUX2//NR6A1//SMAD5//MITF//DLL4//H2AFY2//PURB//RARB//SMO//TRPS1//UBE2I//NCOA1//EIF3A//RPL7L1//SUB1//NCBP2//EBF3//TAF5L//NFYC//NPM1//PBX3//YBX2//PLAGL1//RFX4//ALX4//HIF3A//TAF11//BTF3//XBP1//MIXL1//FUBP3//HAND2//CREB5//SNRPF//SRSF11//ALDOB//PCK1//NPPC//PDE1B//CMPK1//WDHD1//CDK2AP1//FSTL3//PCGF3//USP16//ZNF273//FOXP4//ZNF488//ZNF597//ZNF558//ZNF320//E2F5//KDM2A//CRTC1//GABPB1//ALX3//ACAD8//ZNF544//HOXA9//APEX1//ZNF699//AFF3//PAX7//ATRX//COMMD8//PRKAA2//ARNTL2//RNF4//SP4//ZNF37A//ZNF75D//ZNF329//SAP30L//SLTM//ZC3H12A//NCOA4//PIAS2//ZNF251//CHURC1//REXO4//RORA//CSDE1//LZTR1//CKS2//CERS6//QRICH1//THAP10//ZNF704//CCNC//CDK12//MRPS7//COA1//EIF1AX//EIF4E//EIF5//DDX3Y//GTPBP1//DDX6//OGFOD1//NUS1//XPNPEP2//VNN1//ABCD3//SCD//ETNK2//DGKA//AGPAT5//AGPAT4//PIP5K1C//MTM1//PIP5K1A//SYNJ1//CYP19A1//DHCR24//IDI2//GCLM//CPOX//G3BP2//NF2//RFK//EREG//GALNT14//WIZ//LARP4B//UPF3A//THBS1//F2R//FBXW11//CRLF3//PRAMEF19//TRIM71//MEX3B//KERA//LMAN1//ZDHHC17//MOGAT3//ENOPH1//CANT1//TMEM100//CTDSPL2//NOD1//QKI//C5AR1//NECAB3//NECAB1//TIMP2//PSMC6//IL2//TRIAP1//YES1//C14ORF39//RIPK3//CD40LG//ATP7A//PSMA8//PSMA1//CUL2//RPE65//NMD3//RAP2C// |
| GO:0055065 | metal ion homeostasis | Biological process | 22 | 624 | 393 | 17653 | 1.58366771057611 | 0.0232273935091666 | 0.774077795335659 | 1.63399952236064 | 0.0559796437659033 | SLC24A2//GRIK2//ATP7A//TMEM199//TFRC//NCOA4//SLC30A5//CCR5//F2R//IL2//TRPC5//C5AR1//BAK1//TRPC3//SLC41A1//FKBP1A//F2RL2//STEAP2//B2M//SFXN1//SLC30A2//CALCR// |
| GO:0035335 | peptidyl-tyrosine dephosphorylation | Biological process | 6 | 99 | 393 | 17653 | 2.72233788264323 | 0.0232466001112202 | 0.774077795335659 | 1.63364055507107 | 0.0152671755725191 | MTM1//PTPN7//PTPRG//PTP4A2//DUSP16//CDC14A// |
| GO:0002521 | leukocyte differentiation | Biological process | 18 | 483 | 393 | 17653 | 1.67398416386136 | 0.0235740377850613 | 0.774077795335659 | 1.62756602470586 | 0.0458015267175573 | CMTM7//BAK1//CD40LG//IL2//DLL4//PDE1B//MITF//TFRC//RIPK3//B2M//VNN1//FAM20C//ATP7A//CD1D//XBP1//RASSF2//FSTL3//RORA// |
| GO:0018130 | heterocycle biosynthetic process | Biological process | 119 | 4550 | 393 | 17653 | 1.17479350166373 | 0.0237388927403288 | 0.774077795335659 | 1.6245395418254 | 0.302798982188295 | DHFR//GFI1//KLF2//ZHX1//IRX2//DR1//CUX2//NR6A1//SMAD5//MITF//DLL4//H2AFY2//PURB//RARB//SMO//TRPS1//UBE2I//NCOA1//SUB1//NCBP2//EBF3//TAF5L//NFYC//NPM1//PBX3//YBX2//PLAGL1//RFX4//ALX4//HIF3A//TAF11//BTF3//XBP1//MIXL1//FUBP3//HAND2//CREB5//SNRPF//SRSF11//ALDOB//NPPC//PDE1B//CMPK1//FSTL3//PCGF3//USP16//ZNF273//KAT7//FOXP4//ZNF488//ZNF597//ZNF558//ZNF320//E2F5//KDM2A//CRTC1//GABPB1//ALX3//ACAD8//ZNF544//HOXA9//APEX1//ZNF699//AFF3//PAX7//ATRX//COMMD8//PRKAA2//ARNTL2//RNF4//SP4//ZNF37A//ZNF75D//ZNF329//SAP30L//SLTM//ZC3H12A//NCOA4//PIAS2//ZNF251//CHURC1//REXO4//RORA//CSDE1//LZTR1//WDHD1//CKS2//CERS6//QRICH1//THAP10//ZNF704//CCNC//CDK12//CPOX//G3BP2//RFK//EREG//F2R//FBXW11//CRLF3//PRAMEF19//TMEM100//CTDSPL2//NOD1//C5AR1//TIMP2//PSMC6//IL2//TRIAP1//YES1//C14ORF39//RIPK3//CD40LG//PCK1//PSMA8//PSMA1//CUL2//NMD3//RAP2C// |
| GO:0008299 | isoprenoid biosynthetic process | Biological process | 3 | 28 | 393 | 17653 | 4.81270447110142 | 0.0237586002932751 | 0.774077795335659 | 1.62417914882607 | 0.00763358778625954 | IDI2//NUS1//RPE65// |
| GO:0048265 | response to pain | Biological process | 3 | 28 | 393 | 17653 | 4.81270447110142 | 0.0237586002932751 | 0.774077795335659 | 1.62417914882607 | 0.00763358778625954 | PIRT//VWA1//THBS1// |
| GO:0019883 | antigen processing and presentation of endogenous antigen | Biological process | 2 | 11 | 393 | 17653 | 8.16701364792968 | 0.0238072129868131 | 0.774077795335659 | 1.62329144270065 | 0.00508905852417303 | B2M//CD1D// |
| GO:0033235 | positive regulation of protein sumoylation | Biological process | 2 | 11 | 393 | 17653 | 8.16701364792968 | 0.0238072129868131 | 0.774077795335659 | 1.62329144270065 | 0.00508905852417303 | UBE2I//MUL1// |
| GO:0042983 | amyloid precursor protein biosynthetic process | Biological process | 2 | 11 | 393 | 17653 | 8.16701364792968 | 0.0238072129868131 | 0.774077795335659 | 1.62329144270065 | 0.00508905852417303 | NECAB3//NECAB1// |
| GO:0042984 | regulation of amyloid precursor protein biosynthetic process | Biological process | 2 | 11 | 393 | 17653 | 8.16701364792968 | 0.0238072129868131 | 0.774077795335659 | 1.62329144270065 | 0.00508905852417303 | NECAB3//NECAB1// |
| GO:0044650 | adhesion of symbiont to host cell | Biological process | 2 | 11 | 393 | 17653 | 8.16701364792968 | 0.0238072129868131 | 0.774077795335659 | 1.62329144270065 | 0.00508905852417303 | CLEC4M//CD209// |
| GO:0046007 | negative regulation of activated T cell proliferation | Biological process | 2 | 11 | 393 | 17653 | 8.16701364792968 | 0.0238072129868131 | 0.774077795335659 | 1.62329144270065 | 0.00508905852417303 | BTN2A2//CD274// |
| GO:0051024 | positive regulation of immunoglobulin secretion | Biological process | 2 | 11 | 393 | 17653 | 8.16701364792968 | 0.0238072129868131 | 0.774077795335659 | 1.62329144270065 | 0.00508905852417303 | IL2//XBP1// |
| GO:0061307 | cardiac neural crest cell differentiation involved in heart development | Biological process | 2 | 11 | 393 | 17653 | 8.16701364792968 | 0.0238072129868131 | 0.774077795335659 | 1.62329144270065 | 0.00508905852417303 | HAND2//SEMA3C// |
| GO:0061308 | cardiac neural crest cell development involved in heart development | Biological process | 2 | 11 | 393 | 17653 | 8.16701364792968 | 0.0238072129868131 | 0.774077795335659 | 1.62329144270065 | 0.00508905852417303 | HAND2//SEMA3C// |
| GO:0035556 | intracellular signal transduction | Biological process | 79 | 2871 | 393 | 17653 | 1.23600398120009 | 0.024110197566399 | 0.780178306897399 | 1.61779923086496 | 0.201017811704835 | CCR5//PSMA8//EREG//IL2//TAOK3//PSMA1//PSMC6//STK24//MAP3K8//F2R//NOD1//THBS1//C5AR1//DUSP16//NPM1//TRIAP1//PLAGL1//CALCR//SMO//RIPK3//TRAF4//MUL1//CD40LG//RAB6C//DHCR24//G3BP2//BAK1//PIP5K1C//PIP5K1A//ATRX//KAT7//GFRAL//MTM1//PRKAA2//RAB36//RAP2C//TIMP2//FAS//DYNLT1//F2RL2//MOB1B//NF2//FBXW11//AEN//CANT1//FKBP1A//ZDHHC17//UBE2I//MAPKBP1//RORA//ZC3H12A//CDK12//TMEM100//SERPINB3//PEX5L//GRIK2//RASSF2//CRLF3//PIRT//INPP5B//ARHGAP28//OSBPL8//OTUD3//XBP1//EIF3A//HAND2//PPP1R13B//CUL2//TAF11//VNN1//RNF152//CLEC4M//ASB15//DGKA//CD209//CAMK1G//RGS7//TUFT1//CCDC68// |
| GO:0070498 | interleukin-1-mediated signaling pathway | Biological process | 6 | 100 | 393 | 17653 | 2.69511450381679 | 0.0242840243702844 | 0.78046421812289 | 1.61467933996172 | 0.0152671755725191 | NOD1//MAP3K8//PSMA8//FBXW11//PSMA1//PSMC6// |
| GO:0016126 | sterol biosynthetic process | Biological process | 5 | 74 | 393 | 17653 | 3.03503885564954 | 0.0245547622410304 | 0.78046421812289 | 1.60986426670071 | 0.0127226463104326 | DHCR24//PRKAA2//IDI2//NFYC//SCD// |
| GO:0098659 | inorganic cation import across plasma membrane | Biological process | 4 | 50 | 393 | 17653 | 3.59348600508906 | 0.0249291835678806 | 0.78046421812289 | 1.60329194443126 | 0.0101781170483461 | STEAP2//SLC9C2//SLC9A2//CALCR// |
| GO:0099587 | inorganic ion import across plasma membrane | Biological process | 4 | 50 | 393 | 17653 | 3.59348600508906 | 0.0249291835678806 | 0.78046421812289 | 1.60329194443126 | 0.0101781170483461 | STEAP2//SLC9C2//SLC9A2//CALCR// |
| GO:0019438 | aromatic compound biosynthetic process | Biological process | 119 | 4558 | 393 | 17653 | 1.17273155607064 | 0.0249478139411525 | 0.78046421812289 | 1.60296750354315 | 0.302798982188295 | DHFR//GFI1//KLF2//ZHX1//IRX2//DR1//CUX2//NR6A1//SMAD5//MITF//DLL4//H2AFY2//PURB//RARB//SMO//TRPS1//UBE2I//NCOA1//SUB1//NCBP2//EBF3//TAF5L//NFYC//NPM1//PBX3//YBX2//PLAGL1//RFX4//ALX4//HIF3A//TAF11//BTF3//XBP1//MIXL1//FUBP3//HAND2//CREB5//SNRPF//SRSF11//ALDOB//NPPC//PDE1B//CMPK1//FSTL3//PCGF3//USP16//ZNF273//KAT7//FOXP4//ZNF488//ZNF597//ZNF558//ZNF320//E2F5//KDM2A//CRTC1//GABPB1//ALX3//ACAD8//ZNF544//HOXA9//APEX1//ZNF699//AFF3//PAX7//ATRX//COMMD8//PRKAA2//ARNTL2//RNF4//SP4//ZNF37A//ZNF75D//ZNF329//SAP30L//SLTM//ZC3H12A//NCOA4//PIAS2//ZNF251//CHURC1//REXO4//RORA//CSDE1//LZTR1//WDHD1//CKS2//CERS6//QRICH1//THAP10//ZNF704//CCNC//CDK12//CPOX//G3BP2//EREG//RFK//F2R//FBXW11//CRLF3//PRAMEF19//TMEM100//CTDSPL2//NOD1//C5AR1//TIMP2//PSMC6//IL2//TRIAP1//YES1//C14ORF39//RIPK3//CD40LG//PCK1//PSMA8//PSMA1//CUL2//NMD3//RAP2C// |
| GO:0030003 | cellular cation homeostasis | Biological process | 22 | 631 | 393 | 17653 | 1.56609928906417 | 0.0259075441819415 | 0.78046421812289 | 1.58657375254021 | 0.0559796437659033 | SLC24A2//GRIK2//ATP7A//TMEM199//TFRC//NCOA4//SLC30A5//ATP6V0E1//CCR5//F2R//IL2//TRPC5//C5AR1//BAK1//TRPC3//SLC41A1//FKBP1A//SLC9C2//SLC9A2//F2RL2//SLC30A2//CALCR// |
| GO:0007205 | protein kinase C-activating G-protein coupled receptor signaling pathway | Biological process | 3 | 29 | 393 | 17653 | 4.64674914451171 | 0.0260732361214459 | 0.78046421812289 | 1.58380506233131 | 0.00763358778625954 | DGKA//F2R//IL2// |
| GO:2000144 | positive regulation of DNA-templated transcription, initiation | Biological process | 3 | 29 | 393 | 17653 | 4.64674914451171 | 0.0260732361214459 | 0.78046421812289 | 1.58380506233131 | 0.00763358778625954 | PSMC6//SUB1//MITF// |
| GO:0006402 | mRNA catabolic process | Biological process | 14 | 351 | 393 | 17653 | 1.79162407661135 | 0.0261618967363803 | 0.78046421812289 | 1.58233077287521 | 0.0356234096692112 | NCBP2//UPF3A//EIF4E//ZC3H12A//PSMA8//APEX1//PSMA1//PSMC6//DDX6//GTPBP1//TRIM71//QKI//CSDE1//NPM1// |
| GO:0042493 | response to drug | Biological process | 31 | 966 | 393 | 17653 | 1.44148636332506 | 0.026403334736133 | 0.78046421812289 | 1.57834121835593 | 0.0788804071246819 | PDE1B//FKBP1A//DHFR//GJB2//THBS1//TRPC3//PRKAA2//TFRC//BAK1//STK24//IL2//NPPC//OXCT1//BCHE//KLF2//APEX1//NOD1//EIF4E//PCK1//KAT7//ATRX//GCLM//ZC3H12A//KIF1B//NCOA1//SEMA3C//LTA//B2M//ABCD3//TIMP2//RAB6C// |
| GO:0002504 | antigen processing and presentation of peptide or polysaccharide antigen via MHC class II | Biological process | 6 | 102 | 393 | 17653 | 2.64226912138901 | 0.0264507715279387 | 0.78046421812289 | 1.57756165574828 | 0.0152671755725191 | THBS1//IFI30//CLTA//DYNC1LI2//HLA-DPB1//CTSF// |
| GO:0032609 | interferon-gamma production | Biological process | 6 | 102 | 393 | 17653 | 2.64226912138901 | 0.0264507715279387 | 0.78046421812289 | 1.57756165574828 | 0.0152671755725191 | RIPK3//CD274//HLA-DPB1//IL2//LTA//ZC3H12A// |
| GO:0071887 | leukocyte apoptotic process | Biological process | 6 | 102 | 393 | 17653 | 2.64226912138901 | 0.0264507715279387 | 0.78046421812289 | 1.57756165574828 | 0.0152671755725191 | BAK1//IL2//CD3G//RIPK3//CD274//CCR5// |
| GO:0044271 | cellular nitrogen compound biosynthetic process | Biological process | 137 | 5342 | 393 | 17653 | 1.15197393929521 | 0.026572850272413 | 0.78046421812289 | 1.57556185954566 | 0.348600508905852 | DHFR//GFI1//KLF2//ZHX1//IRX2//DR1//CUX2//NR6A1//SMAD5//MITF//DLL4//H2AFY2//PURB//RARB//SMO//TRPS1//UBE2I//NCOA1//EIF3A//RPL7L1//SUB1//NCBP2//EBF3//TAF5L//NFYC//NPM1//PBX3//YBX2//PLAGL1//RFX4//ALX4//HIF3A//TAF11//BTF3//XBP1//MIXL1//FUBP3//HAND2//CREB5//SNRPF//SRSF11//ALDOB//NPPC//PDE1B//CMPK1//FSTL3//PCGF3//USP16//ZNF273//KAT7//FOXP4//ZNF488//ZNF597//ZNF558//ZNF320//E2F5//KDM2A//CRTC1//GABPB1//ALX3//ACAD8//ZNF544//HOXA9//APEX1//ZNF699//AFF3//PAX7//ATRX//COMMD8//PRKAA2//ARNTL2//RNF4//SP4//ZNF37A//ZNF75D//ZNF329//SAP30L//SLTM//ZC3H12A//NCOA4//PIAS2//ZNF251//CHURC1//REXO4//RORA//CSDE1//LZTR1//WDHD1//CKS2//CERS6//QRICH1//THAP10//ZNF704//CCNC//CDK12//MRPS7//COA1//EIF1AX//EIF4E//EIF5//DDX3Y//GTPBP1//DDX6//OGFOD1//GCLM//CPOX//G3BP2//RFK//EREG//LARP4B//UPF3A//THBS1//F2R//FBXW11//CRLF3//PRAMEF19//TRIM71//MEX3B//TMEM100//CTDSPL2//NOD1//C5AR1//TIMP2//PSMC6//IL2//TRIAP1//YES1//C14ORF39//RIPK3//CD40LG//QKI//PCK1//PSMA8//PSMA1//CUL2//NMD3//RAP2C// |
| GO:0010033 | response to organic substance | Biological process | 87 | 3218 | 393 | 17653 | 1.21439280004175 | 0.026863307760116 | 0.78046421812289 | 1.57084051238512 | 0.221374045801527 | PDE1B//NCOA1//B2M//THBS1//GPX3//BAK1//XBP1//FKBP1A//SMAD5//RPE65//ATP6V0E1//TRIM71//NCBP2//DHCR24//STEAP2//OXCT1//TIMP2//RARB//ABCD3//CCR5//EREG//IL2//RORA//STX1A//ATP7A//TSN//PSMC6//TMEM100//FSTL3//CTDSPL2//NR6A1//RNF4//NCOA4//PIAS2//DHFR//GJB2//C5AR1//F2R//FAS//LTA//PCK1//YWHAG//UFM1//TRPC3//PSMA8//PSMA1//CD40LG//CD274//AFF3//LTBP1//GCLM//VSNL1//DLL4//YES1//NUS1//FAM20C//BCHE//NPPC//OSBPL8//CALCR//CPOX//IFI30//HLA-DPB1//MUL1//NPM1//GFI1//NOD1//MAP3K8//FBXW11//UBE2W//ZC3H12A//CUX2//SYP//APEX1//PTPN7//KLF2//PRKAA2//SMO//EIF4E//KAT7//ATRX//KIF1B//DYNC1LI2//TFRC//LAPTM5//ADAM23//LRAT// |
| GO:0043488 | regulation of mRNA stability | Biological process | 8 | 160 | 393 | 17653 | 2.24592875318066 | 0.0270139362072045 | 0.78046421812289 | 1.56841213004733 | 0.0203562340966921 | TRIM71//ZC3H12A//QKI//NPM1//PSMA8//APEX1//PSMA1//PSMC6// |
| GO:0046661 | male sex differentiation | Biological process | 8 | 160 | 393 | 17653 | 2.24592875318066 | 0.0270139362072045 | 0.78046421812289 | 1.56841213004733 | 0.0203562340966921 | SMAD5//FSTL3//HOXA9//CSDE1//NCOA4//DHCR24//ATRX//WDR48// |
| GO:0034599 | cellular response to oxidative stress | Biological process | 12 | 286 | 393 | 17653 | 1.88469545721454 | 0.0270586655259258 | 0.78046421812289 | 1.56769362566218 | 0.0305343511450382 | STK24//ATP7A//PRDX2//KLF2//APEX1//VNN1//DHFR//GJB2//GPX3//RAD52//PYROXD1//ZC3H12A// |
| GO:0071453 | cellular response to oxygen levels | Biological process | 10 | 222 | 393 | 17653 | 2.0233592370997 | 0.0273613563841804 | 0.78046421812289 | 1.56286237714331 | 0.0254452926208651 | TMEM199//PSMA8//PSMA1//PSMC6//HIF3A//CUL2//FAS//PCK1//ATP7A//RORA// |
| GO:0099536 | synaptic signaling | Biological process | 23 | 671 | 393 | 17653 | 1.5396829008392 | 0.0273754656897642 | 0.78046421812289 | 1.56263848421485 | 0.0585241730279898 | STX1A//GRIK2//HTR1F//KCNC4//KCNQ3//PMP22//KIF1B//VDAC1//PIP5K1C//SYT16//SNAP47//SYNJ1//FGF14//YWHAG//SYP//SHISA6//SHISA9//BCHE//SLC24A2//KPNA1//F2R//CRTC1//CUX2// |
| GO:0022407 | regulation of cell-cell adhesion | Biological process | 15 | 388 | 393 | 17653 | 1.73654285039742 | 0.0278223032460961 | 0.78046421812289 | 1.55560692015041 | 0.0381679389312977 | NF2//FSTL3//MAP3K8//CD274//HLA-DPB1//YES1//CD3G//CD40LG//VNN1//CD209//IL2//TFRC//CD1D//XBP1//BTN2A2// |
| GO:0051960 | regulation of nervous system development | Biological process | 27 | 820 | 393 | 17653 | 1.47902625209458 | 0.0278876096083659 | 0.78046421812289 | 1.55458870977077 | 0.0687022900763359 | SMO//ZNF804A//DYNLT1//GFI1//PMP22//B2M//PTPRG//NF2//RFX4//RARB//YWHAG//DDX6//EIF4E//TIMP2//NCOA1//TRPC5//STK24//WDR1//ZNF488//SEMA3C//DLL4//CUX2//LTA//IL2//CRTC1//TUBB2B//MUL1// |
| GO:1902532 | negative regulation of intracellular signal transduction | Biological process | 19 | 528 | 393 | 17653 | 1.61638811781942 | 0.0279181329876429 | 0.78046421812289 | 1.55411362832236 | 0.0483460559796438 | DUSP16//THBS1//MTM1//PRKAA2//NF2//MAPKBP1//RORA//ZC3H12A//SERPINB3//TAOK3//TIMP2//OTUD3//MUL1//EIF3A//RASSF2//TRIAP1//VNN1//XBP1//RNF152// |
| GO:0043122 | regulation of I-kappaB kinase/NF-kappaB signaling | Biological process | 10 | 223 | 393 | 17653 | 2.01428587729207 | 0.0281028565419601 | 0.78046421812289 | 1.55124953356971 | 0.0254452926208651 | NOD1//CANT1//F2R//FKBP1A//ZDHHC17//UBE2I//MUL1//MAPKBP1//RORA//ZC3H12A// |
| GO:0009886 | post-embryonic animal morphogenesis | Biological process | 2 | 12 | 393 | 17653 | 7.48642917726887 | 0.0281538015777152 | 0.78046421812289 | 1.55046295453368 | 0.00508905852417303 | ATRX//BAK1// |
| GO:0010759 | positive regulation of macrophage chemotaxis | Biological process | 2 | 12 | 393 | 17653 | 7.48642917726887 | 0.0281538015777152 | 0.78046421812289 | 1.55046295453368 | 0.00508905852417303 | THBS1//C5AR1// |
| GO:0032119 | sequestering of zinc ion | Biological process | 2 | 12 | 393 | 17653 | 7.48642917726887 | 0.0281538015777152 | 0.78046421812289 | 1.55046295453368 | 0.00508905852417303 | SLC30A5//SLC30A2// |
| GO:0061042 | vascular wound healing | Biological process | 2 | 12 | 393 | 17653 | 7.48642917726887 | 0.0281538015777152 | 0.78046421812289 | 1.55046295453368 | 0.00508905852417303 | XBP1//MCAM// |
| GO:0070493 | thrombin-activated receptor signaling pathway | Biological process | 2 | 12 | 393 | 17653 | 7.48642917726887 | 0.0281538015777152 | 0.78046421812289 | 1.55046295453368 | 0.00508905852417303 | F2R//F2RL2// |
| GO:0072520 | seminiferous tubule development | Biological process | 2 | 12 | 393 | 17653 | 7.48642917726887 | 0.0281538015777152 | 0.78046421812289 | 1.55046295453368 | 0.00508905852417303 | ATRX//WDR48// |
| GO:0090085 | regulation of protein deubiquitination | Biological process | 2 | 12 | 393 | 17653 | 7.48642917726887 | 0.0281538015777152 | 0.78046421812289 | 1.55046295453368 | 0.00508905852417303 | WDR48//ZC3H12A// |
| GO:0010524 | positive regulation of calcium ion transport into cytosol | Biological process | 4 | 52 | 393 | 17653 | 3.45527500489333 | 0.0283171775521443 | 0.78046421812289 | 1.54795003610286 | 0.0101781170483461 | F2R//CALCR//BAK1//TRPC3// |
| GO:0036465 | synaptic vesicle recycling | Biological process | 4 | 52 | 393 | 17653 | 3.45527500489333 | 0.0283171775521443 | 0.78046421812289 | 1.54795003610286 | 0.0101781170483461 | SYNJ1//PIP5K1C//SYP//FGF14// |
| GO:0048168 | regulation of neuronal synaptic plasticity | Biological process | 4 | 52 | 393 | 17653 | 3.45527500489333 | 0.0283171775521443 | 0.78046421812289 | 1.54795003610286 | 0.0101781170483461 | GRIK2//SYP//SHISA6//SHISA9// |
| GO:0007006 | mitochondrial membrane organization | Biological process | 7 | 132 | 393 | 17653 | 2.38204564731282 | 0.0283652326288576 | 0.78046421812289 | 1.54721365035012 | 0.0178117048346056 | COX18//DYNLT1//TIMM9//BAK1//PPP1R13B//YWHAG//MUL1// |
| GO:0050671 | positive regulation of lymphocyte proliferation | Biological process | 7 | 132 | 393 | 17653 | 2.38204564731282 | 0.0283652326288576 | 0.78046421812289 | 1.54721365035012 | 0.0178117048346056 | IL2//TFRC//CD274//CD209//HLA-DPB1//CD1D//CD40LG// |
| GO:0000303 | response to superoxide | Biological process | 3 | 30 | 393 | 17653 | 4.49185750636132 | 0.0285043119734369 | 0.78046421812289 | 1.54508943737327 | 0.00763358778625954 | ATP7A//PRDX2//DHFR// |
| GO:0046685 | response to arsenic-containing substance | Biological process | 3 | 30 | 393 | 17653 | 4.49185750636132 | 0.0285043119734369 | 0.78046421812289 | 1.54508943737327 | 0.00763358778625954 | ZC3H12A//CPOX//RNF4// |
| GO:0007249 | I-kappaB kinase/NF-kappaB signaling | Biological process | 11 | 256 | 393 | 17653 | 1.93009502226463 | 0.0287992785297647 | 0.78220691042891 | 1.54061839190975 | 0.0279898218829517 | NOD1//CANT1//F2R//FKBP1A//ZDHHC17//UBE2I//MUL1//MAPKBP1//RORA//ZC3H12A//RIPK3// |
| GO:0072593 | reactive oxygen species metabolic process | Biological process | 11 | 256 | 393 | 17653 | 1.93009502226463 | 0.0287992785297647 | 0.78220691042891 | 1.54061839190975 | 0.0279898218829517 | RORA//RIPK3//THBS1//ZC3H12A//VDAC1//ATP7A//PRDX2//GPX3//KLF2//DHFR//RFK// |
| GO:1903311 | regulation of mRNA metabolic process | Biological process | 12 | 289 | 393 | 17653 | 1.86513114450989 | 0.0290053424338725 | 0.784652523521119 | 1.53752200293338 | 0.0305343511450382 | RBM7//NCBP2//PSMA8//APEX1//PSMA1//PSMC6//SLTM//ZC3H12A//GTPBP1//TRIM71//QKI//NPM1// |
| GO:0032946 | positive regulation of mononuclear cell proliferation | Biological process | 7 | 133 | 393 | 17653 | 2.36413552966385 | 0.0293923321608591 | 0.788810882555119 | 1.53176595307647 | 0.0178117048346056 | IL2//TFRC//CD274//CD209//HLA-DPB1//CD1D//CD40LG// |
| GO:0099504 | synaptic vesicle cycle | Biological process | 7 | 133 | 393 | 17653 | 2.36413552966385 | 0.0293923321608591 | 0.788810882555119 | 1.53176595307647 | 0.0178117048346056 | SYNJ1//STX1A//PIP5K1C//SYT16//SNAP47//SYP//FGF14// |
| GO:0042590 | antigen processing and presentation of exogenous peptide antigen via MHC class I | Biological process | 5 | 78 | 393 | 17653 | 2.8793958374111 | 0.0299743434522459 | 0.801250927934937 | 1.52325032076254 | 0.0127226463104326 | PSMA8//B2M//PSMA1//PSMC6//IFI30// |
| GO:0046068 | cGMP metabolic process | Biological process | 4 | 53 | 393 | 17653 | 3.39008113687647 | 0.0301087983944069 | 0.801676391895173 | 1.52130657630695 | 0.0101781170483461 | NPPC//THBS1//PDE1B//RORA// |
| GO:0042737 | drug catabolic process | Biological process | 7 | 134 | 393 | 17653 | 2.34649272720368 | 0.0304442579733005 | 0.807429477150711 | 1.5164946066654 | 0.0178117048346056 | ALDH1B1//XPNPEP1//PCK1//GPX3//PRDX2//SATL1//OXCT1// |
| GO:0044770 | cell cycle phase transition | Biological process | 21 | 606 | 393 | 17653 | 1.55658428438264 | 0.0307755477617235 | 0.813027459033344 | 1.51179420849326 | 0.0534351145038168 | TRIM71//EIF4E//CRLF3//MCM10//CUL2//DHFR//GFI1//FBXW11//YWHAG//CEP135//NPM1//TRIAP1//PLAGL1//CKS2//TAOK3//CDC14A//PSMA8//PSMA1//PSMC6//APEX1//TPRA1// |
| GO:0046902 | regulation of mitochondrial membrane permeability | Biological process | 5 | 79 | 393 | 17653 | 2.84294778883628 | 0.0314376168887796 | 0.815300473698556 | 1.50255038283872 | 0.0127226463104326 | DYNLT1//PPP1R13B//YWHAG//MUL1//BAK1// |
| GO:0061418 | regulation of transcription from RNA polymerase II promoter in response to hypoxia | Biological process | 5 | 79 | 393 | 17653 | 2.84294778883628 | 0.0314376168887796 | 0.815300473698556 | 1.50255038283872 | 0.0127226463104326 | PSMA8//PSMA1//PSMC6//HIF3A//CUL2// |
| GO:0044237 | cellular metabolic process | Biological process | 262 | 10957 | 393 | 17653 | 1.07407745429102 | 0.0315718473965289 | 0.815300473698556 | 1.500700005037 | 0.666666666666667 | KPNA1//KAT7//MCM10//DHFR//GFI1//KLF2//ZHX1//IRX2//DR1//CUX2//NR6A1//SMAD5//MITF//DLL4//H2AFY2//PURB//RARB//SMO//TRPS1//UBE2I//CCR5//PSMA8//EREG//IL2//TAOK3//PSMA1//PSMC6//STK24//NCBP2//UPF3A//MAP3K8//F2R//NOD1//THBS1//C5AR1//DUSP16//FBXW11//UBE2W//EIF4E//ZC3H12A//COPS7B//DBR1//RBM7//SNRPF//NOVA2//RNPC3//SRSF11//FKBP1A//NCOA1//RPL7L1//APEX1//WDR48//RAD52//EXOG//RPE65//LRAT//POP4//EIF3A//FAS//CDK2AP1//ATP7A//SUB1//EBF3//TAF5L//NFYC//NPM1//PBX3//YBX2//PLAGL1//RFX4//ALX4//HIF3A//TAF11//BTF3//XBP1//MIXL1//FUBP3//HAND2//CREB5//ASB15//WDSUB1//RNF152//RNF4//MUL1//CUL2//ALDH1B1//MOGAT3//PCK1//COX4I1//ALDOB//RPE//H6PD//CYP19A1//ASPH//ACPP//CMPK1//NPPC//PDE1B//WDHD1//USP51//ATRX//APEX2//KDM2A//TSN//FSTL3//PCGF3//USP16//ZNF273//FOXP4//ZNF488//ZNF597//ZNF558//ZNF320//E2F5//CRTC1//GABPB1//ALX3//ACAD8//ZNF544//HOXA9//ZNF699//AFF3//PAX7//COMMD8//PRKAA2//ARNTL2//SP4//ZNF37A//ZNF75D//ZNF329//SAP30L//SLTM//NCOA4//PIAS2//ZNF251//CHURC1//REXO4//RORA//CSDE1//LZTR1//CKS2//CERS6//QRICH1//THAP10//ZNF704//SENP3//CCNC//CDK12//QKI//MRPS7//COA1//EIF1AX//EIF5//DDX3Y//GTPBP1//DDX6//OGFOD1//RIPK3//YES1//SEC11A//SPEG//FAM20C//MEX3B//CDKL1//RASSF2//NF2//YWHAG//MTM1//CTDSPL2//PTPN7//PCMTD1//NUS1//XPNPEP2//VNN1//C2ORF66//CTSF//NGLY1//GCLM//MCCC2//CPQ//ABCD3//SCD//AGPAT5//ETNK2//PIP5K1A//DGKA//AGPAT4//PIP5K1C//SYNJ1//RFK//CPOX//NAT2//MARC2//LIX1L//BAK1//TRAF4//G3BP2//CD40LG//MAPKBP1//IDI2//TRIM71//SERPINB3//SERPINB4//WIZ//VDAC1//C14ORF39//TRPC5//XPNPEP1//TIMP2//LARP4B//EXOC8//ATP6V0E1//GALNT14//N4BP2//ENOPH1//CRLF3//PRAMEF19//TEX30//OTUD3//LYZL6//CAMK1G//KERA//LMAN1//ZDHHC17//CRAT//PRDX2//BCHE//DCUN1D5//CANT1//HYAL4//TMEM100//CALCR//BTN2A2//TFRC//MOB1B//RAP2C//OSBPL8//CLTA//SATL1//ASZ1//EXD1//PTPRG//PTP4A2//CDC14A//OXCT1//GPX3//NECAB3//NECAB1//DHCR24//TRIAP1//INPP5B//SUMF2//LTBP1//VWA1//B2M//GRIK2//SULT6B1//UFM1//AEN//PGBD5//NMD3//TMEM199// |
| GO:0071383 | cellular response to steroid hormone stimulus | Biological process | 11 | 260 | 393 | 17653 | 1.90040125269133 | 0.0317290498771888 | 0.815300473698556 | 1.49854293260403 | 0.0279898218829517 | NR6A1//RNF4//NCOA4//NCOA1//PIAS2//UFM1//RARB//RORA//EIF4E//GJB2//PCK1// |
| GO:0006873 | cellular ion homeostasis | Biological process | 22 | 645 | 393 | 17653 | 1.53210643627828 | 0.0319720685754119 | 0.815300473698556 | 1.495229264203 | 0.0559796437659033 | SLC24A2//GRIK2//ATP7A//TMEM199//TFRC//NCOA4//SLC30A5//ATP6V0E1//CCR5//F2R//IL2//TRPC5//C5AR1//BAK1//TRPC3//SLC41A1//FKBP1A//SLC9C2//SLC9A2//F2RL2//SLC30A2//CALCR// |
| GO:0007549 | dosage compensation | Biological process | 2 | 13 | 393 | 17653 | 6.91055000978665 | 0.0327904375049545 | 0.815300473698556 | 1.48425278873716 | 0.00508905852417303 | PCGF3//H2AFY2// |
| GO:0016024 | CDP-diacylglycerol biosynthetic process | Biological process | 2 | 13 | 393 | 17653 | 6.91055000978665 | 0.0327904375049545 | 0.815300473698556 | 1.48425278873716 | 0.00508905852417303 | AGPAT5//AGPAT4// |
| GO:0021692 | cerebellar Purkinje cell layer morphogenesis | Biological process | 2 | 13 | 393 | 17653 | 6.91055000978665 | 0.0327904375049545 | 0.815300473698556 | 1.48425278873716 | 0.00508905852417303 | ATP7A//RORA// |
| GO:0032486 | Rap protein signal transduction | Biological process | 2 | 13 | 393 | 17653 | 6.91055000978665 | 0.0327904375049545 | 0.815300473698556 | 1.48425278873716 | 0.00508905852417303 | TIMP2//RAP2C// |
| GO:0033262 | regulation of nuclear cell cycle DNA replication | Biological process | 2 | 13 | 393 | 17653 | 6.91055000978665 | 0.0327904375049545 | 0.815300473698556 | 1.48425278873716 | 0.00508905852417303 | ATRX//WIZ// |
| GO:0034063 | stress granule assembly | Biological process | 2 | 13 | 393 | 17653 | 6.91055000978665 | 0.0327904375049545 | 0.815300473698556 | 1.48425278873716 | 0.00508905852417303 | OGFOD1//G3BP2// |
| GO:0042415 | norepinephrine metabolic process | Biological process | 2 | 13 | 393 | 17653 | 6.91055000978665 | 0.0327904375049545 | 0.815300473698556 | 1.48425278873716 | 0.00508905852417303 | HAND2//ATP7A// |
| GO:0055089 | fatty acid homeostasis | Biological process | 2 | 13 | 393 | 17653 | 6.91055000978665 | 0.0327904375049545 | 0.815300473698556 | 1.48425278873716 | 0.00508905852417303 | PRKAA2//XBP1// |
| GO:0097202 | activation of cysteine-type endopeptidase activity | Biological process | 2 | 13 | 393 | 17653 | 6.91055000978665 | 0.0327904375049545 | 0.815300473698556 | 1.48425278873716 | 0.00508905852417303 | ASPH//BAK1// |
| GO:1901160 | primary amino compound metabolic process | Biological process | 2 | 13 | 393 | 17653 | 6.91055000978665 | 0.0327904375049545 | 0.815300473698556 | 1.48425278873716 | 0.00508905852417303 | PDE1B//ATP7A// |
| GO:1905523 | positive regulation of macrophage migration | Biological process | 2 | 13 | 393 | 17653 | 6.91055000978665 | 0.0327904375049545 | 0.815300473698556 | 1.48425278873716 | 0.00508905852417303 | THBS1//C5AR1// |
| GO:0009058 | biosynthetic process | Biological process | 168 | 6735 | 393 | 17653 | 1.12046334234403 | 0.0331851543425001 | 0.822092303363839 | 1.47905615813524 | 0.427480916030534 | KAT7//MCM10//DHFR//GFI1//KLF2//ZHX1//IRX2//DR1//CUX2//NR6A1//SMAD5//MITF//DLL4//H2AFY2//PURB//RARB//SMO//TRPS1//UBE2I//NCOA1//EIF3A//RPL7L1//SUB1//NCBP2//EBF3//TAF5L//NFYC//NPM1//PBX3//YBX2//PLAGL1//RFX4//ALX4//HIF3A//TAF11//BTF3//XBP1//MIXL1//FUBP3//HAND2//CREB5//SNRPF//SRSF11//ALDOB//PCK1//NPPC//PDE1B//CMPK1//WDHD1//CDK2AP1//FSTL3//PCGF3//USP16//ZNF273//FOXP4//ZNF488//ZNF597//ZNF558//ZNF320//E2F5//KDM2A//CRTC1//GABPB1//ALX3//ACAD8//ZNF544//HOXA9//APEX1//ZNF699//AFF3//PAX7//ATRX//COMMD8//PRKAA2//ARNTL2//RNF4//SP4//ZNF37A//ZNF75D//ZNF329//SAP30L//SLTM//ZC3H12A//NCOA4//PIAS2//ZNF251//CHURC1//REXO4//RORA//CSDE1//LZTR1//CKS2//CERS6//QRICH1//THAP10//ZNF704//CCNC//CDK12//MRPS7//COA1//EIF1AX//EIF4E//EIF5//DDX3Y//GTPBP1//DDX6//OGFOD1//NUS1//XPNPEP2//VNN1//ABCD3//SCD//ETNK2//DGKA//AGPAT5//AGPAT4//PIP5K1C//MTM1//PIP5K1A//SYNJ1//CYP19A1//DHCR24//IDI2//GCLM//CPOX//G3BP2//NF2//RFK//EREG//GALNT14//WIZ//LARP4B//UPF3A//THBS1//F2R//FBXW11//CRLF3//PRAMEF19//TRIM71//MEX3B//KERA//LMAN1//ZDHHC17//MOGAT3//ENOPH1//CANT1//TMEM100//CTDSPL2//NOD1//QKI//C5AR1//NECAB3//NECAB1//TIMP2//PSMC6//IL2//TRIAP1//YES1//C14ORF39//RIPK3//CD40LG//ATP7A//PSMA8//PSMA1//CUL2//RPE65//NMD3//RAP2C// |
| GO:0002714 | positive regulation of B cell mediated immunity | Biological process | 3 | 32 | 393 | 17653 | 4.21111641221374 | 0.0337138768534552 | 0.827402133606196 | 1.4721913038643 | 0.00763358778625954 | LTA//TFRC//IL2// |
| GO:0002891 | positive regulation of immunoglobulin mediated immune response | Biological process | 3 | 32 | 393 | 17653 | 4.21111641221374 | 0.0337138768534552 | 0.827402133606196 | 1.4721913038643 | 0.00763358778625954 | LTA//TFRC//IL2// |
| GO:0070228 | regulation of lymphocyte apoptotic process | Biological process | 4 | 55 | 393 | 17653 | 3.26680545917187 | 0.0338888645584676 | 0.827402133606196 | 1.46994298189301 | 0.0101781170483461 | IL2//RIPK3//CD274//CD3G// |
| GO:0097345 | mitochondrial outer membrane permeabilization | Biological process | 4 | 55 | 393 | 17653 | 3.26680545917187 | 0.0338888645584676 | 0.827402133606196 | 1.46994298189301 | 0.0101781170483461 | PPP1R13B//YWHAG//MUL1//BAK1// |
| GO:0071704 | organic substance metabolic process | Biological process | 268 | 11249 | 393 | 17653 | 1.07015540199559 | 0.0341636471868445 | 0.831110596851185 | 1.46643577180711 | 0.681933842239186 | KPNA1//KAT7//MCM10//DHFR//GFI1//KLF2//ZHX1//IRX2//DR1//CUX2//NR6A1//SMAD5//MITF//DLL4//H2AFY2//PURB//RARB//SMO//TRPS1//UBE2I//CCR5//PSMA8//EREG//IL2//TAOK3//PSMA1//PSMC6//STK24//NCBP2//UPF3A//MAP3K8//F2R//NOD1//THBS1//C5AR1//DUSP16//FBXW11//UBE2W//EIF4E//ZC3H12A//COPS7B//DBR1//RBM7//SNRPF//NOVA2//RNPC3//SRSF11//FKBP1A//NCOA1//RPL7L1//APEX1//WDR48//RAD52//EXOG//RPE65//LRAT//POP4//EIF3A//FAS//CDK2AP1//ATP7A//SUB1//EBF3//TAF5L//NFYC//NPM1//PBX3//YBX2//PLAGL1//RFX4//ALX4//HIF3A//TAF11//BTF3//XBP1//MIXL1//FUBP3//HAND2//CREB5//ASB15//WDSUB1//RNF152//RNF4//MUL1//CUL2//YDJC//ALDH1B1//HYAL4//RPE//ALDOB//PCK1//H6PD//MOGAT3//COX4I1//ACPP//CMPK1//NPPC//PDE1B//WDHD1//USP51//ATRX//APEX2//KDM2A//TSN//FSTL3//PCGF3//USP16//ZNF273//FOXP4//ZNF488//ZNF597//ZNF558//ZNF320//E2F5//CRTC1//GABPB1//ALX3//ACAD8//ZNF544//HOXA9//ZNF699//AFF3//PAX7//COMMD8//PRKAA2//ARNTL2//SP4//ZNF37A//ZNF75D//ZNF329//SAP30L//SLTM//NCOA4//PIAS2//ZNF251//CHURC1//REXO4//RORA//CSDE1//LZTR1//CKS2//CERS6//QRICH1//THAP10//ZNF704//SENP3//CCNC//CDK12//QKI//MRPS7//COA1//EIF1AX//EIF5//DDX3Y//GTPBP1//DDX6//OGFOD1//RIPK3//YES1//SEC11A//SPEG//FAM20C//MEX3B//CDKL1//RASSF2//NF2//YWHAG//MTM1//CTDSPL2//PTPN7//PCMTD1//ASPH//NUS1//XPNPEP2//VNN1//C2ORF66//CPQ//GZMK//NRIP3//PRSS22//ST14//XPNPEP1//CTSF//ADAM23//NGLY1//GCLM//MCCC2//BTN2A1//TPRA1//ABCD3//SCD//AGPAT5//ETNK2//PIP5K1A//DGKA//AGPAT4//PIP5K1C//SYNJ1//CYP19A1//DHCR24//IDI2//RFK//CPOX//BAK1//TRAF4//G3BP2//CD40LG//MAPKBP1//PGLYRP4//GALNT14//TRIM71//SERPINB3//SERPINB4//WIZ//KIF1B//CALCR//ZNF804A//C14ORF39//TRPC5//TIMP2//PLCXD3//LARP4B//EBPL//CRLF3//PRAMEF19//TEX30//OTUD3//LYZL6//CAMK1G//KERA//LMAN1//ZDHHC17//CRAT//ENOPH1//NECAB3//BCHE//DCUN1D5//CANT1//TMEM100//ASZ1//EXD1//TFRC//MOB1B//RAP2C//OSBPL8//CLTA//SATL1//PTPRG//PTP4A2//CDC14A//MARC2//OXCT1//NECAB1//TRIAP1//INPP5B//SUMF2//LTBP1//VWA1//B2M//GRIK2//SULT6B1//TSPAN5//UFM1//N4BP2//AEN//PGBD5//NMD3//TMEM199// |
| GO:0043487 | regulation of RNA stability | Biological process | 8 | 168 | 393 | 17653 | 2.13897976493396 | 0.0346517819568099 | 0.839964162630485 | 1.46027442702301 | 0.0203562340966921 | PSMA8//APEX1//PSMA1//PSMC6//TRIM71//ZC3H12A//QKI//NPM1// |
| GO:1905952 | regulation of lipid localization | Biological process | 7 | 138 | 393 | 17653 | 2.27847844525574 | 0.0349045007953441 | 0.843068353138972 | 1.45711856889889 | 0.0178117048346056 | THBS1//ZC3H12A//OSBPL8//LRAT//NUS1//CYP19A1//TRIAP1// |
| GO:0050852 | T cell receptor signaling pathway | Biological process | 9 | 200 | 393 | 17653 | 2.0213358778626 | 0.0354503755606551 | 0.848763772103175 | 1.45037915954844 | 0.0229007633587786 | BTN2A2//BTN2A1//PSMA8//FBXW11//HLA-DPB1//PSMA1//PSMC6//ZC3H12A//CD3G// |
| GO:0071229 | cellular response to acid chemical | Biological process | 9 | 200 | 393 | 17653 | 2.0213358778626 | 0.0354503755606551 | 0.848763772103175 | 1.45037915954844 | 0.0229007633587786 | ATP7A//XBP1//PCK1//RARB//YES1//PRKAA2//GCLM//KLF2//ZC3H12A// |
| GO:0046328 | regulation of JNK cascade | Biological process | 8 | 169 | 393 | 17653 | 2.12632307993435 | 0.0356994865352178 | 0.848763772103175 | 1.44733803028907 | 0.0203562340966921 | MUL1//CD40LG//TAOK3//SERPINB3//NOD1//TRAF4//RASSF2//GRIK2// |
| GO:0002637 | regulation of immunoglobulin production | Biological process | 4 | 56 | 393 | 17653 | 3.20846964740095 | 0.0358777672612123 | 0.848763772103175 | 1.44517459172344 | 0.0101781170483461 | XBP1//TFRC//IL2//CD40LG// |
| GO:0046824 | positive regulation of nucleocytoplasmic transport | Biological process | 4 | 56 | 393 | 17653 | 3.20846964740095 | 0.0358777672612123 | 0.848763772103175 | 1.44517459172344 | 0.0101781170483461 | SMO//ZC3H12A//CTDSPL2//NCBP2// |
| GO:0009267 | cellular response to starvation | Biological process | 7 | 139 | 393 | 17653 | 2.26208651399491 | 0.0360837046956969 | 0.848763772103175 | 1.44268888004635 | 0.0178117048346056 | RNF152//FAS//PRKAA2//XBP1//ZC3H12A//PCK1//STK24// |
| GO:0050670 | regulation of lymphocyte proliferation | Biological process | 9 | 201 | 393 | 17653 | 2.01127948046029 | 0.0364168857322579 | 0.848763772103175 | 1.43869719657309 | 0.0229007633587786 | IL2//TFRC//CD274//CD209//HLA-DPB1//CD1D//CD40LG//RIPK3//BTN2A2// |
| GO:0000305 | response to oxygen radical | Biological process | 3 | 33 | 393 | 17653 | 4.08350682396484 | 0.0364910030775764 | 0.848763772103175 | 1.43781419843122 | 0.00763358778625954 | ATP7A//PRDX2//DHFR// |
| GO:0035115 | embryonic forelimb morphogenesis | Biological process | 3 | 33 | 393 | 17653 | 4.08350682396484 | 0.0364910030775764 | 0.848763772103175 | 1.43781419843122 | 0.00763358778625954 | ALX3//HOXA9//ALX4// |
| GO:0071549 | cellular response to dexamethasone stimulus | Biological process | 3 | 33 | 393 | 17653 | 4.08350682396484 | 0.0364910030775764 | 0.848763772103175 | 1.43781419843122 | 0.00763358778625954 | EIF4E//GJB2//PCK1// |
| GO:0070665 | positive regulation of leukocyte proliferation | Biological process | 7 | 140 | 393 | 17653 | 2.24592875318066 | 0.0372889442472689 | 0.848763772103175 | 1.42841991253776 | 0.0178117048346056 | IL2//TFRC//CD274//CD209//HLA-DPB1//CD1D//CD40LG// |
| GO:0032944 | regulation of mononuclear cell proliferation | Biological process | 9 | 202 | 393 | 17653 | 2.0013226513491 | 0.0374013106708287 | 0.848763772103175 | 1.42711317835742 | 0.0229007633587786 | IL2//TFRC//CD274//CD209//HLA-DPB1//CD1D//CD40LG//RIPK3//BTN2A2// |
| GO:0000054 | ribosomal subunit export from nucleus | Biological process | 2 | 14 | 393 | 17653 | 6.41693929480189 | 0.0377021217945255 | 0.848763772103175 | 1.42363420794476 | 0.00508905852417303 | NPM1//NMD3// |
| GO:0033750 | ribosome localization | Biological process | 2 | 14 | 393 | 17653 | 6.41693929480189 | 0.0377021217945255 | 0.848763772103175 | 1.42363420794476 | 0.00508905852417303 | NPM1//NMD3// |
| GO:0036295 | cellular response to increased oxygen levels | Biological process | 2 | 14 | 393 | 17653 | 6.41693929480189 | 0.0377021217945255 | 0.848763772103175 | 1.42363420794476 | 0.00508905852417303 | FAS//TMEM199// |
| GO:0044406 | adhesion of symbiont to host | Biological process | 2 | 14 | 393 | 17653 | 6.41693929480189 | 0.0377021217945255 | 0.848763772103175 | 1.42363420794476 | 0.00508905852417303 | CLEC4M//CD209// |
| GO:0046341 | CDP-diacylglycerol metabolic process | Biological process | 2 | 14 | 393 | 17653 | 6.41693929480189 | 0.0377021217945255 | 0.848763772103175 | 1.42363420794476 | 0.00508905852417303 | AGPAT5//AGPAT4// |
| GO:0070071 | proton-transporting two-sector ATPase complex assembly | Biological process | 2 | 14 | 393 | 17653 | 6.41693929480189 | 0.0377021217945255 | 0.848763772103175 | 1.42363420794476 | 0.00508905852417303 | TMEM199//ALDOB// |
| GO:1900103 | positive regulation of endoplasmic reticulum unfolded protein response | Biological process | 2 | 14 | 393 | 17653 | 6.41693929480189 | 0.0377021217945255 | 0.848763772103175 | 1.42363420794476 | 0.00508905852417303 | BAK1//XBP1// |
| GO:2000105 | positive regulation of DNA-dependent DNA replication | Biological process | 2 | 14 | 393 | 17653 | 6.41693929480189 | 0.0377021217945255 | 0.848763772103175 | 1.42363420794476 | 0.00508905852417303 | ATRX//WIZ// |
| GO:0032675 | regulation of interleukin-6 production | Biological process | 6 | 111 | 393 | 17653 | 2.42803108451963 | 0.0377758236585917 | 0.848763772103175 | 1.42278605759167 | 0.0152671755725191 | KLF2//ZC3H12A//NOD1//EREG//F2R//XBP1// |
| GO:0048878 | chemical homeostasis | Biological process | 34 | 1114 | 393 | 17653 | 1.37094394269556 | 0.0385557456481573 | 0.862568265391248 | 1.41391089342747 | 0.0865139949109415 | SLC24A2//GRIK2//ATP7A//TMEM199//TFRC//NCOA4//SLC30A5//ATP6V0E1//CCR5//F2R//IL2//TRPC5//C5AR1//BAK1//TRPC3//SLC41A1//DYNLT1//XBP1//OXCT1//VSNL1//PCK1//PRKAA2//NUS1//FKBP1A//SLC9C2//SLC9A2//F2RL2//GCLM//STEAP2//B2M//SFXN1//SLC30A2//RORA//CALCR// |
| GO:0048246 | macrophage chemotaxis | Biological process | 3 | 34 | 393 | 17653 | 3.96340368208352 | 0.0393818642686222 | 0.862568265391248 | 1.40470372896957 | 0.00763358778625954 | THBS1//C5AR1//CYP19A1// |
| GO:0051930 | regulation of sensory perception of pain | Biological process | 3 | 34 | 393 | 17653 | 3.96340368208352 | 0.0393818642686222 | 0.862568265391248 | 1.40470372896957 | 0.00763358778625954 | F2R//ACPP//TMEM100// |
| GO:0051931 | regulation of sensory perception | Biological process | 3 | 34 | 393 | 17653 | 3.96340368208352 | 0.0393818642686222 | 0.862568265391248 | 1.40470372896957 | 0.00763358778625954 | F2R//ACPP//TMEM100// |
| GO:0072604 | interleukin-6 secretion | Biological process | 3 | 34 | 393 | 17653 | 3.96340368208352 | 0.0393818642686222 | 0.862568265391248 | 1.40470372896957 | 0.00763358778625954 | ZC3H12A//F2R//XBP1// |
| GO:2000106 | regulation of leukocyte apoptotic process | Biological process | 5 | 84 | 393 | 17653 | 2.67372470616745 | 0.0394195428143651 | 0.862568265391248 | 1.40428841695028 | 0.0127226463104326 | IL2//CD3G//RIPK3//CD274//CCR5// |
| GO:0008610 | lipid biosynthetic process | Biological process | 23 | 697 | 393 | 17653 | 1.48224853151091 | 0.039522081937943 | 0.862568265391248 | 1.40316018578621 | 0.0585241730279898 | NUS1//PRKAA2//ABCD3//XBP1//SCD//ETNK2//DGKA//AGPAT5//AGPAT4//PIP5K1C//MTM1//PIP5K1A//SYNJ1//CYP19A1//DHCR24//IDI2//GFI1//MOGAT3//CERS6//QKI//NFYC//PCK1//RPE65// |
| GO:0043687 | post-translational protein modification | Biological process | 16 | 442 | 393 | 17653 | 1.62601176700862 | 0.0395328698530908 | 0.862568265391248 | 1.40304165729576 | 0.0407124681933842 | XPNPEP2//VNN1//FSTL3//ASB15//PSMA8//FBXW11//SUMF2//LTBP1//PSMA1//FAM20C//PSMC6//HIF3A//COPS7B//VWA1//PTP4A2//CUL2// |
| GO:0055085 | transmembrane transport | Biological process | 45 | 1550 | 393 | 17653 | 1.30408766313716 | 0.0396063390424706 | 0.862568265391248 | 1.40223529915423 | 0.114503816793893 | VDAC1//PRKAA2//SLC30A5//BAK1//THBS1//CCR5//ATP7A//ATP6V0E1//CLTCL1//PEX5L//SLC24A2//GRIK2//ASPH//SFXN1//KCNC4//KCNQ3//CACNA2D4//SLC41A1//TIMM9//F2R//FKBP1A//WDR1//TRPC3//TRPC5//SLC30A2//SLC9C2//KCNS1//SLC9A2//LRRC8B//STEAP2//FGF14//COX4I1//ZDHHC17//CALCR//SHISA6//SHISA9//PIRT//OSBPL8//PSMA8//MFSD8//GJB2//SLC25A35//PSMA1//PSMC6//ABCD3// |
| GO:0006875 | cellular metal ion homeostasis | Biological process | 19 | 550 | 393 | 17653 | 1.55173259310664 | 0.0396656410670824 | 0.862568265391248 | 1.4015855223523 | 0.0483460559796438 | SLC24A2//GRIK2//ATP7A//TMEM199//TFRC//NCOA4//SLC30A5//CCR5//F2R//IL2//TRPC5//C5AR1//BAK1//TRPC3//SLC41A1//FKBP1A//F2RL2//SLC30A2//CALCR// |
| GO:0014823 | response to activity | Biological process | 4 | 58 | 393 | 17653 | 3.09783276300781 | 0.0400540336785046 | 0.862692451489575 | 1.39735374134991 | 0.0101781170483461 | PRKAA2//GCLM//OXCT1//PCK1// |
| GO:0032768 | regulation of monooxygenase activity | Biological process | 4 | 58 | 393 | 17653 | 3.09783276300781 | 0.0400540336785046 | 0.862692451489575 | 1.39735374134991 | 0.0101781170483461 | GFI1//NOD1//NUS1//DHFR// |
| GO:0075733 | intracellular transport of virus | Biological process | 4 | 58 | 393 | 17653 | 3.09783276300781 | 0.0400540336785046 | 0.862692451489575 | 1.39735374134991 | 0.0101781170483461 | DYNLT1//CLEC4M//CD209//KPNA1// |
| GO:0034220 | ion transmembrane transport | Biological process | 34 | 1119 | 393 | 17653 | 1.36481818781309 | 0.0406582843956799 | 0.871822578174872 | 1.39085095066622 | 0.0865139949109415 | VDAC1//PRKAA2//SLC30A5//BAK1//THBS1//CCR5//ATP7A//ATP6V0E1//KCNC4//KCNQ3//CACNA2D4//SLC41A1//SLC24A2//F2R//FKBP1A//WDR1//TRPC3//TRPC5//SLC30A2//SLC9C2//KCNS1//SLC9A2//LRRC8B//STEAP2//FGF14//COX4I1//ZDHHC17//CALCR//SHISA6//SHISA9//PIRT//GRIK2//ASPH//SFXN1// |
| GO:0035264 | multicellular organism growth | Biological process | 7 | 143 | 393 | 17653 | 2.1988113667503 | 0.0410626040015243 | 0.871822578174872 | 1.38655351313314 | 0.0178117048346056 | NPPC//SMO//KLF2//ATRX//ETNK2//WDR48//RARB// |
| GO:0034612 | response to tumor necrosis factor | Biological process | 12 | 305 | 393 | 17653 | 1.76728819922413 | 0.0411261699985904 | 0.871822578174872 | 1.3858817336206 | 0.0305343511450382 | PSMA8//FAS//LTA//PSMA1//PSMC6//CD40LG//KLF2//PCK1//RORA//THBS1//ZC3H12A//AFF3// |
| GO:0010035 | response to inorganic substance | Biological process | 18 | 517 | 393 | 17653 | 1.56389623045462 | 0.0417228616965736 | 0.871822578174872 | 1.37962591174099 | 0.0458015267175573 | CPOX//FKBP1A//ATP7A//SLC30A5//SLC30A2//PRDX2//B2M//THBS1//BAK1//STK24//TRPC3//KLF2//APEX1//FSTL3//SLC41A1//ATRX//ZC3H12A//DHFR// |
| GO:0045667 | regulation of osteoblast differentiation | Biological process | 6 | 114 | 393 | 17653 | 2.36413552966385 | 0.0421443866251857 | 0.871822578174872 | 1.37526026255889 | 0.0152671755725191 | HAND2//SMAD5//NPPC//FAM20C//PIAS2//RASSF2// |
| GO:0046916 | cellular transition metal ion homeostasis | Biological process | 6 | 114 | 393 | 17653 | 2.36413552966385 | 0.0421443866251857 | 0.871822578174872 | 1.37526026255889 | 0.0152671755725191 | ATP7A//TMEM199//TFRC//NCOA4//SLC30A5//SLC30A2// |
| GO:0001662 | behavioral fear response | Biological process | 3 | 35 | 393 | 17653 | 3.85016357688113 | 0.0423854151470382 | 0.871822578174872 | 1.37278355875688 | 0.00763358778625954 | EIF4E//GRIK2//VDAC1// |
| GO:0002209 | behavioral defense response | Biological process | 3 | 35 | 393 | 17653 | 3.85016357688113 | 0.0423854151470382 | 0.871822578174872 | 1.37278355875688 | 0.00763358778625954 | EIF4E//GRIK2//VDAC1// |
| GO:0051204 | protein insertion into mitochondrial membrane | Biological process | 3 | 35 | 393 | 17653 | 3.85016357688113 | 0.0423854151470382 | 0.871822578174872 | 1.37278355875688 | 0.00763358778625954 | COX18//PPP1R13B//YWHAG// |
| GO:1903523 | negative regulation of blood circulation | Biological process | 3 | 35 | 393 | 17653 | 3.85016357688113 | 0.0423854151470382 | 0.871822578174872 | 1.37278355875688 | 0.00763358778625954 | IL2//ZC3H12A//F2R// |
| GO:2000826 | regulation of heart morphogenesis | Biological process | 3 | 35 | 393 | 17653 | 3.85016357688113 | 0.0423854151470382 | 0.871822578174872 | 1.37278355875688 | 0.00763358778625954 | HAND2//SEMA3C//SMO// |
| GO:0007548 | sex differentiation | Biological process | 11 | 273 | 393 | 17653 | 1.80990595494412 | 0.0427077227440127 | 0.871822578174872 | 1.36949358535324 | 0.0279898218829517 | EREG//SMAD5//FSTL3//HOXA9//CSDE1//NCOA4//CYP19A1//DHCR24//ATRX//BAK1//WDR48// |
| GO:0033554 | cellular response to stress | Biological process | 55 | 1962 | 393 | 17653 | 1.25918533562626 | 0.0427736459342857 | 0.871822578174872 | 1.36882372983071 | 0.139949109414758 | COPS7B//WDR48//RAD52//WDHD1//USP51//APEX1//NPM1//TAOK3//ATRX//UBE2W//APEX2//KDM2A//USP16//MCM10//ZC3H12A//TRIAP1//PLAGL1//BAK1//XBP1//NOD1//MUL1//CD40LG//STK24//C14ORF39//ATP7A//PRDX2//PSMC6//KAT7//MAP3K8//GFRAL//DHFR//FAS//RNF152//GJB2//GPX3//PYROXD1//THBS1//UFM1//KLF2//PRKAA2//AEN//SERPINB3//GRIK2//TRAF4//RASSF2//PCK1//FBXW11//PSMA8//PSMA1//HIF3A//CUL2//UBE2I//RORA//HAND2//VNN1// |
| GO:0003417 | growth plate cartilage development | Biological process | 2 | 15 | 393 | 17653 | 5.9891433418151 | 0.0428743765829588 | 0.871822578174872 | 1.36780218178414 | 0.00508905852417303 | NPPC//RARB// |
| GO:0021924 | cell proliferation in external granule layer | Biological process | 2 | 15 | 393 | 17653 | 5.9891433418151 | 0.0428743765829588 | 0.871822578174872 | 1.36780218178414 | 0.00508905852417303 | RORA// |
| GO:0021930 | cerebellar granule cell precursor proliferation | Biological process | 2 | 15 | 393 | 17653 | 5.9891433418151 | 0.0428743765829588 | 0.871822578174872 | 1.36780218178414 | 0.00508905852417303 | RORA// |
| GO:0098719 | sodium ion import across plasma membrane | Biological process | 2 | 15 | 393 | 17653 | 5.9891433418151 | 0.0428743765829588 | 0.871822578174872 | 1.36780218178414 | 0.00508905852417303 | SLC9C2//SLC9A2// |
| GO:1990000 | amyloid fibril formation | Biological process | 2 | 15 | 393 | 17653 | 5.9891433418151 | 0.0428743765829588 | 0.871822578174872 | 1.36780218178414 | 0.00508905852417303 | RIPK3//FKBP1A// |
| GO:1901655 | cellular response to ketone | Biological process | 5 | 86 | 393 | 17653 | 2.61154506183798 | 0.0429272391737738 | 0.871822578174872 | 1.36726704187582 | 0.0127226463104326 | PRKAA2//KLF2//EIF4E//GJB2//PCK1// |
| GO:0034097 | response to cytokine | Biological process | 35 | 1164 | 393 | 17653 | 1.35064443919799 | 0.0432668654922303 | 0.876089255460939 | 1.36384456652388 | 0.089058524173028 | CCR5//EREG//FKBP1A//IL2//RORA//STX1A//PSMA8//FAS//LTA//PSMA1//PSMC6//CD40LG//AFF3//PDE1B//IFI30//HLA-DPB1//B2M//MUL1//GFI1//NOD1//MAP3K8//FBXW11//PCK1//PTPN7//KLF2//ZC3H12A//XBP1//THBS1//GCLM//TFRC//LAPTM5//ADAM23//LRAT//CD274//TIMP2// |
| GO:0007268 | chemical synaptic transmission | Biological process | 22 | 667 | 393 | 17653 | 1.48157219100373 | 0.0435827081898628 | 0.87723171276203 | 1.36068578658701 | 0.0559796437659033 | STX1A//KIF1B//VDAC1//PIP5K1C//SYT16//SNAP47//SYNJ1//GRIK2//KCNC4//FGF14//YWHAG//SYP//SHISA6//SHISA9//BCHE//SLC24A2//F2R//CRTC1//CUX2//HTR1F//KCNQ3//PMP22// |
| GO:0098916 | anterograde trans-synaptic signaling | Biological process | 22 | 667 | 393 | 17653 | 1.48157219100373 | 0.0435827081898628 | 0.87723171276203 | 1.36068578658701 | 0.0559796437659033 | STX1A//GRIK2//HTR1F//KCNC4//KCNQ3//PMP22//KIF1B//VDAC1//PIP5K1C//SYT16//SNAP47//SYNJ1//FGF14//YWHAG//SYP//SHISA6//SHISA9//BCHE//SLC24A2//F2R//CRTC1//CUX2// |
| GO:0001505 | regulation of neurotransmitter levels | Biological process | 12 | 308 | 393 | 17653 | 1.75007435312779 | 0.0437422785675881 | 0.877830949414238 | 1.35909859798892 | 0.0305343511450382 | STX1A//RORA//PIP5K1C//SYT16//SNAP47//SYNJ1//BCHE//PDE1B//ATP7A//ZC3H12A//KLF2//KCNC4// |
| GO:0090559 | regulation of membrane permeability | Biological process | 5 | 87 | 393 | 17653 | 2.58152730250651 | 0.044749279217896 | 0.893017477729563 | 1.34921395552598 | 0.0127226463104326 | DYNLT1//BAK1//PPP1R13B//YWHAG//MUL1// |
| GO:0042594 | response to starvation | Biological process | 8 | 177 | 393 | 17653 | 2.03021808197122 | 0.0448565154936398 | 0.893017477729563 | 1.34817446594388 | 0.0203562340966921 | STK24//RNF152//FAS//PRKAA2//XBP1//ZC3H12A//PCK1//OXCT1// |
| GO:0060284 | regulation of cell development | Biological process | 27 | 858 | 393 | 17653 | 1.41352159291091 | 0.0448951563548797 | 0.893017477729563 | 1.34780051159843 | 0.0687022900763359 | SMO//ZNF804A//DYNLT1//GFI1//PMP22//B2M//PTPRG//NF2//YWHAG//DDX6//EIF4E//RARB//TIMP2//NCOA1//TRPC5//STK24//WDR1//ZNF488//SEMA3C//DLL4//CUX2//LTA//IL2//CRTC1//NPPC//TUBB2B//MUL1// |
| GO:0050794 | regulation of cellular process | Biological process | 263 | 11074 | 393 | 17653 | 1.06678573611435 | 0.045215439046874 | 0.895133654202237 | 1.34471324775524 | 0.669211195928753 | KPNA1//DHFR//GFI1//KLF2//ZHX1//IRX2//DR1//CUX2//NR6A1//SMAD5//MITF//DLL4//H2AFY2//PURB//RARB//SMO//TRPS1//UBE2I//CCR5//PSMA8//EREG//IL2//TAOK3//PSMA1//PSMC6//STK24//MAP3K8//F2R//NOD1//THBS1//C5AR1//DUSP16//RBM7//NCOA1//BAK1//FKBP1A//ZC3H12A//FAS//CDK2AP1//NF2//STX1A//ATP7A//PGLYRP4//FBXW11//CD209//HAND2//USP16//E2F5//CDK12//CDKL1//PRKAA2//RIPK3//CANT1//ASZ1//FGF14//ZDHHC17//LRRC8B//CD274//PLCXD3//INPP5B//GFRAL//LTA//NPM1//ARHGAP28//CLTCL1//RASSF4//PIP5K1A//GTPBP1//TRAF4//PTPRG//YES1//CLEC4M//ASB15//DGKA//CAMK1G//RGS7//TUFT1//CCDC68//LTBP1//APEX1//PRDX2//PCGF3//KAT7//ZNF597//ZNF558//ZNF320//KDM2A//ACAD8//TAF5L//ZNF544//ZNF699//AFF3//NFYC//YBX2//ATRX//COMMD8//ARNTL2//REXO4//RORA//TAF11//BTF3//ZNF37A//ZNF75D//CSDE1//ZNF329//SAP30L//LZTR1//ZNF251//FSTL3//ZNF273//SUB1//WDHD1//FOXP4//CKS2//CERS6//GABPB1//ALX3//QRICH1//THAP10//RFX4//ZNF704//HIF3A//SP4//SLTM//CREB5//DDX6//EIF4E//EIF5//NCBP2//OGFOD1//YWHAG//MUL1//TRIAP1//PLAGL1//XBP1//DHCR24//HEPACAM//CUL2//CDC14A//TSPAN5//TSPAN19//TSPAN12//CALCR//CD3G//NPPC//TPRA1//F2RL2//OR2L13//HTR1F//PDE1B//GRIK2//TMEM100//DZIP1//G3BP2//CD40LG//RAB6C//TRPC3//PAX7//UFM1//PRAMEF19//DYNLT1//RGS5//SERPINB3//TRPC5//SPEG//BTG3//ASPH//PMP22//BCHE//TIMP2//RPE65//ATP6V0E1//WDR1//TRIM71//CEP135//SERPINB4//CMTM7//RNF152//USP51//WIZ//LMAN1//VDAC1//CYP19A1//ZNF804A//B2M//PIP5K1C//LARP4B//UPF3A//EXOC8//PPP1R13B//ALX4//EBF3//CRLF3//RNF4//NCOA4//FUBP3//PIAS2//CHURC1//ZNF488//MEX3B//SYT16//BTN2A2//BTN2A1//HLA-DPB1//TCTEX1D2//SEMA3C//MCAM//OSBPL8//RAP2C//CTDSPL2//TFRC//RUNDC1//MOB1B//RASSF2//MTM1//RAB36//CRTC1//VNN1//ARPC5//OXCT1//VSNL1//NUS1//FAM20C//CD1D//AEN//NECAB3//NECAB1//CD200//GZMK//MAPKBP1//GCLM//PEX5L//HOXA9//CCNC//PBX3//MIXL1//MAP9//KCNC4//PIRT//SYP//SHISA6//SHISA9//WDR48//C14ORF39//NOVA2//DCUN1D5//OTUD3//ACPP//SNAP47//SLC24A2//CCSAP//IFI30//SLC30A5//SLC30A2//QKI//PCK1//EIF3A//KCNS1//TUBB2B//NMD3//NBPF3//SYNJ1//RAD52// |
| GO:0099537 | trans-synaptic signaling | Biological process | 22 | 670 | 393 | 17653 | 1.47493828567088 | 0.0453780358264898 | 0.895133654202237 | 1.34315430633954 | 0.0559796437659033 | STX1A//GRIK2//HTR1F//KCNC4//KCNQ3//PMP22//KIF1B//VDAC1//PIP5K1C//SYT16//SNAP47//SYNJ1//FGF14//YWHAG//SYP//SHISA6//SHISA9//BCHE//SLC24A2//F2R//CRTC1//CUX2// |
| GO:0000082 | G1/S transition of mitotic cell cycle | Biological process | 11 | 276 | 393 | 17653 | 1.79023306412951 | 0.045572489846265 | 0.895133654202237 | 1.34129724315435 | 0.0279898218829517 | DHFR//GFI1//NPM1//TRIAP1//PLAGL1//APEX1//TRIM71//EIF4E//CRLF3//MCM10//CUL2// |
| GO:0061024 | membrane organization | Biological process | 29 | 936 | 393 | 17653 | 1.39170798808203 | 0.0455918729659828 | 0.895133654202237 | 1.34111256612042 | 0.0737913486005089 | SNAP47//STX1A//SYT16//TMEM170A//LYZL6//BAK1//MUL1//CCR5//RUNDC1//COX18//SAMD9L//DYNLT1//THBS1//TIMM9//LMAN1//SYP//OSBPL8//TRIAP1//PPP1R13B//YWHAG//SHISA6//ARPC5//CLTA//DHCR24//PIP5K1C//TFRC//CLTCL1//SYNJ1//CD3G// |
| GO:0042981 | regulation of apoptotic process | Biological process | 45 | 1566 | 393 | 17653 | 1.29076365125325 | 0.0456633314652923 | 0.895133654202237 | 1.34043240681396 | 0.114503816793893 | IL2//NOD1//F2R//FAS//MUL1//DHCR24//NPM1//PAX7//TRIAP1//UFM1//PRKAA2//RARB//PRAMEF19//SMO//PRDX2//THBS1//XBP1//CD40LG//BAK1//ZC3H12A//HAND2//GZMK//LTA//NCOA1//RASSF2//GCLM//GRIK2//GFRAL//C5AR1//MITF//CD3G//RIPK3//PPP1R13B//YWHAG//VNN1//CD274//CCR5//MAP3K8//ALX3//APEX1//NF2//TAOK3//ALX4//STK24//TRAF4// |
| GO:0016079 | synaptic vesicle exocytosis | Biological process | 5 | 88 | 393 | 17653 | 2.55219176497802 | 0.0466169434332546 | 0.900361717489385 | 1.3314562055814 | 0.0127226463104326 | STX1A//SNAP47//SYNJ1//SYT16//PIP5K1C// |
| GO:0046794 | transport of virus | Biological process | 4 | 61 | 393 | 17653 | 2.94548033204021 | 0.0468151219578095 | 0.900361717489385 | 1.32961384089941 | 0.0101781170483461 | DYNLT1//CLEC4M//CD209//KPNA1// |
| GO:1902110 | positive regulation of mitochondrial membrane permeability involved in apoptotic process | Biological process | 4 | 61 | 393 | 17653 | 2.94548033204021 | 0.0468151219578095 | 0.900361717489385 | 1.32961384089941 | 0.0101781170483461 | PPP1R13B//YWHAG//MUL1//BAK1// |
| GO:0002694 | regulation of leukocyte activation | Biological process | 18 | 525 | 393 | 17653 | 1.54006543075245 | 0.0471675561204865 | 0.900361717489385 | 1.32635662515101 | 0.0458015267175573 | IL2//TFRC//MAP3K8//CD274//HLA-DPB1//YES1//CD3G//CD40LG//VNN1//CD209//CD1D//RORA//CD200//ZC3H12A//THBS1//XBP1//RIPK3//BTN2A2// |
| GO:0006913 | nucleocytoplasmic transport | Biological process | 13 | 347 | 393 | 17653 | 1.68282846059646 | 0.0477428511289798 | 0.900361717489385 | 1.32109164916232 | 0.0330788804071247 | NPM1//NMD3//EIF4E//NCBP2//SRSF11//KPNA1//TRPS1//SMO//ZC3H12A//DUSP16//CTDSPL2//SNRPF//UPF3A// |
| GO:0007346 | regulation of mitotic cell cycle | Biological process | 22 | 674 | 393 | 17653 | 1.46618494272922 | 0.0478548905317414 | 0.900361717489385 | 1.32007367288364 | 0.0559796437659033 | NPM1//TRIAP1//PLAGL1//TAOK3//CDC14A//YWHAG//CEP135//PSMA8//PSMA1//PSMC6//EREG//BTG3//TIMP2//EIF4E//MAP9//APEX1//CCSAP//TPRA1//ATRX//CKS2//MAP3K8//STK24// |
| GO:0045664 | regulation of neuron differentiation | Biological process | 20 | 600 | 393 | 17653 | 1.49728583545377 | 0.0481728044734494 | 0.900361717489385 | 1.31719806965215 | 0.0508905852417303 | ZNF804A//DYNLT1//GFI1//PMP22//B2M//PTPRG//DDX6//EIF4E//RARB//TIMP2//NCOA1//TRPC5//STK24//SEMA3C//CUX2//IL2//CRTC1//TUBB2B//MUL1//YWHAG// |
| GO:0070663 | regulation of leukocyte proliferation | Biological process | 9 | 212 | 393 | 17653 | 1.90692063949301 | 0.0482568016687721 | 0.900361717489385 | 1.31644146534544 | 0.0229007633587786 | IL2//TFRC//CD274//CD209//HLA-DPB1//CD1D//CD40LG//RIPK3//BTN2A2// |
| GO:0010155 | regulation of proton transport | Biological process | 2 | 16 | 393 | 17653 | 5.61482188295165 | 0.0482932294454539 | 0.900361717489385 | 1.31611375166231 | 0.00508905852417303 | ATP7A//SLC30A5// |
| GO:0031629 | synaptic vesicle fusion to presynaptic active zone membrane | Biological process | 2 | 16 | 393 | 17653 | 5.61482188295165 | 0.0482932294454539 | 0.900361717489385 | 1.31611375166231 | 0.00508905852417303 | SNAP47//STX1A// |
| GO:0034755 | iron ion transmembrane transport | Biological process | 2 | 16 | 393 | 17653 | 5.61482188295165 | 0.0482932294454539 | 0.900361717489385 | 1.31611375166231 | 0.00508905852417303 | ATP7A//STEAP2// |
| GO:0048535 | lymph node development | Biological process | 2 | 16 | 393 | 17653 | 5.61482188295165 | 0.0482932294454539 | 0.900361717489385 | 1.31611375166231 | 0.00508905852417303 | RIPK3//LTA// |
| GO:0050908 | detection of light stimulus involved in visual perception | Biological process | 2 | 16 | 393 | 17653 | 5.61482188295165 | 0.0482932294454539 | 0.900361717489385 | 1.31611375166231 | 0.00508905852417303 | RPE65//CACNA2D4// |
| GO:0050962 | detection of light stimulus involved in sensory perception | Biological process | 2 | 16 | 393 | 17653 | 5.61482188295165 | 0.0482932294454539 | 0.900361717489385 | 1.31611375166231 | 0.00508905852417303 | RPE65//CACNA2D4// |
| GO:0071428 | rRNA-containing ribonucleoprotein complex export from nucleus | Biological process | 2 | 16 | 393 | 17653 | 5.61482188295165 | 0.0482932294454539 | 0.900361717489385 | 1.31611375166231 | 0.00508905852417303 | NPM1//NMD3// |
| GO:0099500 | vesicle fusion to plasma membrane | Biological process | 2 | 16 | 393 | 17653 | 5.61482188295165 | 0.0482932294454539 | 0.900361717489385 | 1.31611375166231 | 0.00508905852417303 | SNAP47//STX1A// |
| GO:1902001 | fatty acid transmembrane transport | Biological process | 2 | 16 | 393 | 17653 | 5.61482188295165 | 0.0482932294454539 | 0.900361717489385 | 1.31611375166231 | 0.00508905852417303 | PRKAA2//THBS1// |
| GO:0044238 | primary metabolic process | Biological process | 259 | 10905 | 393 | 17653 | 1.06684190201521 | 0.048595002111222 | 0.900361717489385 | 1.31340839467046 | 0.659033078880407 | KPNA1//KAT7//MCM10//DHFR//GFI1//KLF2//ZHX1//IRX2//DR1//CUX2//NR6A1//SMAD5//MITF//DLL4//H2AFY2//PURB//RARB//SMO//TRPS1//UBE2I//CCR5//PSMA8//EREG//IL2//TAOK3//PSMA1//PSMC6//STK24//NCBP2//UPF3A//MAP3K8//F2R//NOD1//THBS1//C5AR1//DUSP16//FBXW11//UBE2W//EIF4E//ZC3H12A//COPS7B//DBR1//RBM7//SNRPF//NOVA2//RNPC3//SRSF11//FKBP1A//NCOA1//RPL7L1//APEX1//WDR48//RAD52//EXOG//RPE65//LRAT//POP4//EIF3A//FAS//CDK2AP1//ATP7A//SUB1//EBF3//TAF5L//NFYC//NPM1//PBX3//YBX2//PLAGL1//RFX4//ALX4//HIF3A//TAF11//BTF3//XBP1//MIXL1//FUBP3//HAND2//CREB5//ASB15//WDSUB1//RNF152//RNF4//MUL1//CUL2//YDJC//ALDH1B1//HYAL4//RPE//ALDOB//PCK1//H6PD//MOGAT3//COX4I1//ACPP//CMPK1//NPPC//PDE1B//WDHD1//USP51//ATRX//APEX2//KDM2A//TSN//FSTL3//PCGF3//USP16//ZNF273//FOXP4//ZNF488//ZNF597//ZNF558//ZNF320//E2F5//CRTC1//GABPB1//ALX3//ACAD8//ZNF544//HOXA9//ZNF699//AFF3//PAX7//COMMD8//PRKAA2//ARNTL2//SP4//ZNF37A//ZNF75D//ZNF329//SAP30L//SLTM//NCOA4//PIAS2//ZNF251//CHURC1//REXO4//RORA//CSDE1//LZTR1//CKS2//CERS6//QRICH1//THAP10//ZNF704//SENP3//CCNC//CDK12//QKI//MRPS7//COA1//EIF1AX//EIF5//DDX3Y//GTPBP1//DDX6//OGFOD1//RIPK3//YES1//SEC11A//SPEG//FAM20C//MEX3B//CDKL1//RASSF2//NF2//YWHAG//MTM1//CTDSPL2//PTPN7//PCMTD1//ASPH//NUS1//XPNPEP2//VNN1//C2ORF66//CPQ//GZMK//NRIP3//PRSS22//ST14//XPNPEP1//CTSF//ADAM23//NGLY1//GCLM//MCCC2//BTN2A1//TPRA1//ABCD3//SCD//AGPAT5//ETNK2//PIP5K1A//DGKA//AGPAT4//PIP5K1C//SYNJ1//CYP19A1//DHCR24//IDI2//BAK1//TRAF4//G3BP2//CD40LG//MAPKBP1//GALNT14//RFK//TRIM71//SERPINB3//SERPINB4//WIZ//C14ORF39//TRPC5//TIMP2//PLCXD3//LARP4B//EBPL//CRLF3//PRAMEF19//TEX30//OTUD3//CAMK1G//LMAN1//ZDHHC17//CPOX//ENOPH1//NECAB3//DCUN1D5//CANT1//TMEM100//CALCR//MOB1B//RAP2C//OSBPL8//CLTA//CRAT//ASZ1//EXD1//PTPRG//PTP4A2//CDC14A//NECAB1//TRIAP1//SUMF2//LTBP1//VWA1//B2M//TFRC//GRIK2//INPP5B//SULT6B1//TSPAN5//UFM1//N4BP2//AEN//PGBD5//NMD3//TMEM199// |
| GO:0042149 | cellular response to glucose starvation | Biological process | 3 | 37 | 393 | 17653 | 3.64204662677945 | 0.0487257709006528 | 0.900361717489385 | 1.3122412810918 | 0.00763358778625954 | PRKAA2//XBP1//ZC3H12A// |
| GO:0042596 | fear response | Biological process | 3 | 37 | 393 | 17653 | 3.64204662677945 | 0.0487257709006528 | 0.900361717489385 | 1.3122412810918 | 0.00763358778625954 | EIF4E//GRIK2//VDAC1// |
| GO:0046839 | phospholipid dephosphorylation | Biological process | 3 | 37 | 393 | 17653 | 3.64204662677945 | 0.0487257709006528 | 0.900361717489385 | 1.3122412810918 | 0.00763358778625954 | INPP5B//MTM1//SYNJ1// |
| GO:0009987 | cellular process | Biological process | 369 | 16158 | 393 | 17653 | 1.02580481485786 | 0.0490151635114298 | 0.903241282909536 | 1.30966954424518 | 0.938931297709924 | KPNA1//MRPS7//KAT7//MCM10//UBE2I//DDX3Y//TUBB2B//TRIM71//EIF4E//CRLF3//CUL2//DHFR//GFI1//FBXW11//YWHAG//CEP135//KLF2//ZHX1//IRX2//DR1//CUX2//NR6A1//SMAD5//MITF//DLL4//H2AFY2//PURB//RARB//SMO//TRPS1//DYNLT1//CCR5//PSMA8//EREG//IL2//TAOK3//PSMA1//PSMC6//STK24//NCBP2//UPF3A//MAP3K8//F2R//NOD1//THBS1//C5AR1//DUSP16//UBE2W//ATRX//DYNC1LI2//TACC1//MUL1//USP16//WDHD1//CLTCL1//RAB6C//ZC3H12A//COPS7B//DBR1//RBM7//SNRPF//NOVA2//RNPC3//SRSF11//FKBP1A//NCOA1//RPL7L1//APEX1//WDR48//RAD52//EXOG//CDH24//MAP9//RPE65//LRAT//TMEM100//QKI//POP4//EIF3A//SEMA3C//BAK1//EXOC8//FAS//CDK2AP1//NF2//STX1A//ATP7A//PGLYRP4//CD209//FSTL3//AGPAT5//MIXL1//CMTM7//WDR1//FGF14//GJB2//LTA//HAND2//SPAG17//NPPC//SUB1//EBF3//TAF5L//NFYC//NPM1//PBX3//YBX2//PLAGL1//RFX4//ALX4//HIF3A//TAF11//BTF3//XBP1//FUBP3//CREB5//E2F5//CDK12//CDKL1//LMAN1//NGLY1//RGS7//TBCA//B2M//ARPC5//PIP5K1C//SYNJ1//QRICH1//DHCR24//RNF152//KIF1B//GSDMA//EMC4//TRIAP1//PDE1B//RFK//VDAC1//SLTM//TRAF4//PRKAA2//RIPK3//CANT1//ASZ1//ZDHHC17//LRRC8B//CD274//PLCXD3//INPP5B//GFRAL//ARHGAP28//RASSF4//PIP5K1A//GTPBP1//PTPRG//YES1//CLEC4M//ASB15//DGKA//CAMK1G//TUFT1//CCDC68//LTBP1//SNAP47//SYT16//WDSUB1//RNF4//ALDH1B1//MOGAT3//PCK1//COX4I1//ALDOB//RPE//H6PD//PRDX2//CYP19A1//ASPH//ACPP//CMPK1//USP51//APEX2//KDM2A//TSN//PAX7//PCGF3//ZNF273//FOXP4//ZNF488//ZNF597//ZNF558//ZNF320//CRTC1//GABPB1//ALX3//ACAD8//ZNF544//HOXA9//ZNF699//AFF3//COMMD8//ARNTL2//SP4//ZNF37A//ZNF75D//ZNF329//SAP30L//NCOA4//PIAS2//ZNF251//CHURC1//REXO4//RORA//CSDE1//LZTR1//CKS2//CERS6//THAP10//ZNF704//SENP3//CCNC//COA1//EIF1AX//EIF5//DDX6//OGFOD1//SEC11A//SPEG//FAM20C//MEX3B//RASSF2//MTM1//CTDSPL2//PTPN7//PCMTD1//NUS1//XPNPEP2//VNN1//C2ORF66//CTSF//GCLM//MCCC2//CPQ//TIMM9//ABCD3//SCD//ETNK2//AGPAT4//CPOX//NAT2//MARC2//SLC24A2//GRIK2//TMEM199//TFRC//SLC30A5//LIX1L//TMEM170A//IGSF22//CLTA//TMED7//ATP6V0E1//TNS1//CCSAP//HEPACAM//SPC24//CDC14A//HEPACAM2//C14ORF39//SYCE1//SNTG1//CD3G//TSPAN5//TSPAN19//TSPAN12//CALCR//TPRA1//F2RL2//OR2L13//HTR1F//TRPC5//DZIP1//G3BP2//CD40LG//KCNC4//KCNQ3//PMP22//GLIPR1L2//LYZL6//KERA//TRPC3//CADM2//CD200//UFM1//PRAMEF19//MAPKBP1//RGS5//SERPINB3//BTG3//BCHE//TIMP2//IDI2//COX18//TCTEX1D2//COPG1//NECAB3//SERPINB4//WIZ//XPNPEP1//SLC41A1//ZNF804A//SNX30//LARP4B//SYP//GALNT14//N4BP2//ENOPH1//PPP1R13B//PEX5L//TEX30//OTUD3//CRAT//DCUN1D5//BTN2A2//BTN2A1//HLA-DPB1//MYL1//VWA1//HYAL4//ST14//SFXN1//MCAM//OSBPL8//RAP2C//EXD1//RUNDC1//KRT6C//KRTAP4-4//MOB1B//PGBD5//RAB36//SATL1//SAMD9L//CEP350//GPX3//PYROXD1//PTP4A2//OXCT1//VSNL1//DNALI1//CD1D//AEN//KCNS1//KCTD15//NECAB1//GZMK//SUMF2//STEAP2//PIRT//SHISA6//SHISA9//SULT6B1//SLC9C2//SLC9A2//IFI30//SLC30A2//TCHH//NMD3//NBPF3//LAPTM5//ADAM23// |
| GO:0006401 | RNA catabolic process | Biological process | 14 | 384 | 393 | 17653 | 1.63765638252757 | 0.0494029318198728 | 0.907913119287499 | 1.30624727708018 | 0.0356234096692112 | NCBP2//UPF3A//EIF4E//ZC3H12A//PSMA8//APEX1//PSMA1//PSMC6//DDX6//GTPBP1//TRIM71//QKI//CSDE1//NPM1// |
| GO:0000165 | MAPK cascade | Biological process | 29 | 944 | 393 | 17653 | 1.37991385258981 | 0.0498413381260807 | 0.913392669279243 | 1.30241030637754 | 0.0737913486005089 | STK24//MAP3K8//F2R//TAOK3//NOD1//THBS1//C5AR1//DUSP16//MUL1//CD40LG//FAS//CDK12//NF2//TIMP2//SERPINB3//GRIK2//TRAF4//RASSF2//FBXW11//EIF3A//CALCR//HAND2//ZC3H12A//CCR5//PSMA8//EREG//IL2//PSMA1//PSMC6// |
